# Detection of recurrent alternative splicing switches in tumor samples reveals novel signatures of cancer

**DOI:** 10.1093/nar/gku1392

**Published:** 2015-01-10

**Authors:** Endre Sebestyén, Michał Zawisza, Eduardo Eyras

**Affiliations:** 1 Computational Genomics, Universitat Pompeu Fabra, Dr. Aiguader 88, E08003 Barcelona, Spain; 2 Universitat Politècnica de Catalunya, Jordi Girona 1-3, E08034 Barcelona, Spain; 3 Catalan Institution for Research and Advanced Studies, Passeig Lluís Companys 23, E08010 Barcelona, Spain

## Abstract

The determination of the alternative splicing isoforms expressed in cancer is fundamental for the development of tumor-specific molecular targets for prognosis and therapy, but it is hindered by the heterogeneity of tumors and the variability across patients. We developed a new computational method, robust to biological and technical variability, which identifies significant transcript isoform changes across multiple samples. We applied this method to more than 4000 samples from the The Cancer Genome Atlas project to obtain novel splicing signatures that are predictive for nine different cancer types, and find a specific signature for basal-like breast tumors involving the tumor-driver CTNND1. Additionally, our method identifies 244 isoform switches, for which the change occurs in the most abundant transcript. Some of these switches occur in known tumor drivers, including PPARG, CCND3, RALGDS, MITF, PRDM1, ABI1 and MYH11, for which the switch implies a change in the protein product. Moreover, some of the switches cannot be described with simple splicing events. Surprisingly, isoform switches are independent of somatic mutations, except for the tumor-suppressor FBLN2 and the oncogene MYH11. Our method reveals novel signatures of cancer in terms of transcript isoforms specifically expressed in tumors, providing novel potential molecular targets for prognosis and therapy. Data and software are available at: http://dx.doi.org/10.6084/m9.figshare.1061917 and https://bitbucket.org/regulatorygenomicsupf/iso-ktsp.

## INTRODUCTION

Somatic alterations in the genome can give rise to changes in the transcript isoforms expressed in a cell, thereby affecting multiple functional pathways and leading to cancer (1–4). These transcriptome changes are often reflected as alternative splicing abnormalities in the tumors, which can bear major importance in terms of the understanding and treatment of cancer (5–7). Alternative splicing alterations may confer a selective advantage to the tumor, such as angiogenesis (8), proliferation (9), cell invasion (10) and avoidance of apoptosis (11). These alterations may be caused by somatic mutations (12), but also by changes in expression, amplifications and deletions in splicing factors (13,14). Most genome-wide studies on the role of alternative splicing in cancer have been based on local patterns of splicing changes, encoded as events (15–19). However, alternative splicing takes place through a change in the relative abundance of the transcript isoforms expressed by a gene, which may involve complex patterns not easily described in terms of simple splicing events. Accordingly, to ultimately determine the impact of splicing alterations in cancer, it is important to describe them in terms of transcript isoforms changes, as it has been illustrated previously for TP53 and other genes (20–22). Furthermore, transcript-based analysis has been shown to improve expression-based tumor classification (23,24) and to be essential for proper prognosis and therapy selection (6,7). The determination of the alternative splicing isoforms expressed in tumors is therefore of utmost relevance to uncover novel oncogenic mechanisms and for the development of appropriate prognostic and therapeutic strategies. This task is, however, hindered by the heterogeneity of tumors and the inherent biological variability between patient samples (25,26). There is thus a need for new methods to identify the alternative splicing isoforms expressed in tumors that are robust to variability and that can help expanding and refining the catalog of molecular signatures in cancer.

We have developed a new computational method that is robust to biological and technical variability and which identifies significant transcript isoform changes that are consistent across multiple samples. The method is based on a rank algorithm that detects consistent reversals of relative isoform expression, and is capable of detecting complex alternative splicing changes and isoform switches. We used this method to analyze more than 4000 RNA sequencing (RNA-Seq) samples from The Cancer Genome Atlas (TCGA) project. Using the consistency of the change in relative abundance across samples, our method provides predictive models with a minimal set of isoform pairs that can separate tumor from normal samples in each cancer type and classifies unseen tumor data with high accuracy. The same approach finds a significant signature for basal-like breast tumors that distinguishes them from other breast cancer subtypes and which includes the tumor-driver CTNND1. Additionally, we are able to detect isoform switches, for which the relative expression change occurs in the most abundant isoform, and are therefore more likely to have a functional impact. These switches can also accurately separate tumor and normal samples; they affect genes in pathways frequently altered in cancer and 10 of them occur in known tumor drivers. Surprisingly, most of these switches are independent of somatic mutations, except for the tumor suppressor FBLN2 and the oncogene MYH11, suggesting that recurrent isoform switching in cancer is mostly independent of somatic mutations. Our analyses show that recurrent transcript isoform changes provide novel signatures in cancer that could potentially lead to the development of new molecular targets for prognosis and therapy.

## MATERIALS AND METHODS

### Data collection and processing

Available processed RNA-Seq data for tumor and normal samples were downloaded for nine cancer types (Table 1) (1–4,27–28) together with the University of California, Santa Cruz (UCSC) gene annotation from June 2011 (assembly hg19) and the somatic mutation data from the TCGA data portal (https://tcga-data.nci.nih.gov/tcga/). To assess sample quality, the estimated read-counts per gene were analyzed using Unveiling RNA Sample Annotation (URSA) (29) and sample pairs that did not cluster with the rest of the samples of the same class (tumor or normal) were removed (Supplementary Figure S1). The list of samples kept for further analyses can be found in Supplementary File S1. See Supplementary Material for details.

**Table 1.** Number of analyzed paired and unpaired tumor samples from each cancer type: breast carcinoma (BRCA), colon adenocarcinoma (COAD), head and neck squamous cell carcinoma (HNSC), kidney chromophobe (KICH), kidney renal clear-cell carcinoma (KIRC), lung adenocarcinoma (LUAD), lung squamous cell carcinoma (LUSC), prostate adenocarcinoma (PRAD) and thyroid carcinoma (THCA)

TCGA acronym	Cancer type	Paired samples	Unpaired tumor samples	Reference
BRCA	Breast invasive carcinoma	107	929	(3)
COAD	Colon adenocarcinoma	26	236	(1)
HNSC	Head/neck squamous cell carcinoma	38	384	(27)
KICH	Kidney chromophobe	21	41	https://tcga-data.nci.nih.gov/
KIRC	Kidney renal clear cell carcinoma	71	434	(4)
LUAD	Lung adenocarcinoma	57	431	(28)
LUSC	Lung squamous cell carcinoma	50	433	(2)
PRAD	Prostate adenocarcinoma	48	247	https://tcga-data.nci.nih.gov/
THCA	Thyroid carcinoma	58	439	https://tcga-data.nci.nih.gov/

All data sets were obtained from https://tcga-data.nci.nih.gov/. For the paired samples we also used the corresponding normal samples from the same patients. For the list of samples used see Supplementary File S1.

The abundance of every transcript per sample was calculated in transcripts per million (TPM) (30) from the transcript-estimated read counts provided by TCGA and the isoform lengths from the UCSC (June 2011) annotation. No further normalization on the TPM values was performed. For each transcript, the relative abundance (or Percent Spliced In (PSI)) per sample was calculated by normalizing the TPM by the sum of TPMs for all transcripts in the gene. Genes with one single isoform or no Human Genome Organisation (HUGO) ID were not considered for further analysis.

### The iso-kTSP algorithm

Changes in the relative abundance of the alternative transcripts from a gene reflect a variation of their relative order in the ranking of transcript expression. Accordingly, the problem of finding alternative splicing changes in cancer at the transcript level is equivalent to measuring the consistency of the reversals in the relative expression of transcript isoforms from the same gene. For this purpose, we developed the iso-kTSP algorithm, which applies the principle of consistency of expression reversals (31–33) to alternative splicing isoforms. The software is implemented in Java and is available at https://bitbucket.org/regulatorygenomicsupf/iso-ktsp.

**Figure 1.**
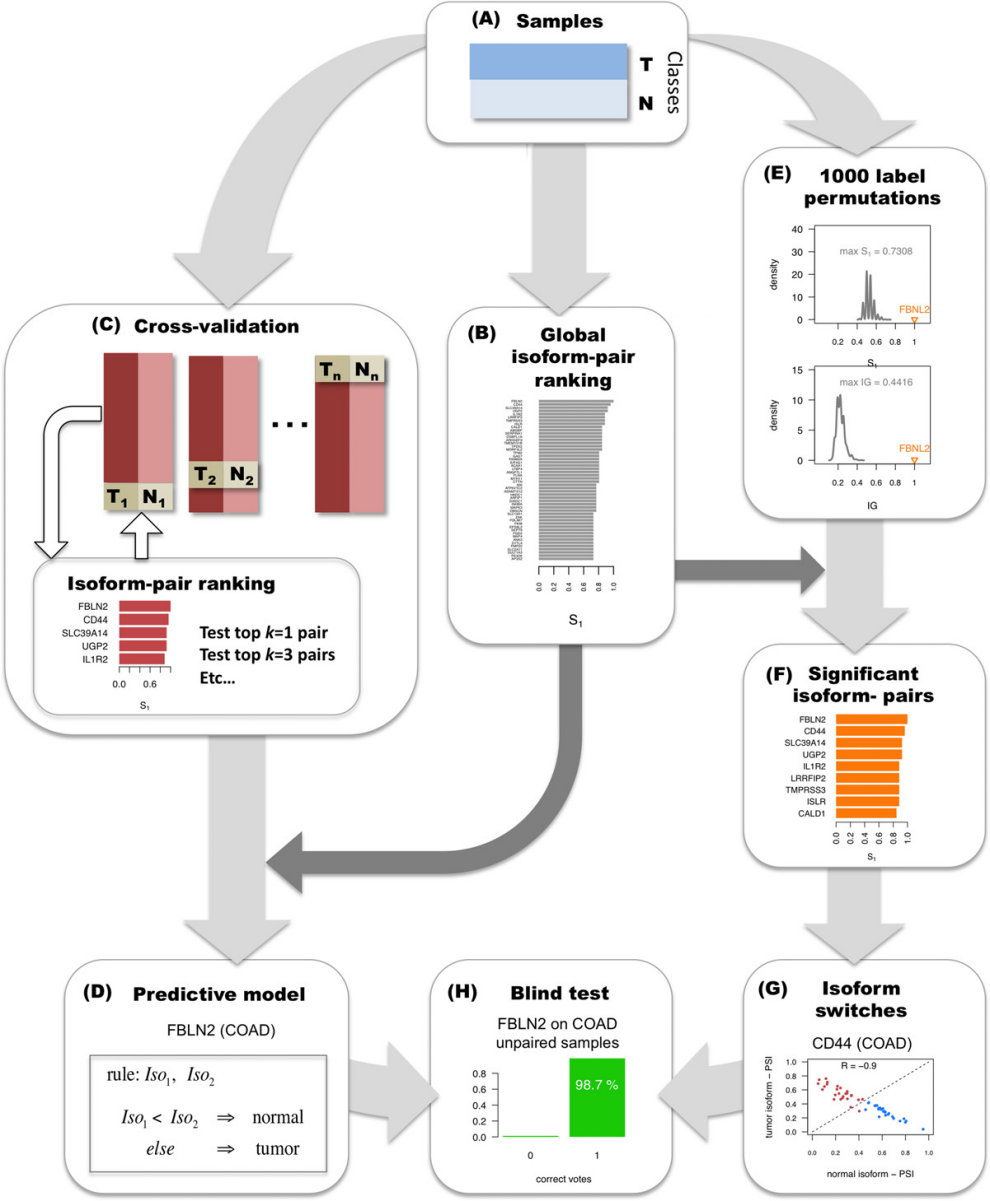
Methodology for detecting significant alternative splicing isoform changes in cancer. The method is illustrated with data from colon adenocarcinoma (COAD). **(A)** Samples are partitioned into two classes, here tumor (T) and normal (N). **(B)** The calculation of relevant isoform-pairs is based on the global ranking of isoform-pairs according to score *S_1_* (Materials and Methods). **(C)** Predictive models are obtained by performing cross-validation: iteratively training in all but one pair of tumor-normal samples, and testing on this left-out pair. At each step of the cross-validation, the top *k* = 1, 3, 5, etc. isoform-pairs of the score *S_1_* ranking are tested on the left-out sample pair according to a majority voting (Materials and Methods). **(D)** A minimal classification model is obtained by selecting the smallest number of pairs from the global ranking with the largest average accuracy calculated in the cross-validation. In the case of COAD, this model consists of a single isoform-pair model in gene FBLN2. **(E)** Significance of the isoform-pairs is assessed by comparing to the expected distributions of score *S_1_* and *IG* values obtained from 1000 permutations of the class labels and by selecting at each permutation the highest score *S_1_* and the highest *IG*. **(F)** The result from the permutation analysis is a ranking of significant isoform-pairs that change relative expression between tumor and normal samples more than expected by chance. **(G)** From this ranking of significant isoform-pairs, we detect as isoform switches those isoform-pairs with minimum score and expression value that anti-correlate across samples (Materials and Methods). In the example, CD44 presents a clear switch between two isoforms in COAD even though it was not chosen in the minimal classification model. **(H)** The isoform-pairs (either from the minimal classification model or from the set of isoform switches) are tested on a held-out data set of unpaired tumor samples.

The iso-kTSP algorithm is based on the following calculation. Given the ranking of isoform expression from multiple samples separated into two classes (Figure 1A), all possible isoform-pairs from the same gene are then sorted according to the sum of frequencies of the two possible relative orders occurring separately in each class, defined as score *S*_1_ (Figure 1B). That is, for every pair of isoforms *I_g,i_* and *I_g,j_* in each gene *g, S*_1_ is based on the frequencies of the two possible relative orders in classes *C_m_, m* = 1,2:

\begin{equation*} S_1 (I_{g,i} ,I_{g,j} ) = P(I_{g,i} {>} I_{g,j} |C_1 ) + P(I_{g,i} {<} I_{g,j} |C_2 ) - 1 \end{equation*}

where *P*(*I_g,i_* > *I_g,j_* |*C*_1_) and *P*(*I_g,i_* < *I_g,j_* |*C*_2_) are the frequencies at which the isoform *I_g,i_* appears later than, or before, *I_g,j_* in the expression ranking of classes *C*_1_ or *C*_2_, respectively. Our definition of *S*_1_ differs from the one used in (32) to account for the fact that for RNA-Seq there are many transcripts with zero reads, hence the expression ranking is not always strictly monotonic.

To avoid possible ties, a second score *S*_2_ is used, which is based on the average rank difference per class *C_m_* for each isoform pair, as proposed previously (32) (see Supplementary Methods for details). All possible isoform pairs are then sorted by the *S*_1_ score and in the case of a tie, by the *S*_2_ score. Moreover, only pairs of isoforms from the same gene are considered and only a single pair of isoforms per gene is listed in the ranking of isoform-pairs. The score *S*_1_ provides an estimate of the probability for the isoforms to change relative order between the two classes. The top scoring isoform pairs are therefore the most consistent changes in isoform relative abundance for a gene between two classes, tumor and normal, or between two tumor subtypes. Each one of these isoform-pairs provides a possible classification rule based on the relative expression order. The semantics for an isoform-pair rule is such that if the first isoform has lower expression than the second, the sample is predicted to be *C*_1_, otherwise it is predicted to be *C*_2_. For *C*_1_ = ‘normal’ and *C*_2_ = ‘tumor’:

\begin{equation*} \begin{array}{*{20}l} {{\rm rule}:I_{g,1} ,I_{g,2} } \\ {I_{g,1} {<} I_{g,2} \Rightarrow {\rm normal}} \\ {{\rm else} \Rightarrow {\rm tumor}} \\ \end{array} \end{equation*}

Accordingly, we will call *I_g_*_,1_ the ‘tumor isoform’ and *I_g_*_,2_ the ‘normal isoform’. The classification of a new sample is performed by evaluating each isoform-pair rule against the ranking of isoform expression of this new sample. Given *k* rules, the classifier selects for each isoform-pair rule the class for which the data fulfills the rule. The final decision for classification is established by simple majority voting, by selecting the most voted class from the *k* rules. In order to avoid ties in this voting, predictive models are always chosen with *k* odd. For instance, for *k* = 3:

\begin{equation*} \left. {\begin{array}{*{20}l} {{\rm rule}\,1: \Rightarrow {\rm tumor}} \\ {{\rm rule}\,2: \Rightarrow {\rm tumor}} \\ {{\rm rule}\,3: \Rightarrow {\rm normal}} \\ \end{array}} \right\}{\rm classification:tumor} \end{equation*}

The optimal number *k* of isoform pairs in the classifier, *k*_opt_, is calculated by performing cross-validation on the training set (Figure 1C). The ranking of isoform-pairs is calculated at each iteration step on a balanced set leaving out one sample from each class, which are used for testing (Figure 1C). The prediction class for a new sample is obtained by evaluating the expression ranking in the new sample against the isoform pair rules. At each iteration step in the cross-validation, the top *k*-pairs (*k* = 1… *k*_max_, with *k* odd) are evaluated on the test set. For each *k*, the accuracy of the model is evaluated against the test set:

\begin{equation*} {\rm accuracy} = \frac{{TP + TN}}{{TP + TN + FN + FP}}, \end{equation*}

where TP, TN, FN and FP are the true positives, true negatives, false negatives and false positives, respectively. This accuracy value is symmetric with respect to the choice of either class as reference for positive cases. Additionally, iso-kTSP also reports the discriminating power of each isoform-pair rule in terms of the information gain (IG). IG provides an estimate of the predictive power of each individual isoform pair and is calculated in terms of the samples that are correctly and incorrectly classified according to the isoform-pair rule (see Supplementary Methods for details). From the global ranking (Figure 1B) we then select the top *k*_opt_ isoform-pairs as a minimal predictive model (Figure 1D), where *k*_opt_ is the smallest odd number of isoform-pairs that have the highest average performance obtained in the cross-fold validation.

### Significance

Significance of the isoform-pairs is measured by performing 1000 permutations of the sample labels (Figure 1E). At each permutation, the cross-fold validation is run as before but keeping only the pair with the highest score *S*_1_. An isoform-pair is significant if its score *S*_1_ and IG are larger than the maximum ones obtained from the permutation analysis. The global ranking of isoform-pairs (Figure 1B) together with the permutation analysis (Figure 1E) yields the list of significant isoform-pairs (Figure 1F).

### Isoform switches

Among the significant isoform-pairs (Figure 1F), those for which the relative expression change occurs in the most abundant isoform of the gene, i.e. isoform switch, have potential functional relevance. We detect these isoform switches from the list of significant isoform-pairs by imposing an anti-correlation (Spearman *R* < −0.8) filter on the relative inclusion levels or PSIs of the isoforms, and keeping those pairs with score *S*_1_ > 0.5 and with average expression per isoform of >1 TPM across either tumor or normal samples (Figure 1G).

### Blind tests

To assess the accuracy of the minimal classification model, or that of a set of isoform switches, a blind test is carried out on the samples not used for cross-validation, for which we measure the proportion of samples correctly labeled by the classifier, as well as the number of correct votes for each prediction (Figure 1H).

### Comparison with other approaches

The performance of the derived isoform-pair models was compared to the performance of the models based on the expression reversals of genes using the kTSP algorithm (32), which is implemented as an option in iso-kTSP. Using the same input data sets, we calculated the gene expression as the sum of the expression of all its transcripts. Additionally, as a validation of our isoform-pairs, we compared our results with those predicted with SwitchSeq (http://biorxiv.org/content/early/2014/06/06/005967) on the same input data sets (Supplementary Table S1).

### Mutation association analysis

For the purpose of finding associations between somatic mutations and isoform switches, we considered those samples for which we had both RNA-Seq and mutation data from DNA sequencing (Supplementary Table S2). Using these samples, we compared the number of samples with a given isoform switch with the number of samples for which the transcripts involved in the switch overlap mutations. Given the samples *M* with one or more mutations in either isoform from the pair, and the samples *S* with the isoform switch, a Jaccard index *J* for the association of these two variables was calculated as:

\begin{equation*} J = \frac{{|M \cap S|}}{{|M \cup S|}}, \end{equation*}

which takes values between 0 and 1. For each isoform switch in each cancer type, a *z*-score was calculated by comparing its value *J* to the *J* values of 100 genes with similar median isoform length. The above analysis was also repeated using only mutations that affect the protein sequence or considering the overlap with genes rather than transcript regions, obtaining similar results (see Supplementary Methods). The mutual information for the association of isoform switches and mutations, and corresponding *z*-score were also computed (see Supplementary Methods). To measure the association of mutations to isoform PSI values, the distribution of the differences between tumor and normal isoform PSIs was compared between mutated and non-mutated samples using a Mann–Whitney test. On the other hand, to measure the mutual exclusion between isoform switches and protein-affecting mutations, we used the following approach: given the number of samples having an isoform switch and no mutation (*n*_10_), and those having a mutation but no isoform switch (*n*_01_), a mutual-exclusion score (mx), with values between 0 and 1, was defined as:

\begin{equation*} mx = 2\frac{{\min (n_{10} ,n_{01} )}}{N}, \end{equation*}

where *N* is the total number of samples. A *z*-score was calculated similarly as above (see Supplementary Methods). Further details and data are provided as Supplementary Material.

## RESULTS

### Recurrent alternative splicing isoform changes can separate tumor and normal samples

**Figure 2.**
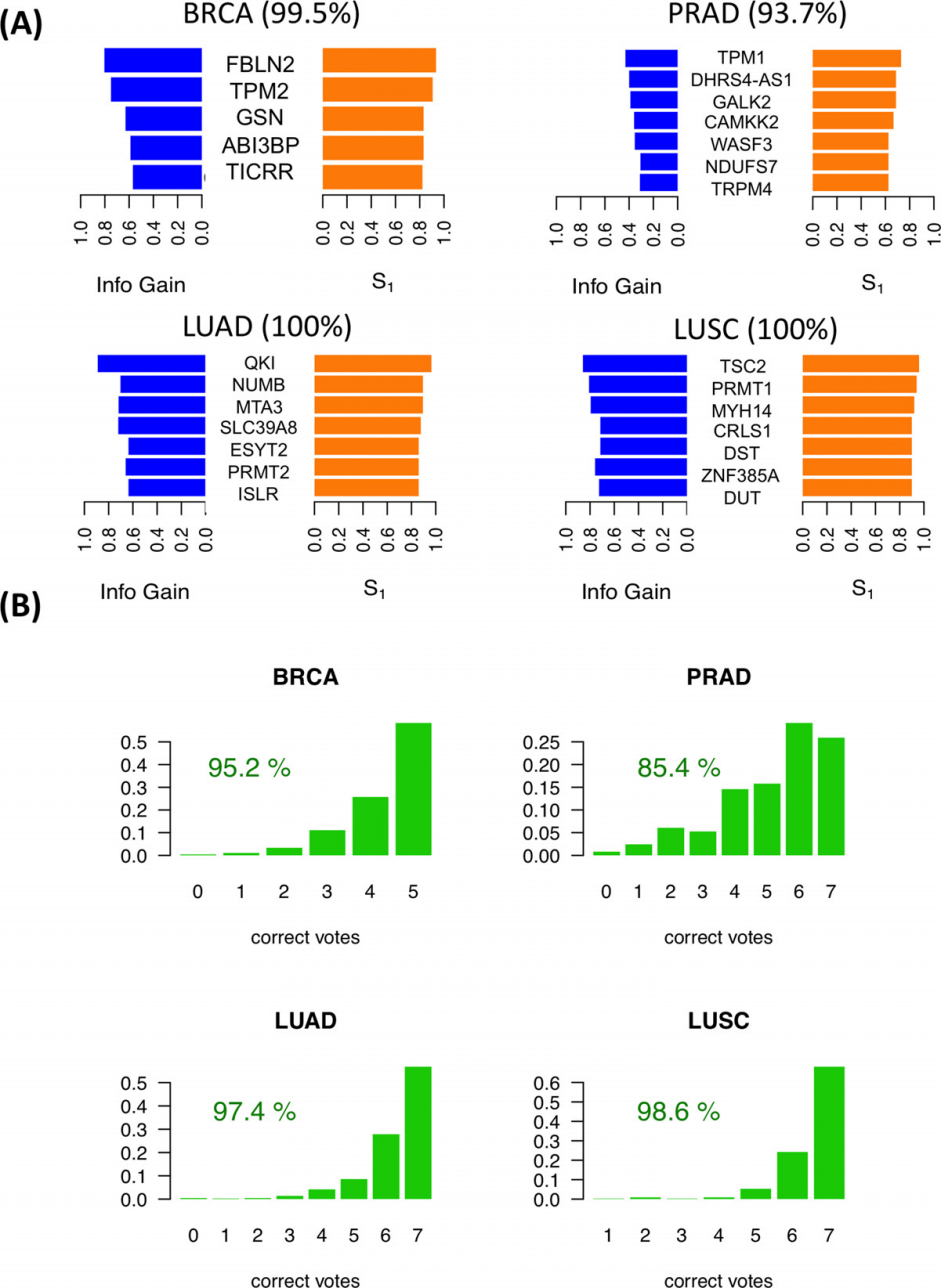
Predictive isoform-pair models. **(A)** Minimal isoform-pair classifiers for BRCA, PRAD, LUAD and LUSC (models for KICH, KIRC, HNSC and THCA are given in Supplementary Figure S2). Each panel shows the score *S_1_* and IG for each isoform-pair in the model, which is indicated by the gene symbol. All isoform-pairs are significant according to the permutation analysis. Next to each cancer label the maximum expected accuracy is given, which is calculated from the cross-validation analysis. Plots with the expression values for each isoform pair are provided in Supplementary Figures S3–S8. **(B)** Blind tests of the isoform-pair models on the unpaired samples for each cancer type. The barplots indicate the proportion of samples (*y*-axis) for each possible number of isoform-pair rules from the model fulfilled by the tumor samples (*x*-axis). A sample is labeled according to a majority vote from all isoform-pair rules. The percentage of samples correctly labeled is also given.

For each cancer type, the iso-kTSP algorithm was applied to the paired samples to obtain minimal classifiers to separate tumor and normal samples. This yielded different predictive models for the 9 cancer types (Figure 2A and Supplementary Figure S2A), with PRAD, THCA and KIRC having the lowest average accuracies, and lung squamous cell carcinomas (LUSC), LUAD, COAD and KICH achieving 100% average accuracy in the cross-fold validation. The blind tests on the remaining unpaired tumor samples show overall accuracies greater than 84% (Figure 2B and Supplementary Figure S2B). These models provide a minimal set of isoform-pairs whose relative expression can separate tumor and normal samples with high accuracy despite the variability of the transcript expression measurement across samples (Supplementary Figures S3–S8) (model files are given in Supplementary File S2). All the isoform-pairs derived for the models are significant according to the permutation analysis (Supplementary Figures S9). This significance depends in general on the number of samples available and on the heterogeneity of the tumor samples. Permutation analysis for a varying number of input samples indicates that in order to obtain significant isoform changes, more than 13 samples are needed on average (Supplementary Figure S10), which is the case for the cancer types analyzed.

**Figure 3.**
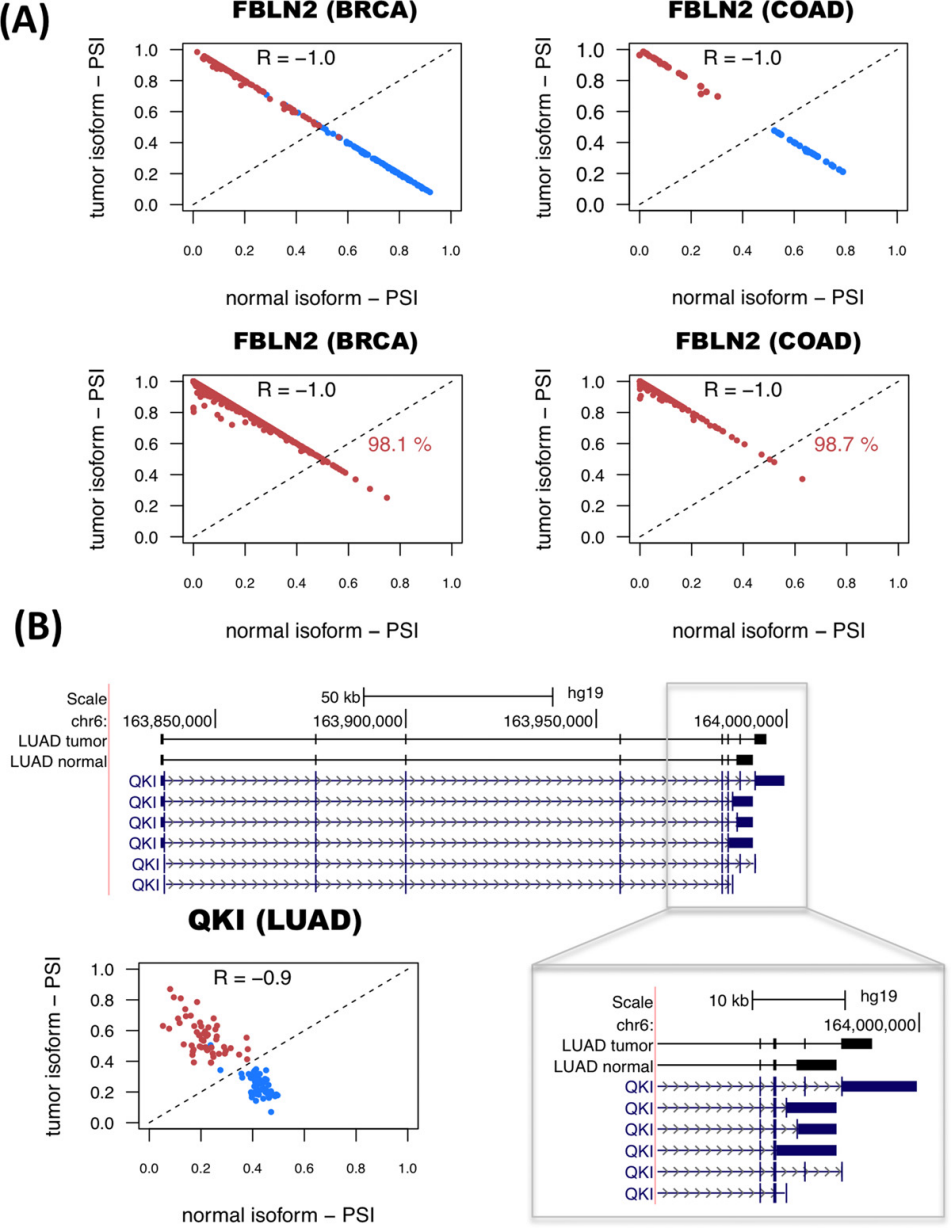
Examples of predictive isoform-pairs. **(A)** The relative inclusion values (PSIs) for the isoform-pair detected for FBLN2 separate tumor from normal samples in BRCA and COAD (upper panels). The *x*-axis represents the PSI for the isoform found to be more abundant in normal samples (normal isoform) and the *y*-axis represents the PSI of the most abundant isoform in tumor samples (tumor isoform). Tumor and normal samples are shown in red and blue, respectively. The bottom panels show the PSIs for the unpaired samples, and the percentage of correctly labeled tumor samples by this isoform-pair is indicated. **(B)** Significant isoform-pair change for QKI in LUAD. The gene locus of QKI is shown, indicating the exon-intron structures of the most abundant isoforms in tumor and normal samples. The zoom-in highlights the 3′-end region where the splicing variation takes place. The bottom left panel shows the PSI values for the normal (*x*-axis) and tumor isoforms (*y*-axis). As before, normal and tumor paired samples are shown in blue and red, respectively.

The genes with significant isoform-pairs detected include FBLN2, which undergoes an isoform change related to the skipping of a protein coding exon (Supplementary Figure S11) and moreover appears as a single gene model for COAD and is part of the BRCA model (Figure 3A). FBLN2 has been proposed before to be a tumor suppressor (34) with a cancer-related function that seems to be specific of the protein produced in tumor cells (35). This isoform switch occurs in more than 98% of the unpaired tumor samples in BRCA and COAD (Figure 3A). In the case of LUAD, surprisingly, we found that the most informative isoform change does not occur in NUMB, as reported previously using microarrays (9,17), but in the splicing factor QKI, which shows a change that cannot be described in terms of a simple alternative splicing event (Figure 3B). In contrast, the LUSC model involves a different set of genes from LUAD model, and includes the gene ZNF385A (Figure 2), whose protein product interacts with TP53 and promotes growth arrest (36). The isoform change found is related to the use of an alternative first exon (Supplementary Figure S12). Similarly to COAD, THCA and KIRC have single-gene models (Supplementary Figure S2). In particular, for THCA the model involves S100A13, a gene encoding a calcium binding protein that has been proposed to be a new marker of angiogenesis in various cancer types (37). The isoform change involves an alternative first exon and classifies correctly 84.5% of the unpaired tumor samples (Supplementary Figure S13). Interestingly, S100A13 and another member of the S100 family, S100A16, have also isoform changes in KICH, even though they were not included in the KICH model (Supplementary Figure S13). The KIRC model is composed of a single-isoform change involving the production of a transcript with a retained-intron in the gene CPAMD8 that is annotated as non-coding (Supplementary Figure S14). A similar case occurs in the gene NAGS, which is part of the KICH model (Supplementary Figure S2) and is related to an autosomal recessive urea cycle disorder (38). We predict that NAGS produces a protein-coding isoform in normal samples, but in tumor samples it produces an isoform with a retained-intron that is annotated as non-coding (Supplementary Figure S15). Importantly, the loss of the protein coding isoform is predictive of 100% of the KICH tumor samples (Supplementary Figure S15). Other isoform changes are discussed in the Supplementary Material (Supplementary Figures S16–S18). Annotation files (in GFF format) for the isoform-pairs in these models are given in Supplementary File S3.

We compared the predictive accuracy of our isoform-based models with the accuracy of gene-based models using the reversal of gene expression, as described by the original kTSP algorithm (32). We found that both approaches show in general very similar accuracies (Supplementary Figure S19). Interesting exceptions are KIRC and LUSC, for which the isoform-based model shows better accuracy than the gene-based model (compare Supplementary Figure S19 with Supplementary Figure S2). In contrast, KICH shows much better accuracy for gene expression changes than for splicing changes (compare Supplementary Figure S19 with Supplementary Figure S2). Interestingly, the isoform- and gene-based models involve different sets of genes in each cancer type, indicating that these alterations occur through independent mechanisms. This comparison shows that alternative splicing changes can provide independent predictive signatures with similar accuracy to models based on gene expression patterns.

### Changes in alternative splicing isoforms can discriminate tumor subtypes

**Figure 4.**
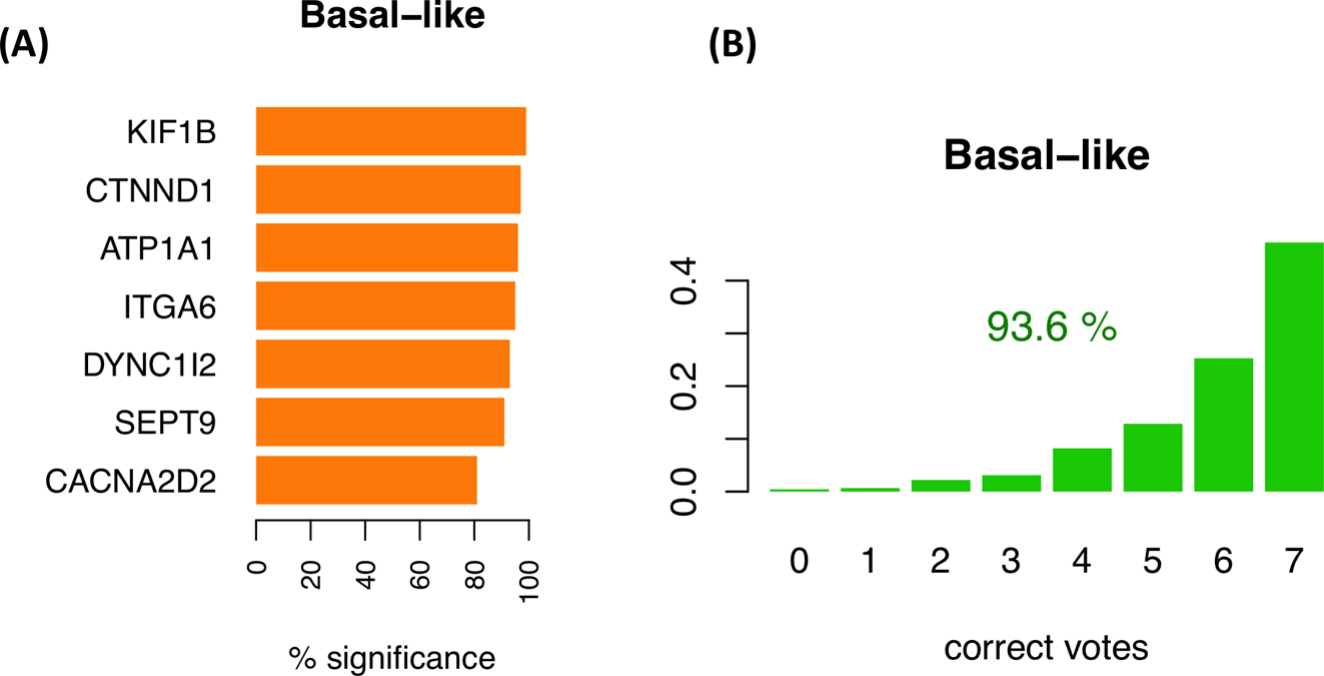
Isoform-pair rules for the basal-like breast tumors. **(A)** The top 7 recurrent isoform changes found comparing basal-like against a balanced pool of the other subtypes (luminal A, luminal B and Her2+). The barplot indicates the frequency of iterations for which the isoform-pair was significant according to the permutation analysis performed on the same subsampled sets. **(B)** Accuracy of the model for the classification of basal-like samples against other subtypes when tested on the entire set of 1036 BRCA tumor samples. The barplot shows the proportion of samples (*y*-axis) with each possible number of correct votes (*x*-axis), from 0 to the number of genes in the model, and the percentage of samples correctly classified.

Cancers are generally classified into subtypes to facilitate patient stratification for more precise prognosis and selection of therapeutic strategy. In particular, breast cancer classification has been recently refined based on molecular information from multiple sources (3). We thus decided to investigate whether breast cancer subtypes are associated with consistent isoform changes when compared to each other. We separated the BRCA tumor samples into luminal A, luminal B, Her2+ and basal-like as labeled by TCGA (3) (Supplementary File S1) and run the iso-kTSP algorithm comparing each subtype against a pool from the rest. In order to maintain balanced sets for the comparison and avoid biases due to sample selection, we subsampled 100 times 45 arbitrary samples for a given subtype and a pool of 15 from each of the other 3 subtypes together. At each iteration step, we performed permutation analysis of the labels to determine the significance of the detected isoform changes. We found that only basal-like tumors showed isoform changes that were significant in more than 80% of the sampling iterations (Figure 4A and Supplementary Figure S20). Among the most significant cases we found KIF1B, which has been implicated in apoptosis (39); ATP1A1, proposed to have tumor suppressor activity (40); ITGA6, found to be required for the growth and survival of a stem cell like subpopulation of MCF7 cells (41); and CTNND1, whose alternative splicing was previously related to cell invasion and metastasis (42) (Figure 4A). We selected the top 7 isoform-pairs in basal-like that were significant in more than 80% of the iterations to build a basal-like model, which classified correctly 93.6% of all the BRCA tumor samples, with 47% of the samples fulfilling all 7 isoform change rules (Figure 4B). Although this cannot be considered a blind test, it provides an estimation of the expected accuracy. For the other BRCA subtypes we found much lower consistency of the isoform changes and none of them were significant for more than 13% of the permutation tests (Supplementary Figure S21).

Four different subtypes have been defined based on mRNA expression for the LUSC: basal, classical, primitive and secretory; which have different clinical and biological characteristics (43). We applied the same approach as above to the four LUSC subtypes using the subtype labeling from TCGA (2), comparing 24 samples from each subtype against the pool of three sets of 8 arbitrary samples from the other subtypes. The most relevant isoform change was found for gene GCNT2 in association to the classical subtype in at least 60% of the subsampling iterations, but significant only in 22% of them (Supplementary Figures S22 and S23). Interestingly, GCNT2 overexpression has been linked to breast and lung cancer metastasis (44). Despite the low recurrence of the isoform changes, tests on the entire data set was able to separate correctly the classical from the other subtypes for more than 80% of samples (Supplementary Figure S22). All other found splicing changes for the other LUSC subtypes occurred at lower frequencies and showed significance in no more than 3% of the iterations.

Colorectal cancers have been classified into hypermutated and non-hypermutated, where non-hypermutated tumors have generally worse prognosis (1). Following previous classification criteria (1), we labeled COAD samples as hyper or non-hyper mutated if they had more or fewer than 250 mutations, respectively, and compared both subtypes by subsampling 40 samples from each one 100 times. This yielded specific isoform changes between the two types occurring in more than 40% of the iterations (Supplementary Figure S24), including a change in the long non-coding RNA gene antisense of NUTM2A (NUTMA2A-AS1), which appeared in 57% of the models. We tested two different models with the top 5 and 13 isoform-pairs, obtaining an accuracy of more than 80% on the total COAD data set (Supplementary Figure S24). Models and annotation files (GFF format) for all subtype models are provided in Supplementary Files S2 and S3, respectively.

### A catalog of alternative isoform switches in cancer

The models described above are optimized to obtain the minimum number of isoform-pairs with maximum average accuracy, which is convenient for defining biomarkers with potential clinical applications. However, the frequency of these isoform changes does not imply functional relevance. On the other hand, cases where the change occurs in the most abundant isoform, i.e. isoform switches (Figure 1G), are more likely to have a functional impact. Accordingly, in order to obtain all the significant isoform changes with a possible functional relevance in cancer, we calculated all significant isoform switches between tumor and normal samples using the following approach: Starting from the 1178 genes with significant isoform changes in at least one cancer type according to our permutation analysis (Figure 1D), we kept those with score *S_1_* > 0.5, which corresponds to selecting isoform-pairs with a change in more than 75% of the samples. Additionally, we kept those cases for which the relative inclusion levels of the two isoforms in the significant isoform-pair anti-correlate, as observed for FBLN2, QKI and other genes (Figure 3 and Supplementary Figures S12–S18). We thus selected those isoform-pairs having an anti-correlation of PSI values of R < −0.8 (Spearman). Finally, we kept only those isoform-pairs with average expression per isoform of >1 TPMs across either tumor or normal samples.

**Figure 5.**
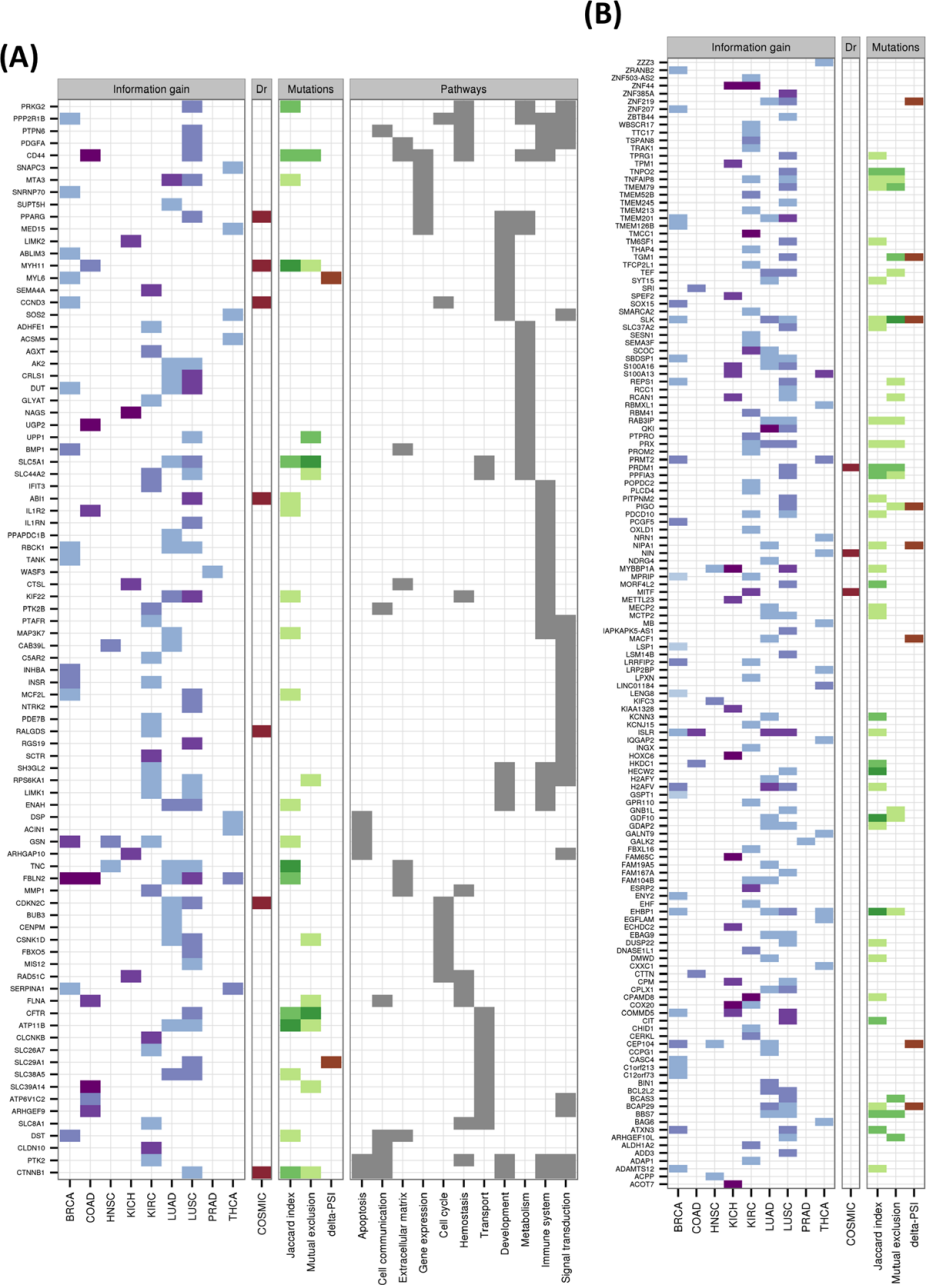
Catalog of isoform switches across various cancer types. Heatmap of the 244 isoform switches detected for the nine cancer types, separated according to whether the genes had an annotated Reactome pathway **(A)** or not **(B)**. The heatmaps show whether the isoform switch occurs in each cancer type, with the color code indicating the IG of the switch: from light blue for low IG (0–0.2) to dark blue/purple for high IG (0.8–1). In red we indicate whether the gene with the switch is annotated as a tumor driver in COSMIC (http://cancer.sanger.ac.uk/). Regarding the mutations, we indicate the Jaccard index and the mutual-exclusion score with light green (0.01–0.02), medium green (0.02–0.03) and dark green (larger than 0.03). The presence of a significant difference (*P*-value < 0.05) of the relative inclusion (delta-PSI) between tumor and normal isoforms in mutated and non-mutated tumor samples before multiple-testing correction is indicated in brown color. The Reactome Pathway annotation for those genes for which this was available is also shown.

This gave a total of 244 isoform switches, with 59 of them appearing in more than one cancer type (Figure 5; Supplementary File S4), and the most common across cancers being FBLN2. From the total 244 switches, 10 occur in known cancer drivers (Figure 5), and several others have been associated before with cancer, like CD44, which has been observed to be relevant in colon cancer initiation (45), and SLC39A14, whose alternative splicing is regulated by WNT in colon cancer (46). LUAD, KIRC and LUSC are the cancer types with most switches, with 85, 65 and 54, respectively. LUSC and LUAD have 33 switches in common. In contrast, KIRC and KICH have only 2 switches in common. HNSC and PRAD are the cancer types with the fewest switches, 7 and 2, respectively. Although functional analysis did not yield any significantly enriched Reactome pathways (47), isoform switches appear frequently in signal transduction, immune system and metabolism related pathways (Figure 5 and Supplementary Figure S25). On the other hand, Gene Ontology analysis shows enrichment of several categories, including actin activity in relation to cell motility and migration, and in categories related to extracellular organization, as well as in response to estrogen and regulation of MAPK activity (Supplementary Figure S26).

**Figure 6.**
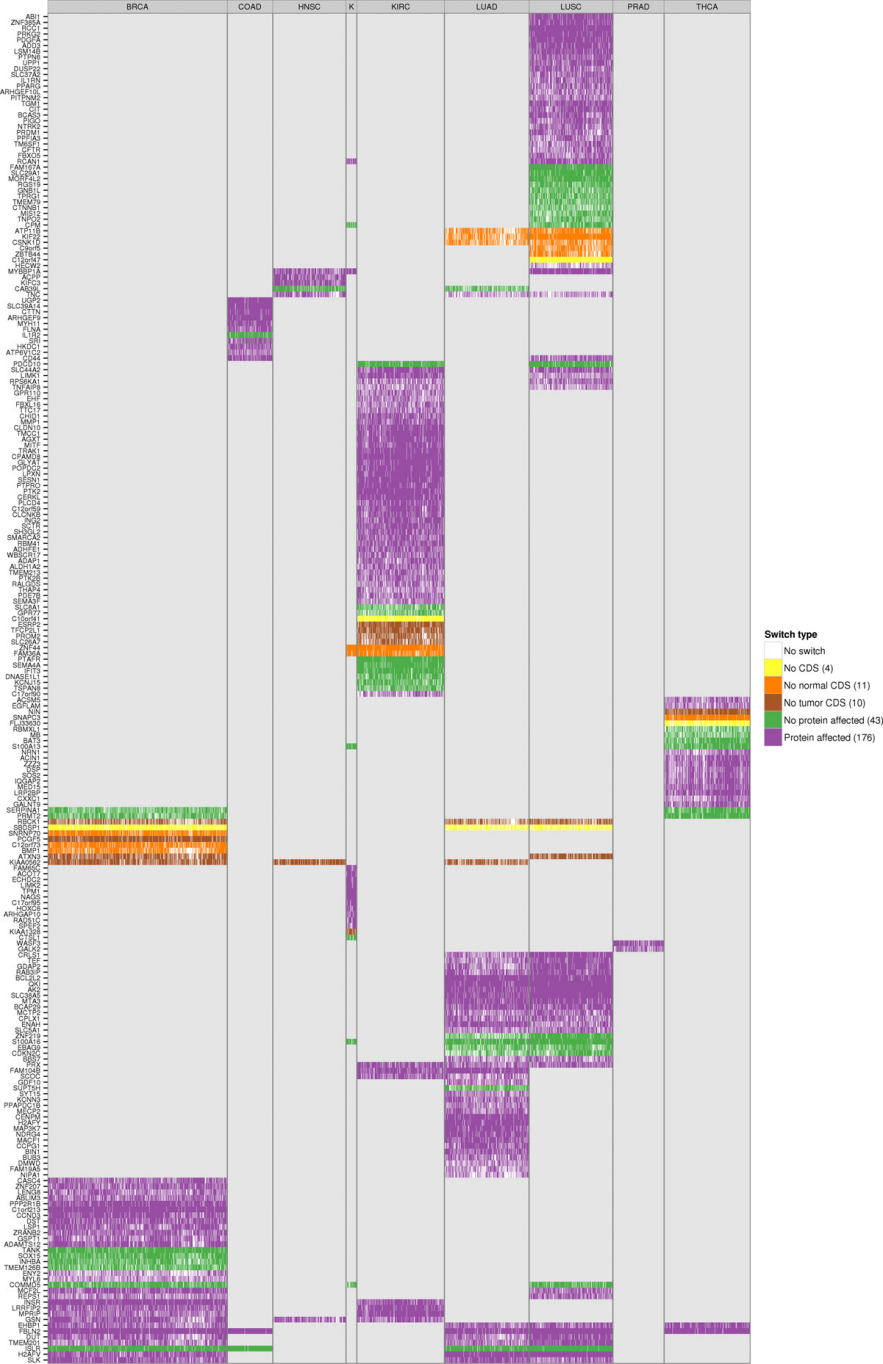
Protein affecting isoform switches across all tumor samples of the nine cancer types. Heatmap of the 244 isoform switches detected in the 9 cancer types, for all paired and unpaired tumor samples. The heatmap shows for each tumor sample whether the switches defined in that cancer type occur in that sample, and whether they affect the protein sequence: *No CDS* means no coding annotation was defined in either the normal or the tumor isoform; *No normal CDS* and *No tumor CDS* means no coding annotation was defined for the normal or the tumor isoform, respectively; *No protein affected* means that the amino-acid sequences are identical for both isoforms in the switch and only UTR regions are differing between the normal and tumor isoform; finally, *Protein affected* means the amino-acid sequence is different between the normal and tumor isoforms. The number in parenthesis on the legend shows the total number of isoform switches for that type. The label text ‘K’ in fourth column refers to the cancer type KICH.

We tested the accuracy of switches as predictive models by performing blind tests with all of them on the set of unpaired tumor samples and found accuracies of around 90% and higher (Supplementary Figure S27). These isoform switches are thus good predictors of tumor samples. Moreover, the majority of the switches involve a change in the encoded protein product (Figure 6): 176 (72%) of the switches affect the protein, 10 (4%) involve a change from coding to non-coding isoform between tumor and normal, 11 (4.5%) involve the reverse change and 43 (17.5%) do not involve any change in the protein sequence (Figure 6). In particular, the tumor drivers PPARG, CCND3, RALGDS, MITF, PRDM1, ABI1 and MYH11, present recurrent isoform switches in LUSC, BRCA, KIRC, KIRC, LUSC, LUSC and COAD, respectively, which affect the encoded proteins and which could have implications for the identification of possible targeted therapies. In contrast, the switches in the tumor drivers CDKN2C and CTNNB1, the former in LUSC and LUAD and the latter in LUSC, do not affect the protein. Interestingly, the tumor driver NIN changes from a protein coding to a non-coding isoform in THCA. These results suggest that the detected alternative splicing switches may have a functional impact in the cancer cells. Interestingly, the significant isoform switches detected present a clearly differential pattern in each cancer type (Figure 6). This raises the question of whether the same switches, even though they are not found to be significant in all cancer types, might still be present in samples from other cancer types at low frequency, but still separate cancer types. To investigate this question, we considered the presence or absence of each isoform switch in each tumor sample by testing the isoform rule corresponding to the switch, regardless of whether the switch was predicted initially in that cancer type. We found that isoform switches indeed group tumor samples together (paired and unpaired) according to cancer type (Supplementary Figure S28). Unsupervised clustering of the occurrence pattern of the switches shows that KIRC, KICH, THCA, PRAD and LUAD tumors are clearly separated from the rest, and that most of the BRCA samples cluster together and next to the COAD samples. In contrast, some LUSC samples are grouped together with LUAD samples, but the majority of them are clustered with HNSC samples (Supplementary Figure S28). These results indicate that isoform switches are not only related to potential tumorigenic processes, but they are also characteristic of specific cancer types. All details for the identified isoform switches and corresponding annotation are provided in Supplementary Files S4 and S5.

To further validate our results, we compared them with the results obtained applying SwitchSeq to the same starting data sets. When we compared our significant isoform-pairs (Figure 1F) with SwitchSeq isoform-pairs, we found that between 25% and 75% of our isoform-pairs are also predicted by SwitchSeq, whereas only 1–26% of the SwitchSeq isoform-pairs are predicted by both methods (Supplementary Table S1A). Moreover, using our score *S*_1_ and the score provided by SwitchSeq, we find overall a low correlation between the common isoform-pairs (Spearman *R* ∼0.17–0.87) (Supplementary Table S1A). Importantly, none of the isoform-pairs predicted by SwitchSeq that are not present in our set are significant according to our permutation test. That is, permuting the tumor and normal labels there is a non-zero probability that the same SwitchSeq isoform-pair will appear by chance. On the other hand, when we compared our isoform switches (Figure 5) with the SwitchSeq results we found that between 90% and 100% of our switches are also predicted by SwitchSeq, and that the correlation between scores for the common isoform-pairs is much higher than before, ranging between 0.75 and 1 (Spearman *R*) (Supplementary Table S1B and Supplementary Figure S29). We conclude that our isoform switches can be independently validated and that they describe changes that are significant as can be distinguished from the variability originating from the heterogeneity of the samples and inter-individual variability.

### Isoform switches in cancer are not frequently associated with somatic mutations

**Figure 7.**
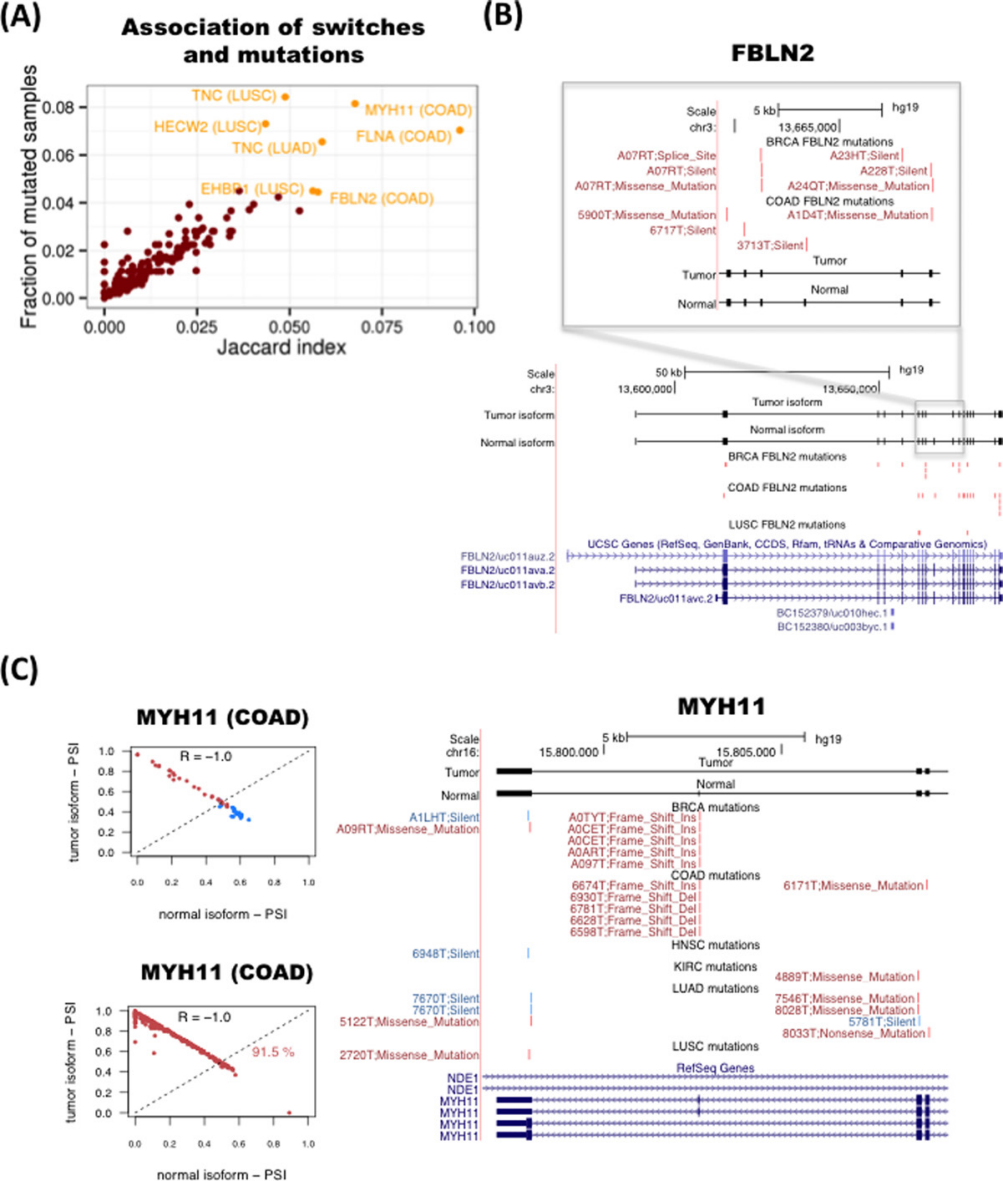
Association between somatic mutations and isoform switches. **(A)** Plot of the Jaccard index (*x*-axis) for the association of mutations with switches in tumor samples and the frequency of samples with mutations in the transcripts undergoing the switch (*y*-axis). **(B)** Example of the tumor suppressor FBLN2. Mutations present in each cancer type are represented in red if the switch is present in the same sample, and in blue if that sample does not have the switch. Each mutation is labeled with the identifier of the sample and the type of mutation. **(C)** Example of the oncogene MYH11. The relative inclusion values (PSI) of the two isoforms in the switch (left panels) separate tumor and normal in COAD and can classify correctly 91.5% of the unpaired tumor samples. Mutations present in each cancer type (right panel) are represented in red if the switch is present in the same sample, and in blue if that sample does not have the switch. Each mutation is labeled with the identifier of the sample and the type of mutation.

As splicing changes may be triggered by genetic mutations (12), we thus investigated whether any of the detected isoform switches may be caused by somatic mutations in the same genomic locus. To this end, we tested whether there was any association between the presence of the isoform switch in tumor samples and somatic mutations in the region of the transcript isoforms undergoing the switch in the same samples. Since in addition to intronic mutations, synonymous as well as non-synonymous mutations could alter the splicing of a gene (48), we considered all mutation types available in TCGA: coding-related (non-sense, missense, frameshift and indel) and non-coding-related (synonymous, splice-site and RNA) mutations. For each isoform-switch and for each cancer type, we calculated the Jaccard index across all samples for the association between the presence of the switch and the presence of somatic mutations (Figure 7A) (Materials and Methods). The Jaccard index agrees with the mutual information measure and do not correlate with the average mRNA length of the switches (Supplementary Figure S30). This analysis shows that FBLN2, MYH11, FLNA and TNC have the strongest association between mutations and switches (Figure 7A and Supplementary Figure S30). These four genes are also the ones with switches with the highest frequency of mutated samples (Figure 7A). For FBLN2, we found several mutations in BRCA and COAD samples on the alternative exon and the flanking constitutive exons (Figure 7B). However, the number of somatic mutations would not be enough to explain all the switches observed. We also found frequent mutations in the alternatively spliced region of the oncogene MYH11. In particular, we found recurrent deletions and insertions on the alternative exon in COAD and BRCA tumor samples that coincide with the presence of the switch (Figure 7C), which fall on a region of low conservation that is next to a putative binding site for the splicing factor SRSF1 (Supplementary Figure S31). For FLNA and TNC we did not see a pattern of recurrence of somatic mutations in the region where the splicing variability occurs. In any case, the number of found mutations cannot explain in general the frequency of the switches observed.

Somatic mutations could also affect the magnitude of the splicing change in specific samples. We therefore tested, for each isoform-switch, whether the presence of mutations in a tumor sample is associated with a larger difference of PSI between the pair of isoforms involved in the switch (Materials and Methods). Among the most significant cases, we found TGM1 and SLK, both in LUSC (Supplementary Figure S32). These two cases show differences in the distribution of PSI differences in samples with and without mutations (Supplementary Figure S32), suggesting that somatic mutations may be partly responsible for the differences in the relative abundance of the two transcripts involved in the switch in these genes. However, the proportion of mutated samples is very small to make a reliable comparison and after multiple-testing correction, none of the found cases remained significant. This suggests that, except for a limited number of cases, mutations may not be the main cause of the recurrent splicing switches found in tumors.

We thus hypothesized that mutations and isoform switches may occur independently as two alternative mechanisms of functional transformation in cancer. To test this possibility, we measured how frequently mutations that affect the protein-coding region occur in tumor samples without the isoform switch in the same gene by defining a mutual-exclusion score based on the number of samples with no switch but with protein-affecting mutations (Materials and Methods). We found that in general the mutual exclusion score correlates with the overall proportion of mutated samples (Supplementary Figure S33). However, the number of samples with both switch and mutation is generally comparable or higher than the number of samples with mutation and no switch, except for the genes TNC and HECW2 in LUSC, for which we find more samples with a protein-affecting mutation and no switch than with switch and protein-affecting mutation (Supplementary Figure S33). This suggests that both types of alterations, mutations and alternative splicing changes, could potentially contribute to cancer. We conclude that, although there are currently not a sufficient number of mutations that can provide clear patterns in relation to the described recurrent isoform switches, there are nonetheless a few cases for which this association may exist, as described for the genes FBLN2 and MYH11, and there is some evidence of mutual exclusion between protein-affecting mutations and alternative splicing, like for the genes TNC and HECW2.

## DISCUSSION

We have described a novel computational method to study consistent alternative splicing changes across multiple samples from two conditions to find predictive signatures with a potential functional impact in cancer. Classification rules are based on the relative expression of a single pair of isoforms per gene, which corresponds to an alternative splicing change between two conditions. Our method provides robust predictive models despite of the variability of transcript isoform expression across multiple samples, as the models are not dependent on parametrizations or on any normalization that would maintain the order in the ranking of isoform expression. This is especially useful for the analysis of RNA-Seq data from multiple samples, since between-sample normalization methods are not yet fully established. Moreover, our method can be used with data from heterogeneous platforms, as long as they provide a meaningful ranking of expression, which is convenient for the re-analysis of public data sets.

The application of our method to RNA-Seq data from the TCGA project has yielded classifiers that can distinguish tumor from normal samples, and between specific tumor subtypes, based on isoform changes. When tested on held-out data sets, the predictive models show overall high accuracies, which are comparable to the ones obtained using models based on gene expression patterns and involve different sets of genes; indicating that alternative splicing alterations describe independent cancer signatures, possibly due to cancer-specific splicing regulatory programs. Moreover, although individual isoform-pair rules do not show in general a strong predictive power, in combination they accurately classify tumor samples in the blind test. This suggests that splicing alterations are heterogeneous across samples, but in combination they provide characteristic signatures, similarly to the patterns of somatic mutations (5). This heterogeneity is further highlighted by the fact that different cancer types only share a small fraction of the found isoform changes. Although some of these changes may be explained by the differences in the cell composition of tumors (10,49), we observed a homogenous pattern of predicted tissue types in tumor and normal samples for most of the cancer types analyzed, indicating that the splicing changes are not a consequence of differences in cell type composition. Comparative analysis between cancer subtypes only yielded a significant model for basal-like breast tumors, which includes genes with known functional relation to cancer, indicating that most of the subtypes considered may share similar alternative splicing patterns.

Our analysis shows that isoform changes hold sufficient information to separate tumor and normal samples, and specific tumor subtypes, which suggests that they can serve as effective molecular markers, as they would only require measuring the expression of two isoforms per gene for a small number of genes. On the other hand, among all significant isoform changes, we found 244 isoform switches, for which the change occurs in the most abundant isoform, and are therefore more likely to have a functional impact. The predicted switches are validated using an independent computational method and are found to occur in genes from pathways frequently altered in tumors. Additionally, we find that the majority of the switches involve a change of the encoded protein or a change between a protein coding and a non-coding isoform, which suggests that they may have a functional impact. In particular, we predict isoform switches that affect the encoded protein for the cancer drivers CCND3, MYH11, MITF, RALGD5, ABI1, PRDM1 and PPARG, which may have implications for targeted therapy development. Interestingly, the predicted switches not only separate accurately tumor and normal samples, but they also group tumor samples according to cancer type. Thus, although multiple alternative splicing alterations occur in all cancer types, there seems to be distinctive regulatory programs that contribute to cancer-specific phenotypes. The isoform switches provide thus an opportunity to develop experimental strategies based on the detection of tumor-specific protein isoforms. For instance, QKI has a splicing switch in lung adenocarcinoma that cannot be described in terms of simple events and which has better predictive power than well-known splicing changes in other genes, like NUMB. Similarly, we found switches in genes involved in cell communication pathways, including DST and FLNA, which could be used for developing tumor-specific molecular targets with reduced cross-reactivity to other proteins.

Our analyses indicate that somatic mutations occurring on exons and splice-sites cannot explain in general the isoform switching patterns. In particular, 99% of the transcripts analyzed appear mutated in less than 5% of the tumor samples, whereas the switches occur in at least 50% of the samples. Despite the lack of somatic mutations, we found a significant association for two cases: the tumor suppressor FBLN2 and the cancer driver MYH11. Although it has been suggested that synonymous mutations in known cancer drivers may contribute to the oncogenic process (50), these occur at low frequency and a direct link between the observed mutations and specific splicing changes in the same tumor samples was not provided. The observed variation could still be due to intronic mutations not represented in the currently available exome sequencing data. Alternatively, the switches could be explained by alterations in splicing factors. Although point mutations and indels on splicing factors also occur at low frequency (14), splicing factors show frequent amplifications, deletions and expression changes in tumors (13). Another possibility is that alterations in chromatin modifications and DNA methylation are responsible for the observed changes. These alterations are frequent in cancers (51,52) and they may induce changes in splicing (53,54). Interestingly, FBLN2, which presents a switch in various cancers, has been observed frequently methylated in breast and other epithelial tumors (55). Further analysis of the frequency of mutations and switches shows that the gene TNC, linked to cell invasion in tumors (56), has a pattern of mutual exclusion between the isoform switch and the somatic mutations affecting the coding regions, suggesting that, albeit to a limited extent, splicing switches may provide an alternative mechanism toward functional transformation in cancer.

In conclusion, we have derived accurate predictive models based on transcript isoform changes from multiple patient samples and recurrent isoform switches with potential application in molecular prognosis and for the exploration of novel therapeutic strategies. Our analysis of nine cancer types indicate that recurrent changes in splicing may contribute together with mutations and other alterations to explain tumor formation, thereby providing novel signatures for cancer.

## SUPPLEMENTARY DATA

Supplementary Data are available at NAR Online.

SUPPLEMENTARY DATA
