# Supplementary material for: Detection of recurrent alternative splicing switches in tumor samples reveals novel signatures of cancer
Source: Nucleic Acids Res. 2015 Jan 10;43(3):1345–56. doi: 10.1093/nar/gku1392 (PMC4330360; doi:10.1093/nar/gku1392)
Supplement: SUPPLEMENTARY DATA [file supp_43_3_1345__index.html]

Detection of recurrent alternative splicing switches in tumor samples reveals novel signatures of cancer — Detection of recurrent alternative splicing switches in tumor samples reveals novel signatures of cancer — SUPPLEMENTARY DATA 

# Detection of recurrent alternative splicing switches in tumor samples reveals novel signatures of cancer

## SUPPLEMENTARY DATA

**Files in this Data Supplement:**

- SUPPLEMENTARY DATA
- SUPPLEMENTARY DATA
- SUPPLEMENTARY DATA
- SUPPLEMENTARY DATA
- SUPPLEMENTARY DATA
