# Supplementary material for: Detection of recurrent alternative splicing switches in tumor samples reveals novel signatures of cancer
Source: Nucleic Acids Res. 2015 Jan 10;43(3):1345–56. doi: 10.1093/nar/gku1392 (PMC4330360; doi:10.1093/nar/gku1392)
Supplement: SUPPLEMENTARY DATA [file supp_gku1392_nar-02177-met-g-2014-File009.pdf]

Supplementary Material

Detection of recurrent alternative splicing switches in tumor samples reveals novel signatures of cancer

Endre Sebestyén[1], Michał Zawisza[2], Eduardo Eyras[1,3,4]

[1]Computational Genomics, Universitat Pompeu Fabra, Dr. Aiguader 88, E08003 Barcelona, Spain
[2]Universitat Politècnica de Catalunya, Jordi Girona 1-3, Barcelona E08034, Spain
[3]Catalan Institution for Research and Advanced Studies, Passeig Lluís Companys 23, E08010 Barcelona, Spain
[4]Correspondence to: eduardo.eyras@upf.edu

(A) **BRCA**

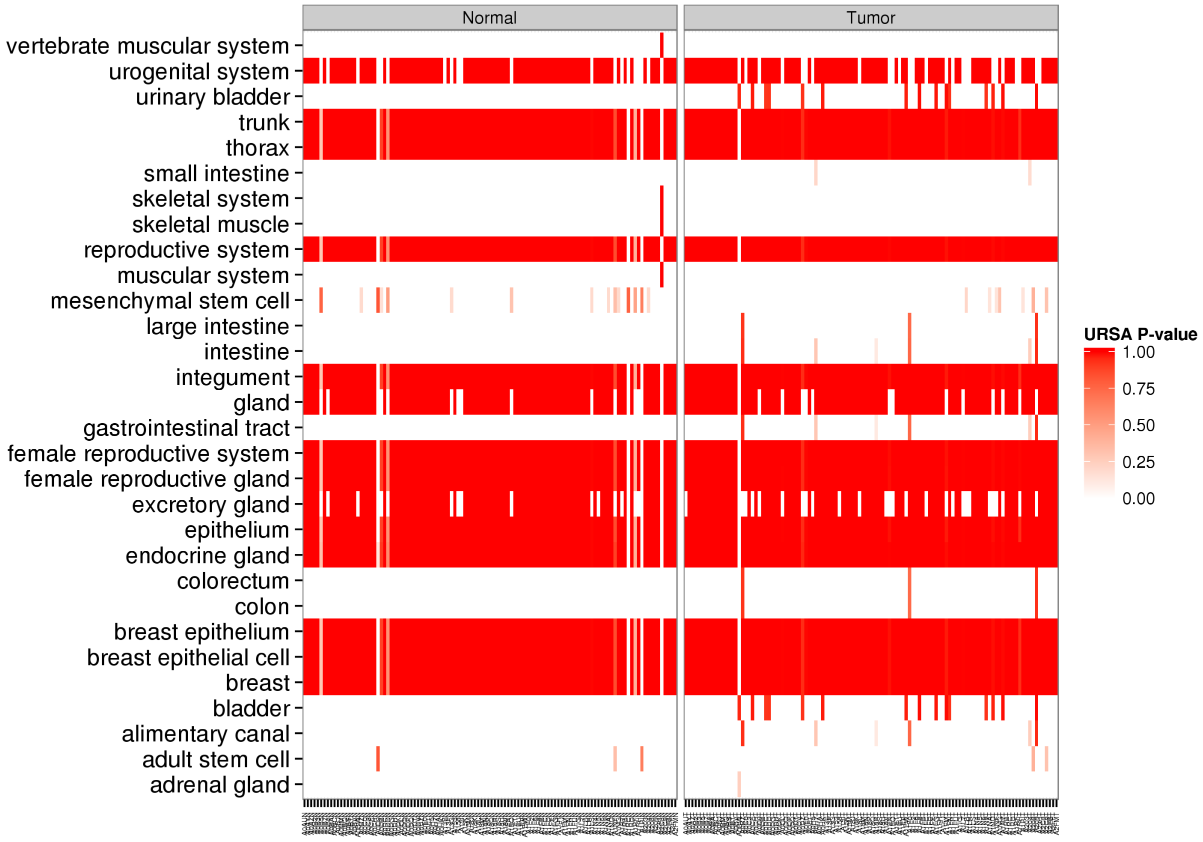

Supplementary Figure 1. For each cancer type, we run URSA (Lee et al. 2013) on the estimated read-counts per gene for all the paired-samples and kept only the tissue predictions with posterior probability P>0.1 (y axis). Tumor and normal samples (x axis) were then clustered separately. Both paired-samples from the same patient were removed if either of the two, tumor or normal, did not cluster with the rest of the same type. The heatmaps show the full original sample list for each cancer type: BRCA **(A)**, COAD **(B)**, HNSC **(C)**, KICH **(D)**, KIRC **(E)**, LUAD **(F)**, LUSC **(G)**, PRAD **(H)** and THCA **(I)**.

(B)

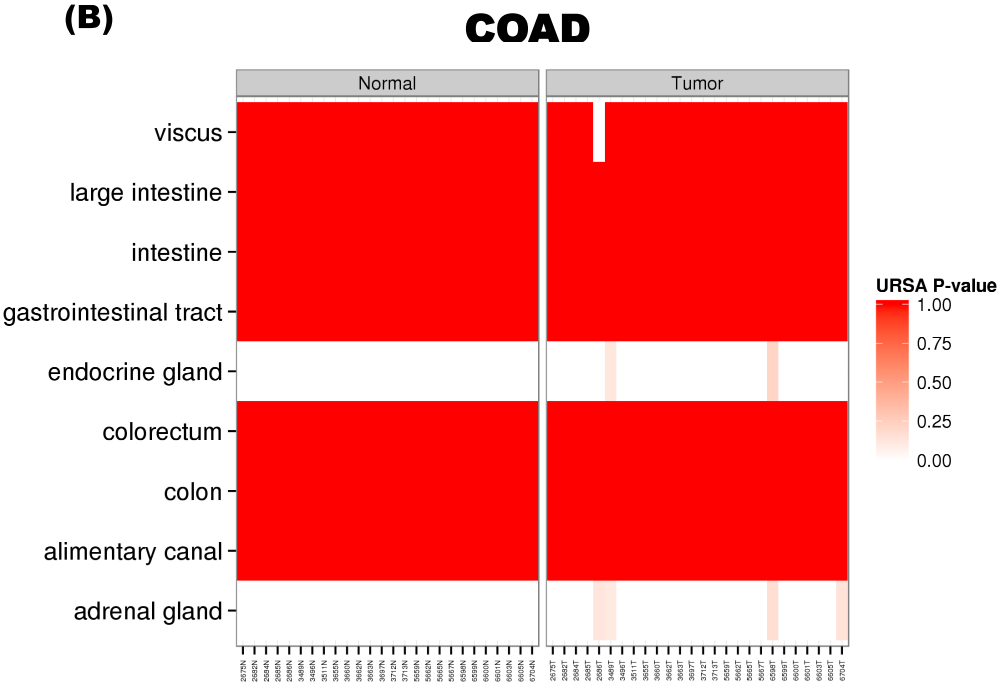
COAD
Normal
Tumor
viscus
large intestine
intestine
gastrointestinal tract
endocrine gland
colorectum
colon
alimentary canal
adrenal gland
URSA P-value
1.00
0.75
0.50
0.25
0.00

(C)

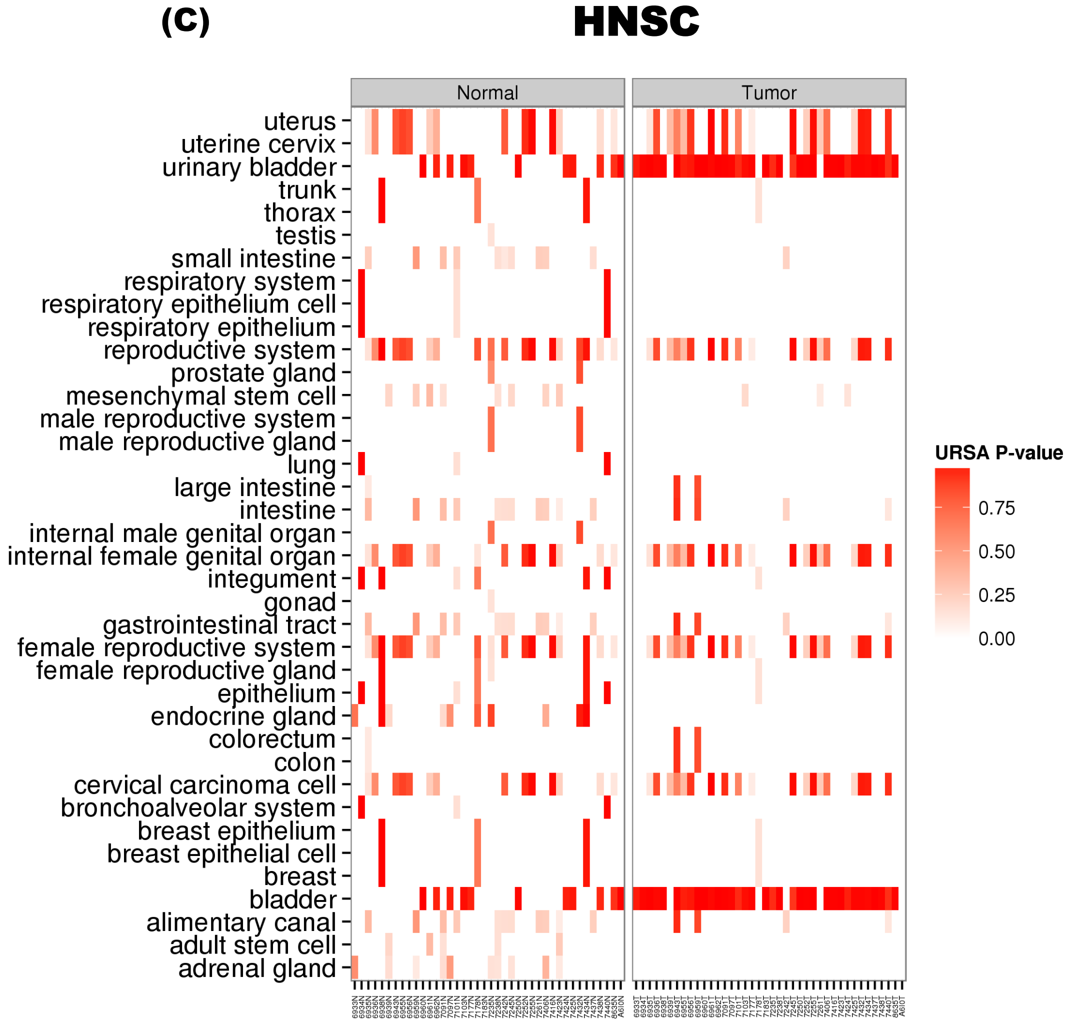
HNSC
Normal
Tumor
uterus
uterine cervix
urinary bladder
trunk
thorax
testis
small intestine
respiratory system
respiratory epithelium cell
respiratory epithelium
reproductive system
prostate gland
mesenchymal stem cell
male reproductive system
male reproductive gland
lung
large intestine
intestine
internal male genital organ
internal female genital organ
integument
gonad
gastrointestinal tract
female reproductive system
female reproductive gland
epithelium
endocrine gland
colorectum
colon
cervical carcinoma cell
bronchoalveolar system
breast epithelium
breast epithelial cell
breast
bladder
alimentary canal
adult stem cell
adrenal gland
URSA P-value
0.75
0.50
0.25
0.00

KICH

(D)

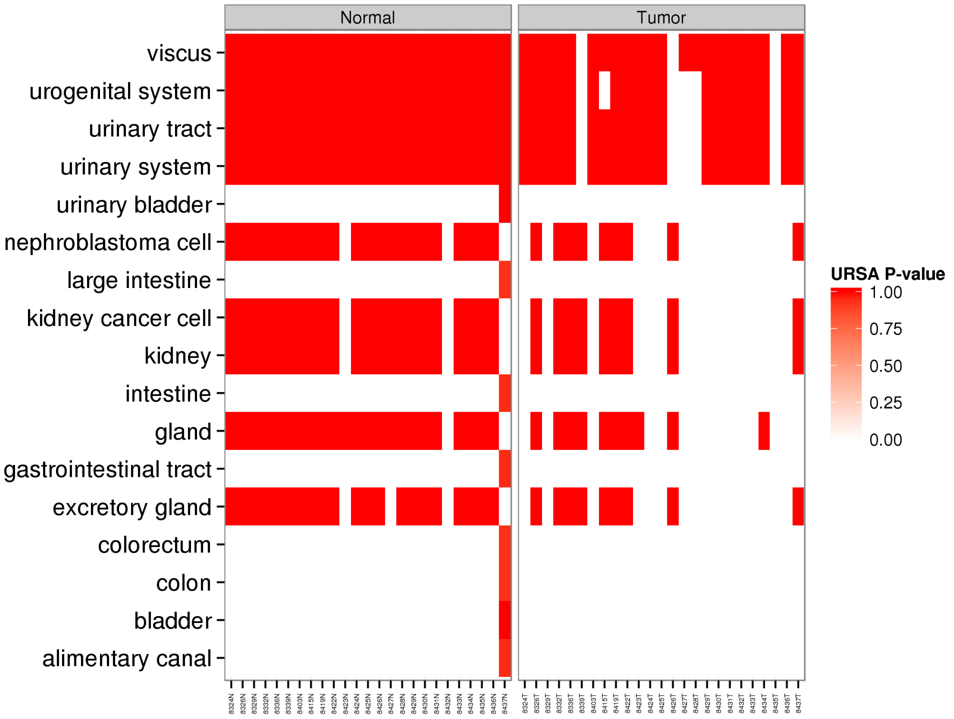
Normal
Tumor
viscus
urogenital system
urinary tract
urinary system
urinary bladder
nephroblastoma cell
large intestine
kidney cancer cell
kidney
intestine
gland
gastrointestinal tract
excretory gland
colorectum
colon
bladder
alimentary canal
URSA P-value
1.00
0.75
0.50
0.25
0.00

(E)

KIRC

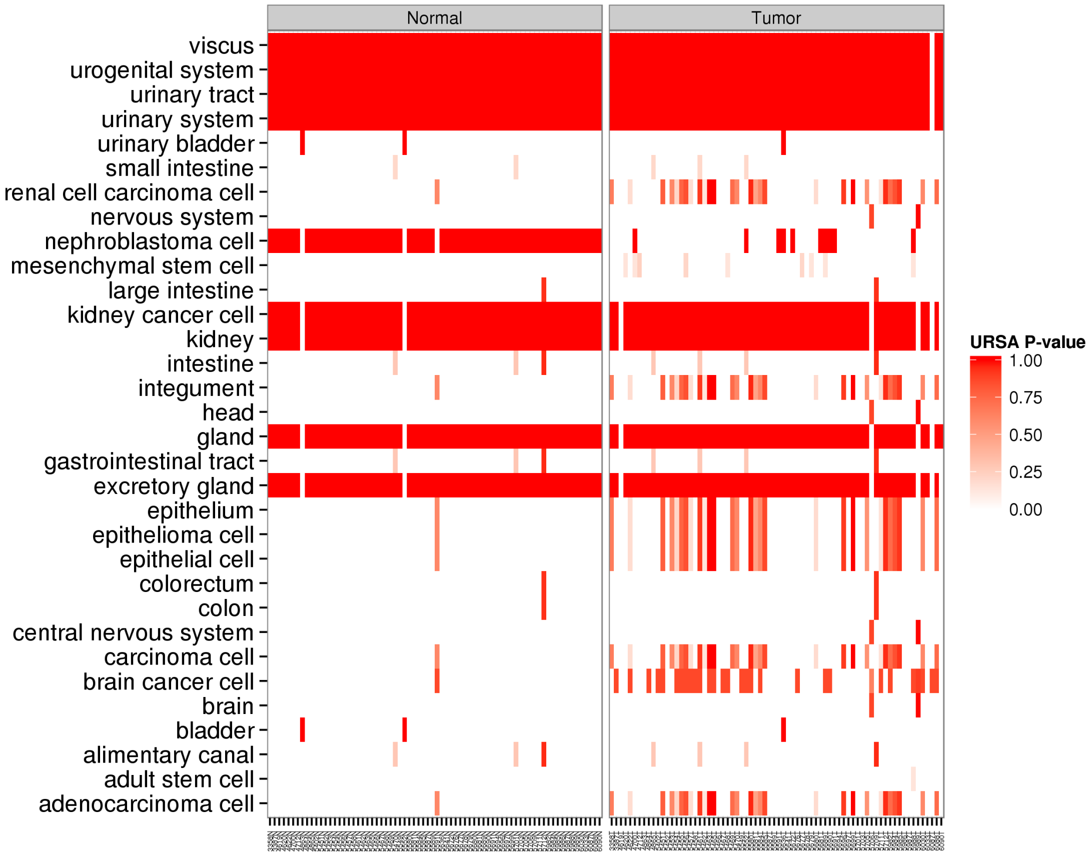
Normal
Tumor
viscus
urogenital system
urinary tract
urinary system
urinary bladder
small intestine
renal cell carcinoma cell
nervous system
nephroblastoma cell
mesenchymal stem cell
large intestine
kidney cancer cell
kidney
intestine
integument
head
gland
gastrointestinal tract
excretory gland
epithelium
epithelioma cell
epithelial cell
colorectum
colon
central nervous system
carcinoma cell
brain cancer cell
brain
bladder
alimentary canal
adult stem cell
adenocarcinoma cell
URSA P-value
1.00
0.75
0.50
0.25
0.00

(F)

LUAD

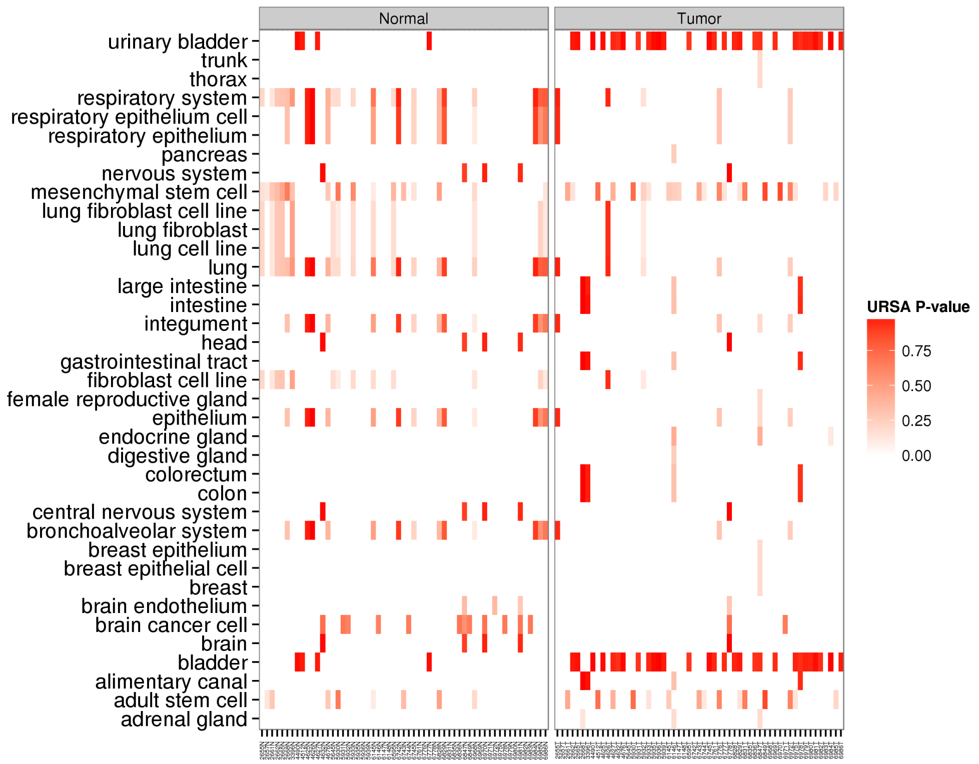
Normal
Tumor
urinary bladder
trunk
thorax
respiratory system
respiratory epithelium cell
respiratory epithelium
pancreas
nervous system
mesenchymal stem cell
lung fibroblast cell line
lung fibroblast
lung cell line
lung
large intestine
intestine
integument
head
gastrointestinal tract
fibroblast cell line
female reproductive gland
epithelium
endocrine gland
digestive gland
colorectum
colon
central nervous system
bronchoalveolar system
breast epithelium
breast epithelial cell
breast
brain endothelium
brain cancer cell
brain
bladder
alimentary canal
adult stem cell
adrenal gland
URSA P-value
0.75
0.50
0.25
0.00

(G)

LUSC

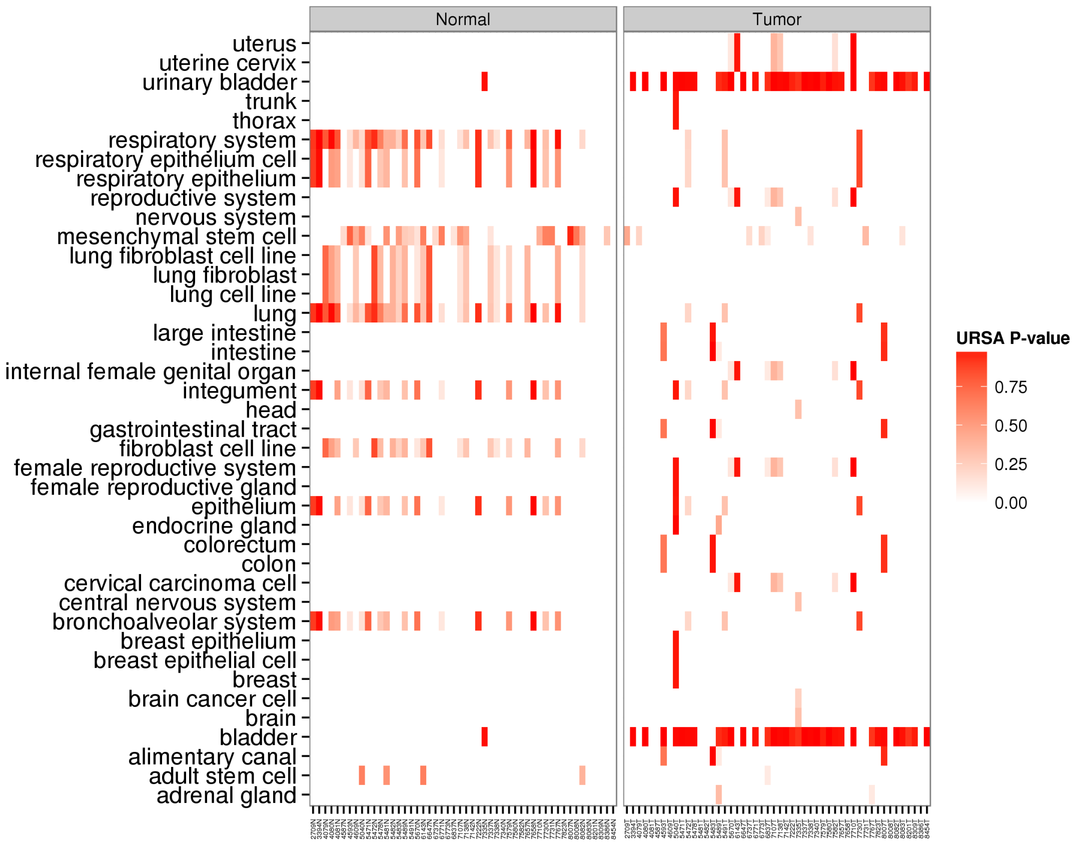
Normal
Tumor
uterus
uterine cervix
urinary bladder
trunk
thorax
respiratory system
respiratory epithelium cell
respiratory epithelium
reproductive system
nervous system
mesenchymal stem cell
lung fibroblast cell line
lung fibroblast
lung cell line
lung
large intestine
intestine
internal female genital organ
integument
head
gastrointestinal tract
fibroblast cell line
female reproductive system
female reproductive gland
epithelium
endocrine gland
colorectum
colon
cervical carcinoma cell
central nervous system
bronchoalveolar system
breast epithelium
breast epithelial cell
breast
brain cancer cell
brain
bladder
alimentary canal
adult stem cell
adrenal gland
URSA P-value
0.75
0.50
0.25
0.00

(H) **PRAD**

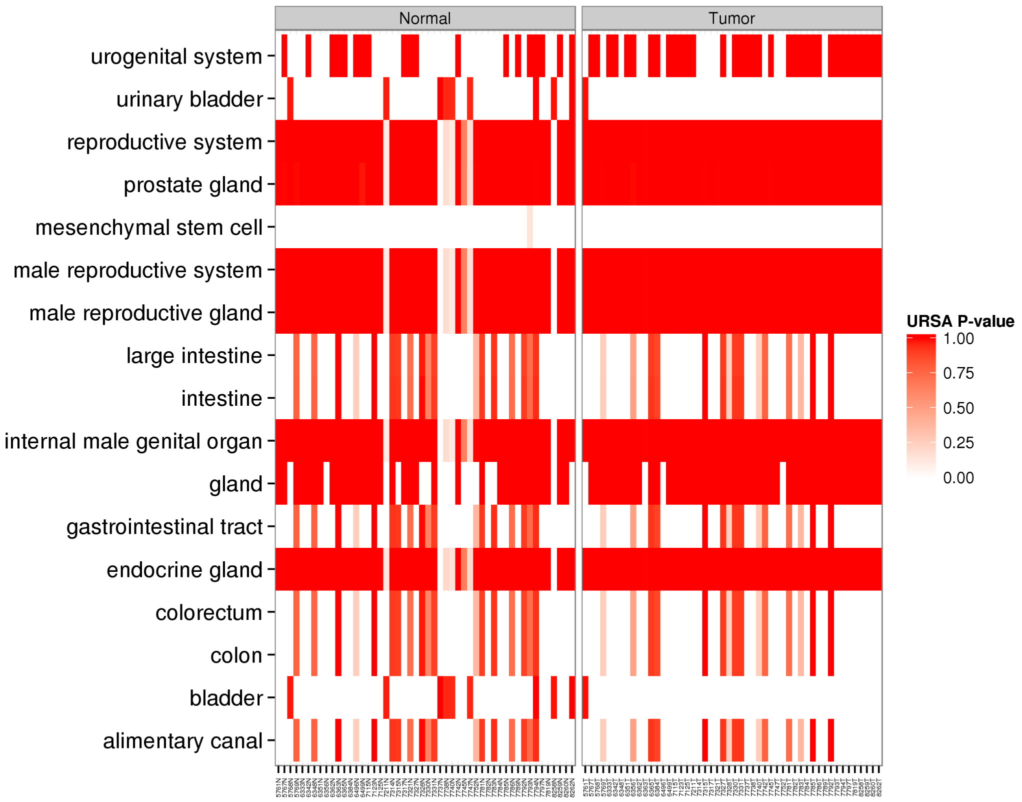

Normal
Tumor
urogenital system
urinary bladder
reproductive system
prostate gland
mesenchymal stem cell
male reproductive system
male reproductive gland
large intestine
intestine
internal male genital organ
gland
gastrointestinal tract
endocrine gland
colorectum
colon
bladder
alimentary canal
URSA P-value
1.00
0.75
0.50
0.25
0.00

(I) **THCA**

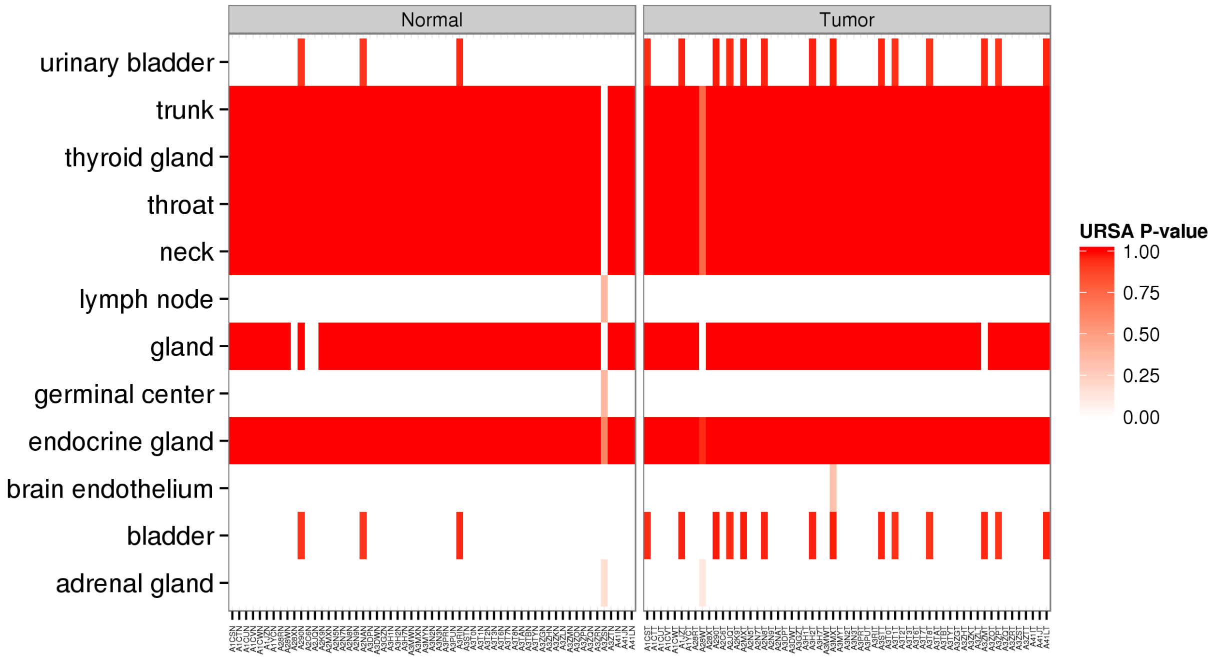

Normal
Tumor
urinary bladder
trunk
thyroid gland
throat
neck
lymph node
gland
germinal center
endocrine gland
brain endothelium
bladder
adrenal gland
URSA P-value
1.00
0.75
0.50
0.25
0.00

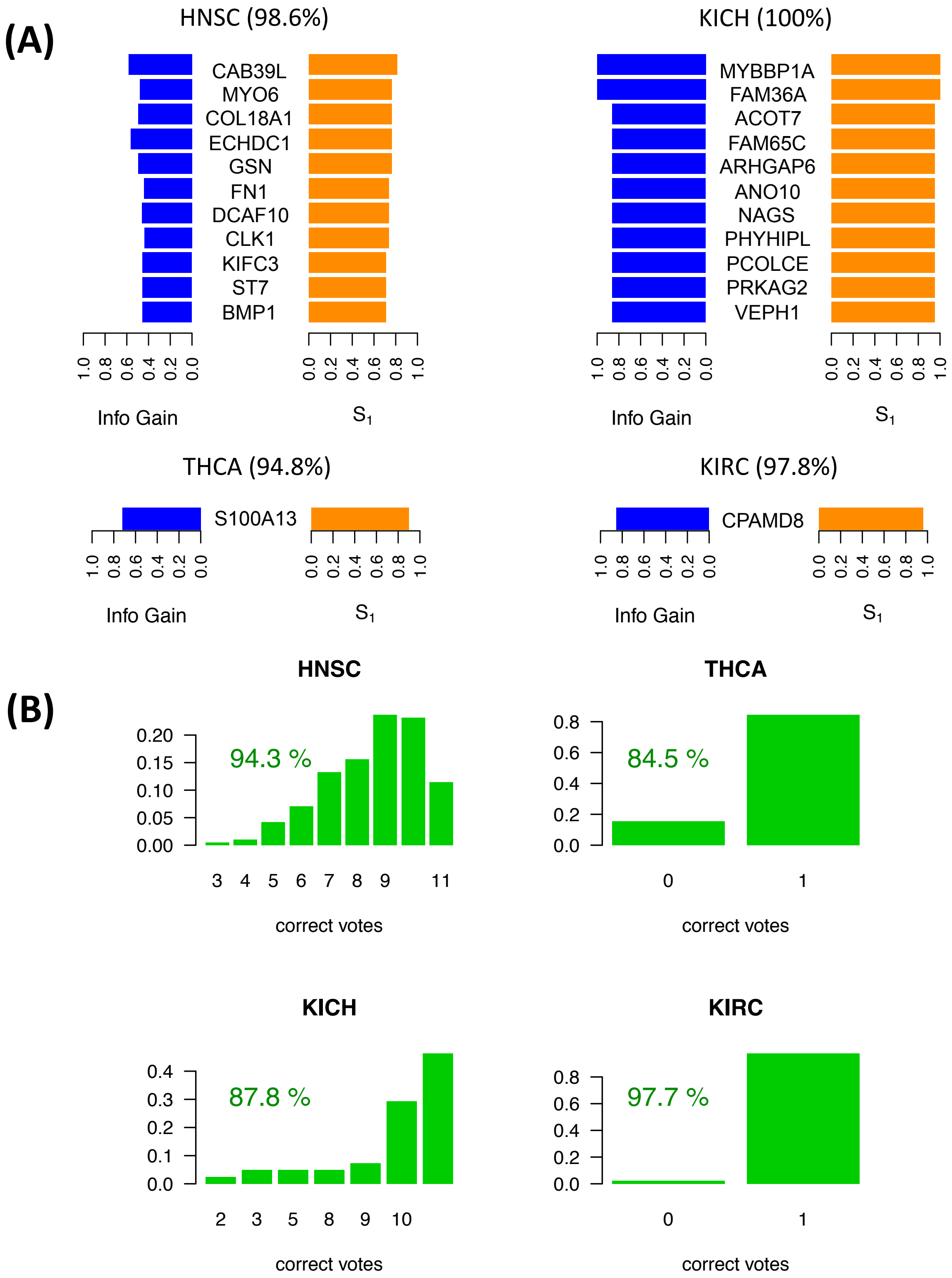

Supplementary Figure 2. **(A)** Isoform-pair models for HNSC, KICH, THCA and KIRC. Each panel shows the score S_1 and information gain (IG) for the selected models in each cancer type. All isoform pairs in the models are significant according to permutation analyses. Next to each cancer label, the expected average accuracy from the cross-validation analysis is given. **(B)** Test of the derived models on the set of held-out samples for the same cancer type. The tests were carried out on the unpaired tumor samples from Table 1. Barplots indicate the proportion of samples (y-axis) for each number of possible correct votes (x-axis), i.e., the number of isoform-pair rules from the model. A sample is labelled according to majority vote, i.e. it is correctly classified if there more correct than incorrect isoform-pair rules.

.

(A)

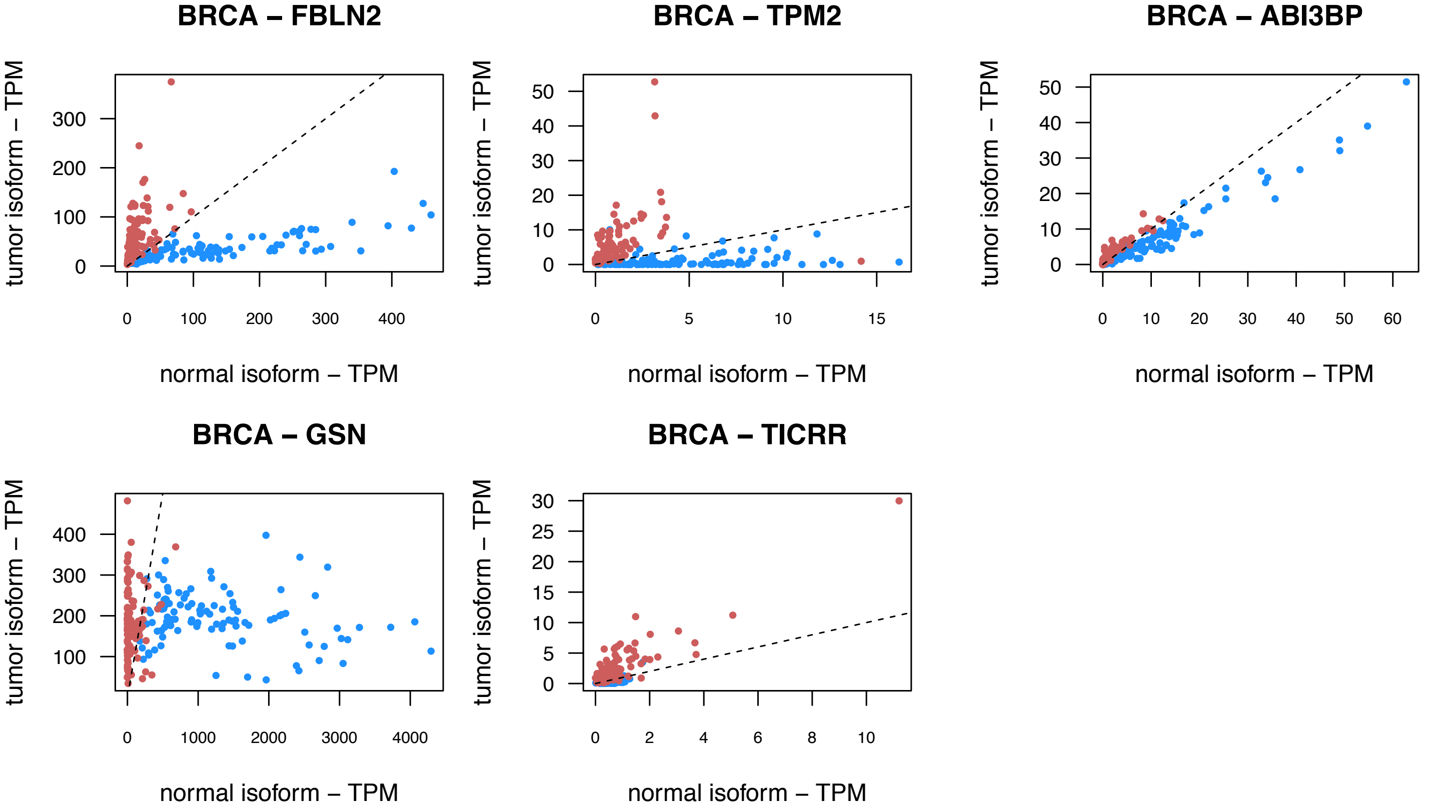

(B)

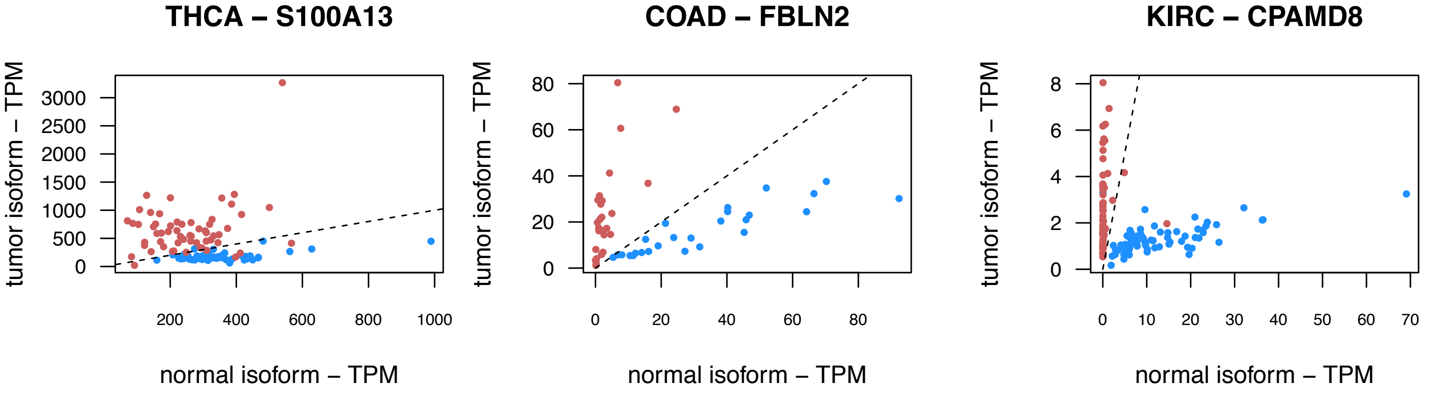

Supplementary Figure 3. Transcript expression plots, also called top scoring pair plots (Geman et al. 2004), for the isoform-pairs of the BRCA model **(A)** and the single-gene models of THCA, COAD and KIRC **(B)**. The plots show the expression for one isoform in the pair versus the expression for the second isoform in the pair for tumor (red) and normal (blue) samples. Expression values are given in transcript per million (TPM) units. The dashed line indicates the equal expression and represents the classification rule. The accuracy of the model is determined by how well the line separates the two samples.

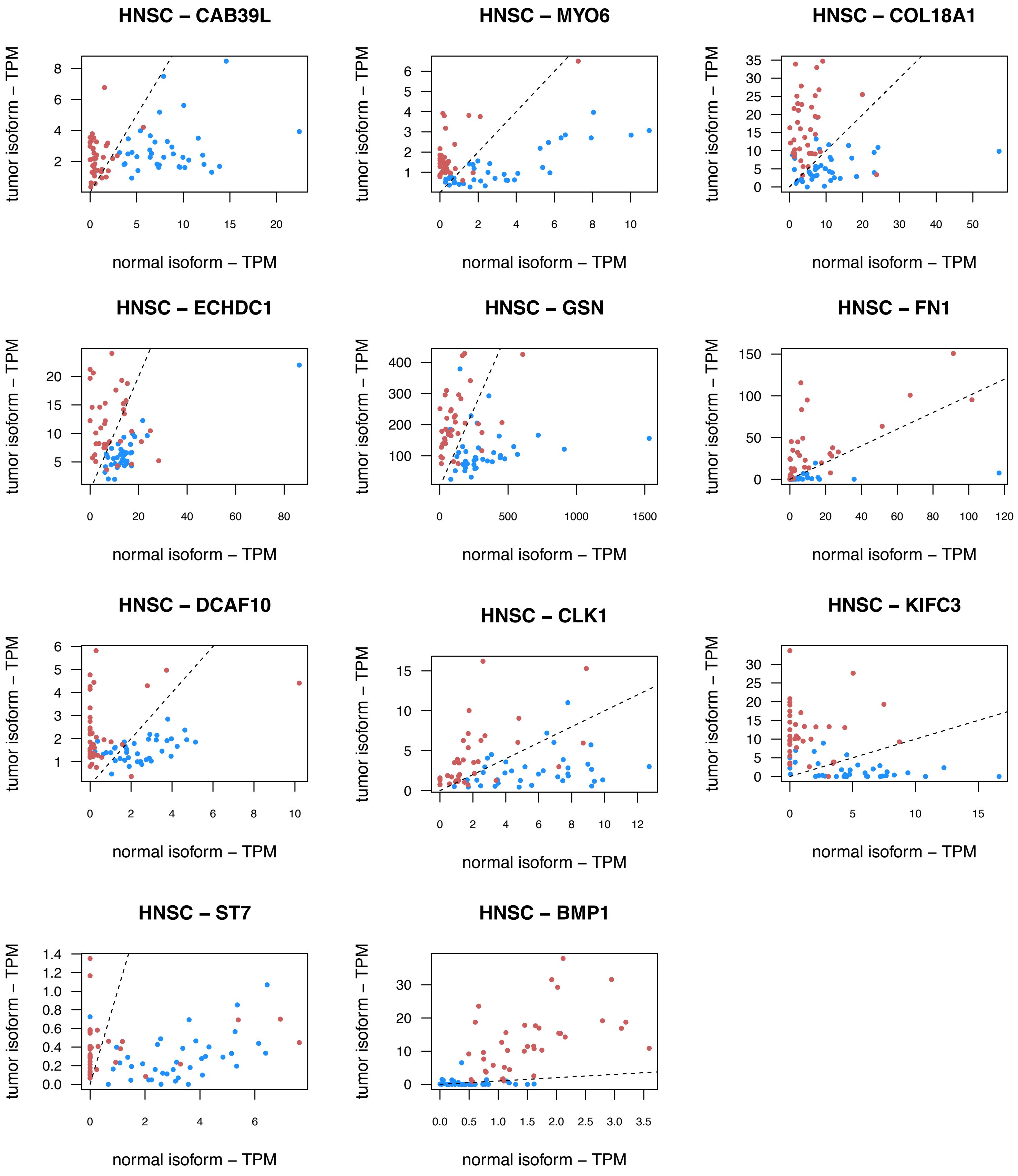

Supplementary Figure 4. Transcript expression plots for the isoform-pairs of the HNSC model The plots show the expression for one isoform in the pair versus the expression for the second isoform in the pair for tumor (red) and normal (blue) samples. Expression values are given in transcript per million (TPM) units. The dashed line indicates the equal expression and represents the classification rule. The accuracy of the model is determined by how well the line separates the two samples.

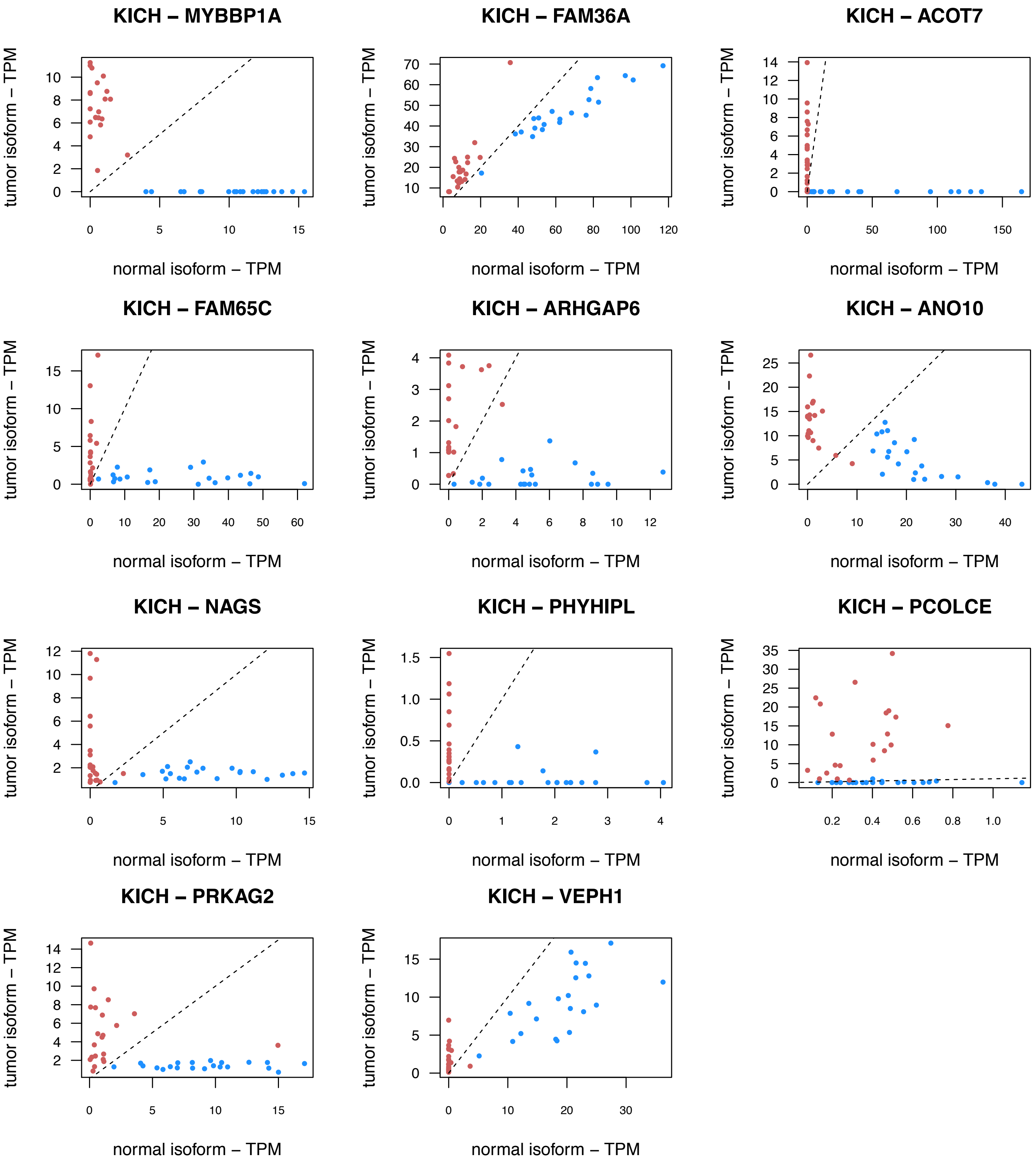

Supplementary Figure 5. Transcript expression plots for the isoform-pairs of the KICH model The plots show the expression for one isoform in the pair versus the expression for the second isoform in the pair for tumor (red) and normal (blue) samples. Expression values are given in transcript per million (TPM) units. The dashed line indicates the equal expression and represents the classification rule. The accuracy of the model is determined by how well the line separates the two samples.

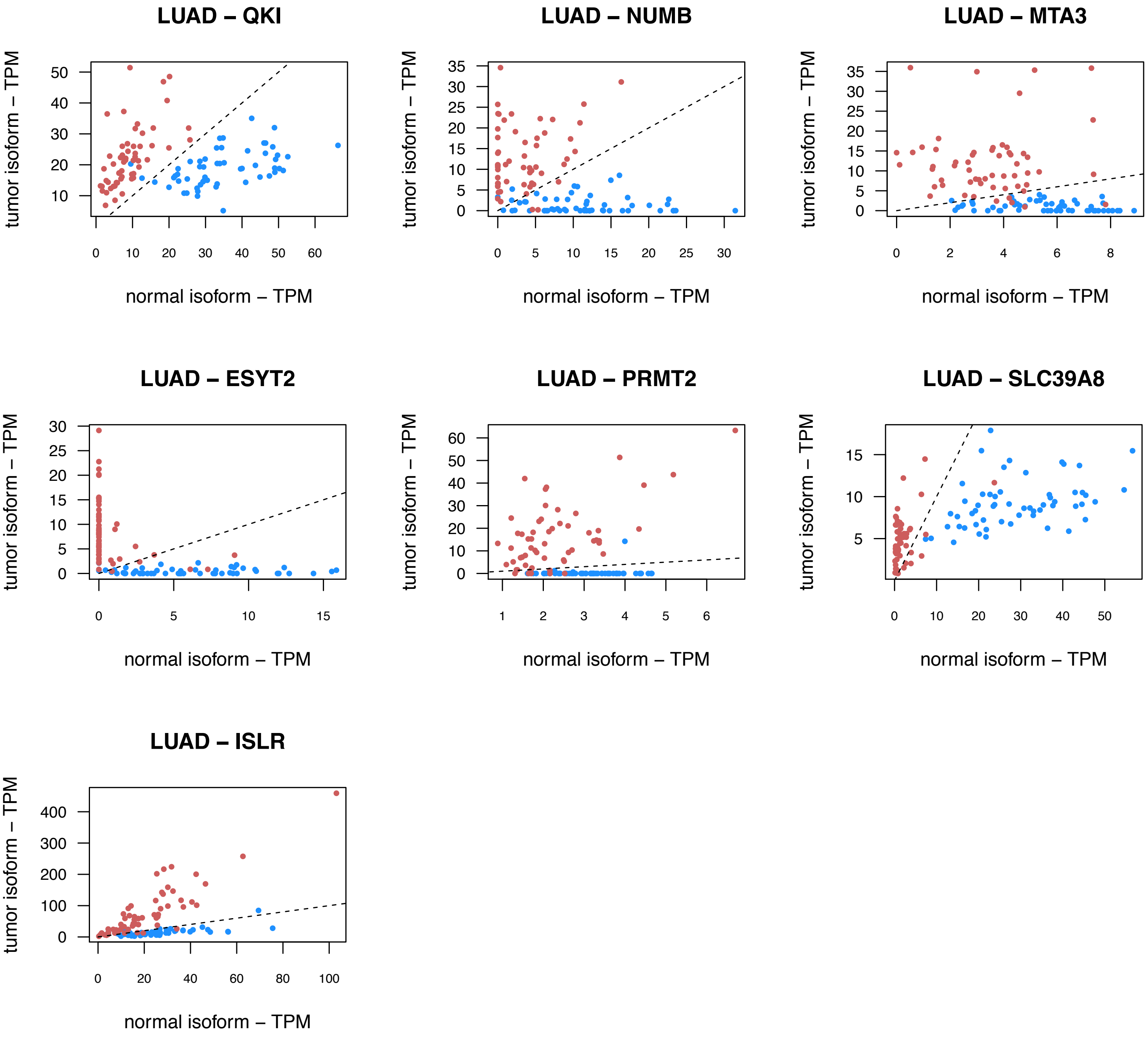

Supplementary Figure 6. Transcript expression plots for the isoform-pairs of the LUAD model. The plots show the expression for one isoform in the pair versus the expression for the second isoform in the pair for tumor (red) and normal (blue) samples. Expression values are given in transcript per million (TPM) units. The dashed line indicates the equal expression and represents the classification rule. The accuracy of the model is determined by how well the line separates the two samples.

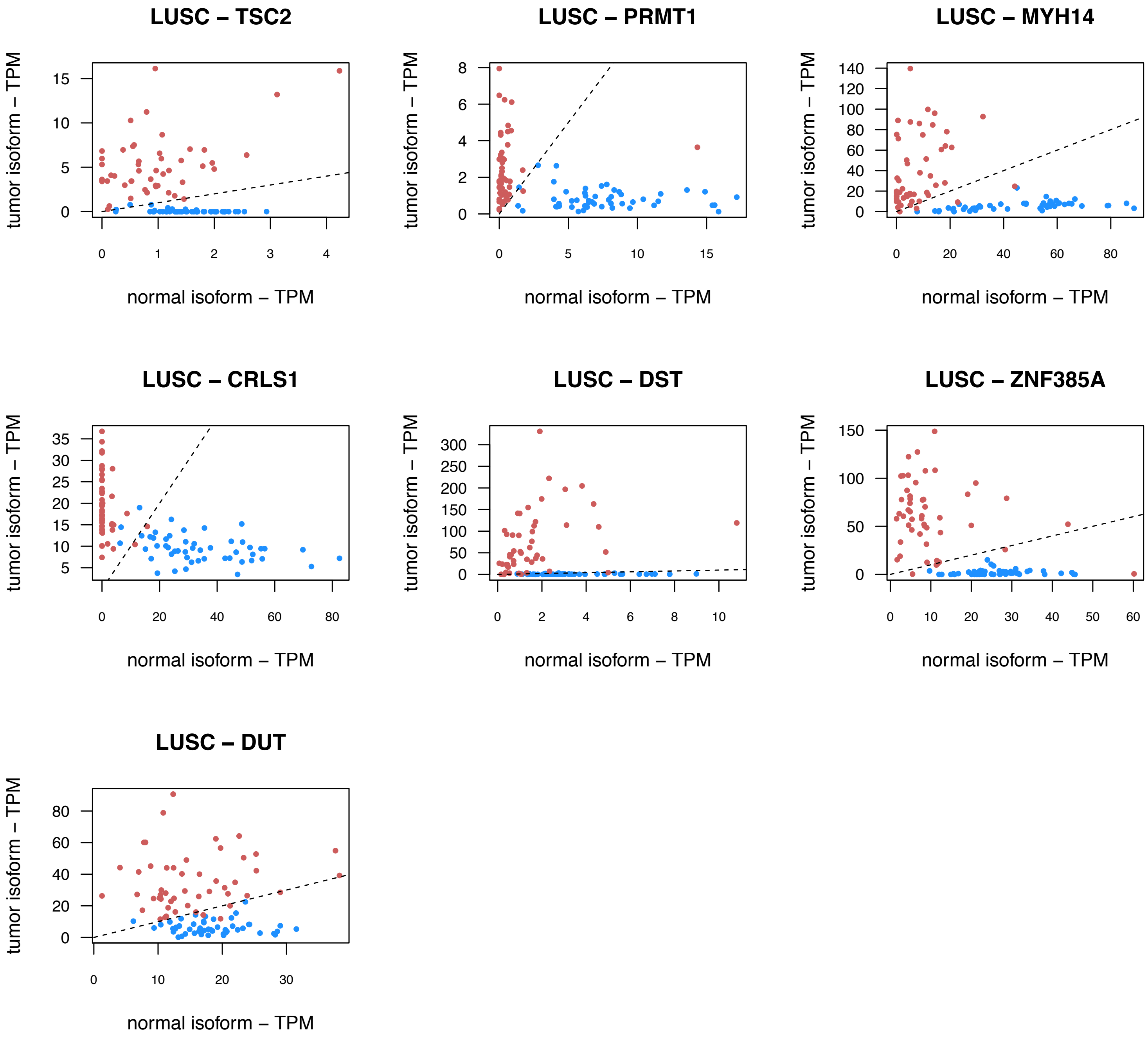

Supplementary Figure 7. Transcript expression plots for the isoform-pairs of the LUSC model. The plots show the expression for one isoform in the pair versus the expression for the second isoform in the pair for tumor (red) and normal (blue) samples. Expression values are given in transcript per million (TPM) units. The dashed line indicates the equal expression and represents the classification rule. The accuracy of the model is determined by how well the line separates the two samples.

PRAD

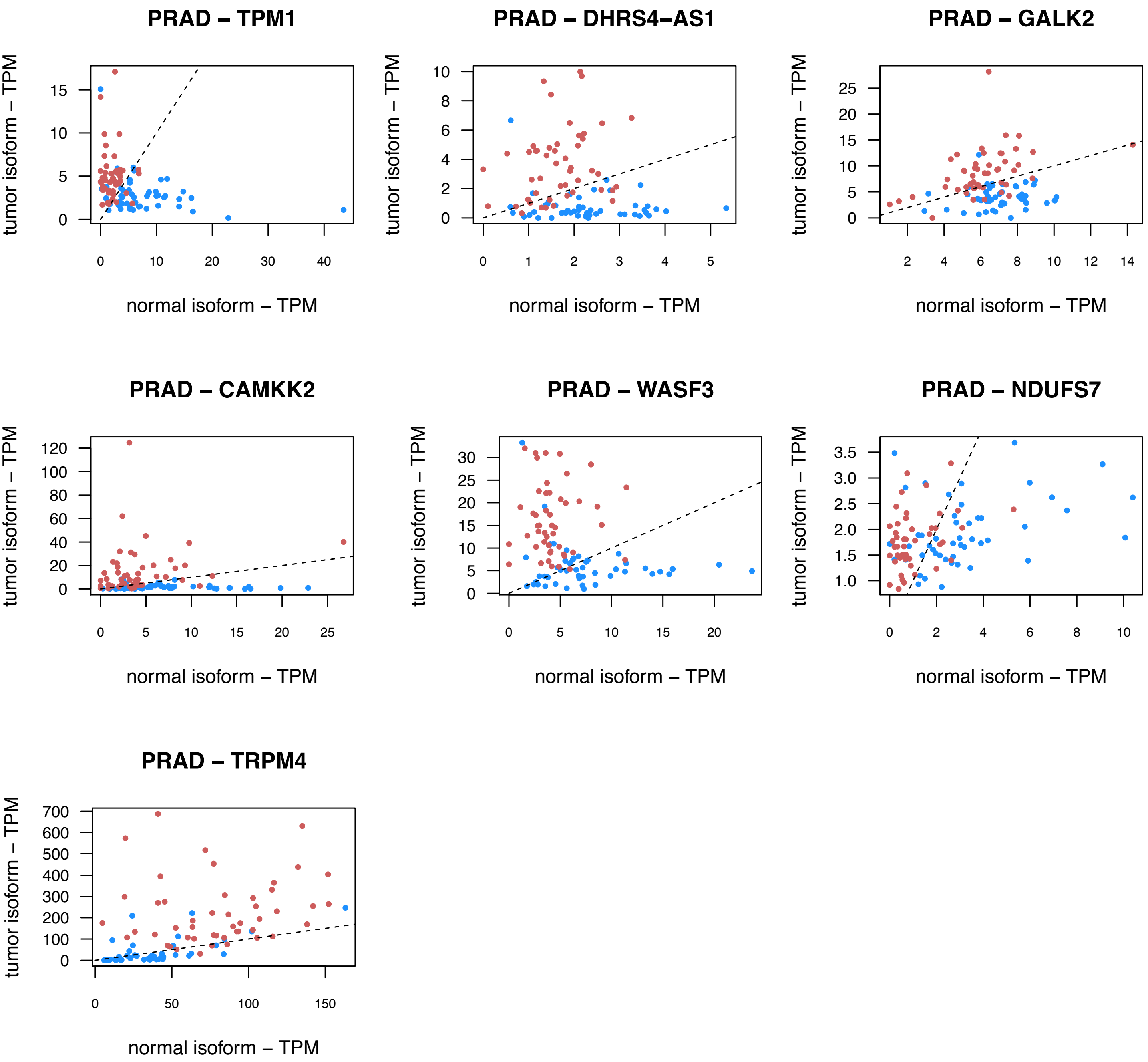

Supplementary Figure 8. Transcript expression plots for the isoform-pairs of the PRAD model. The plots show the expression for one isoform in the pair versus the expression for the second isoform in the pair for tumor (red) and normal (blue) samples. Expression values are given in transcript per million (TPM) units. The dashed line indicates the equal expression and represents the classification rule. The accuracy of the model is determined by how well the line separates the two samples. DHRS4-AS1 (C14orf167) is antisense of DHRS4

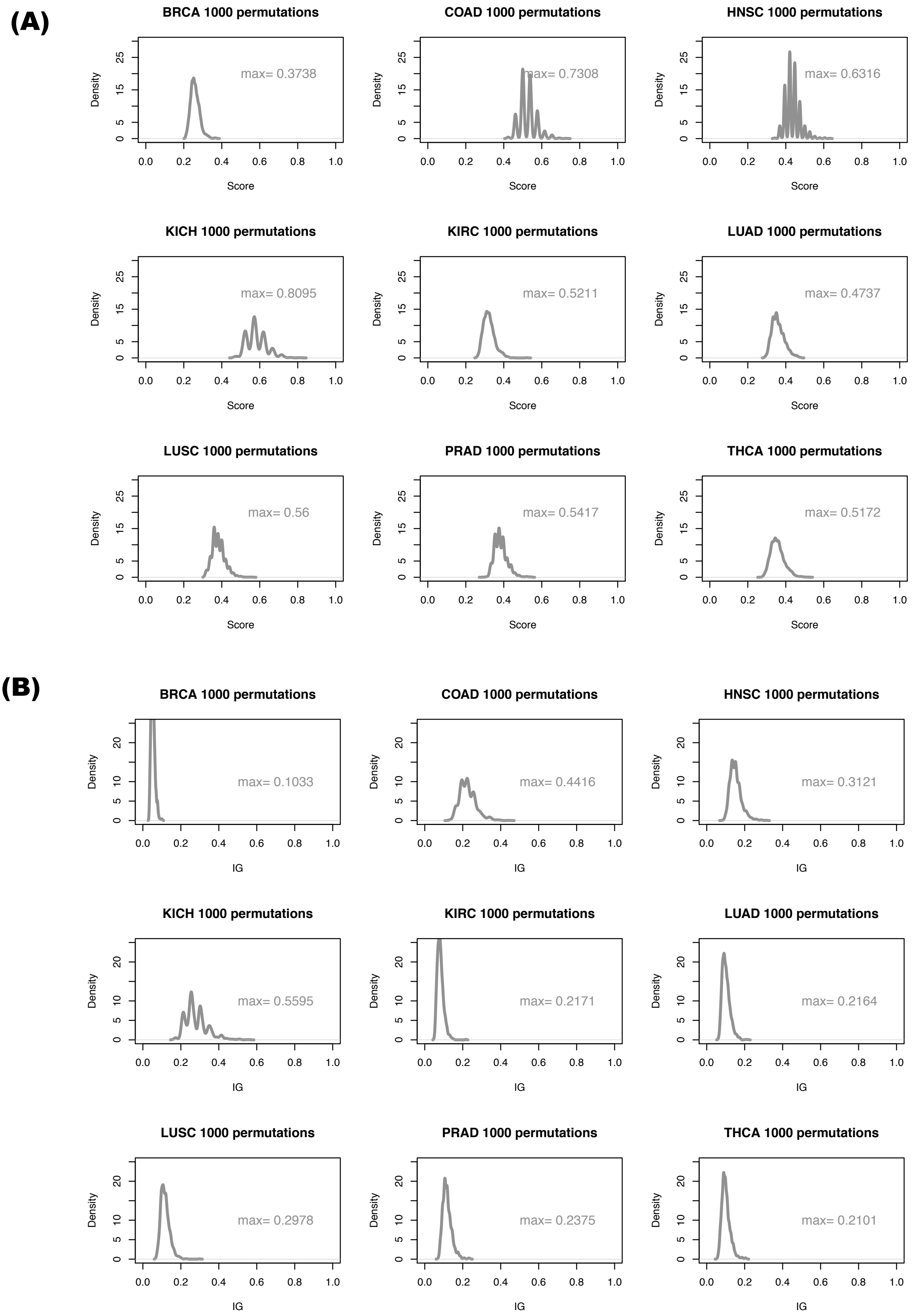

Supplementary Figure 9. Permutation analysis of the paired samples for the 9 cancer types. 1000 permutations of the labels were carried out, and at each permutation the Iso-kTSP algorithm was run and the top isoform-pair according to the cross-validation was selected. The plots show the distributions for the score S_1 (Score) **(A)** and Information Gain (IG) **(B)** for these 1000 top isoform-pairs of the permuted labels. An observed isoform-pair is considered significant if its individual score S_1 and IG values are larger than the maximum ones obtained from the 1000 permutations.

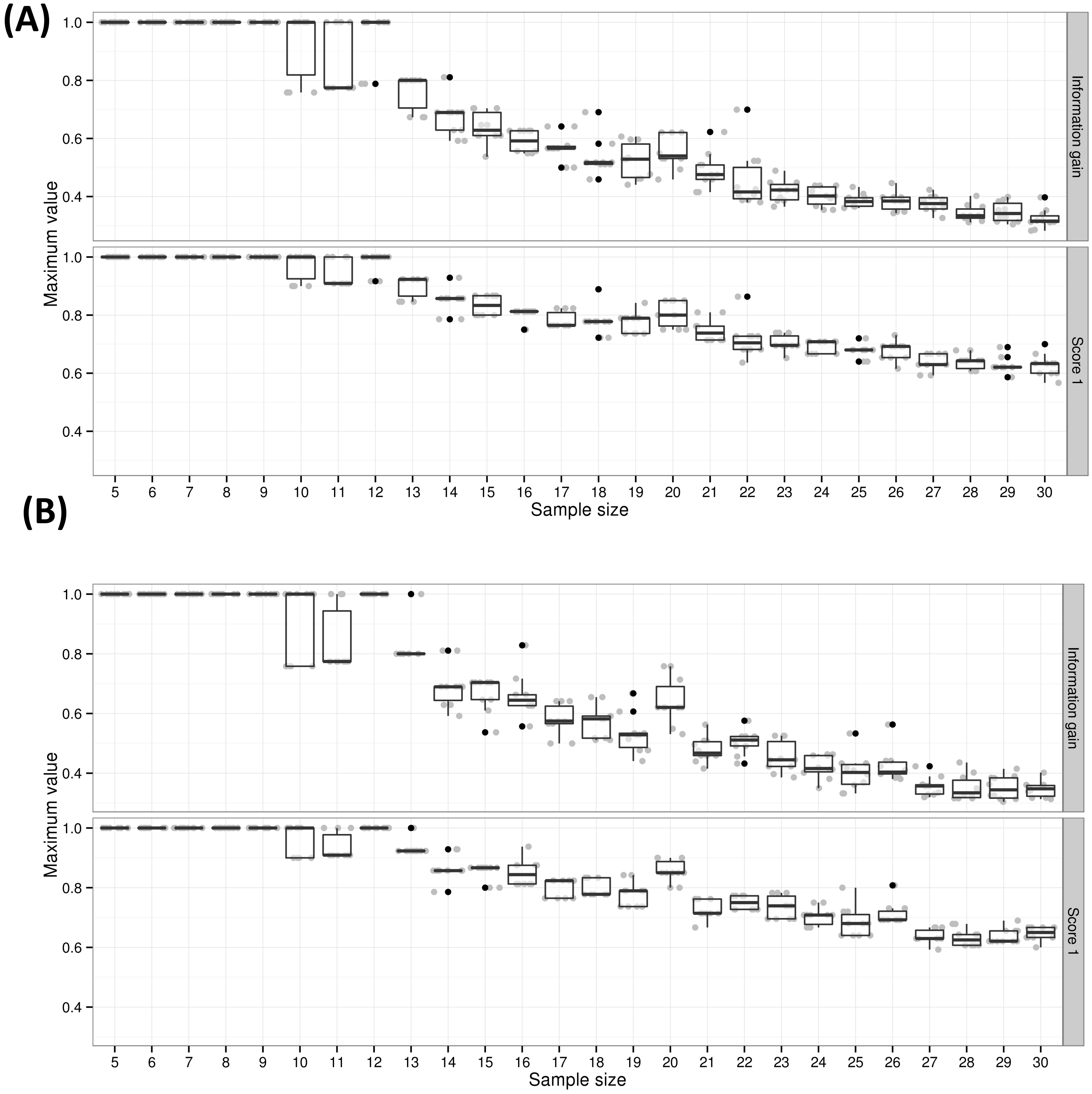

Supplementary Figure 10. Sample permutation with variable number of input samples. For BRCA **(A)** and LUSC **(B)**, we plot the distributions of the maximum values of the score S_1 and Information Gain for 10 permutations, using as input 5,6,… 30 paired input samples (x-axis). The plots indicate that below 12 paired samples (12 tumor and 12 normal), permuting the labels can produce with probability close to 1 isoform changes with score S_1 and IG close to 1. However, with 14 or more labels (13 for BRCA), permutation of the labels produces isoform changes by chance with smaller score 1 and IG values. Thus, significant isoform changes can be produced robustly with 14 or more paired samples.

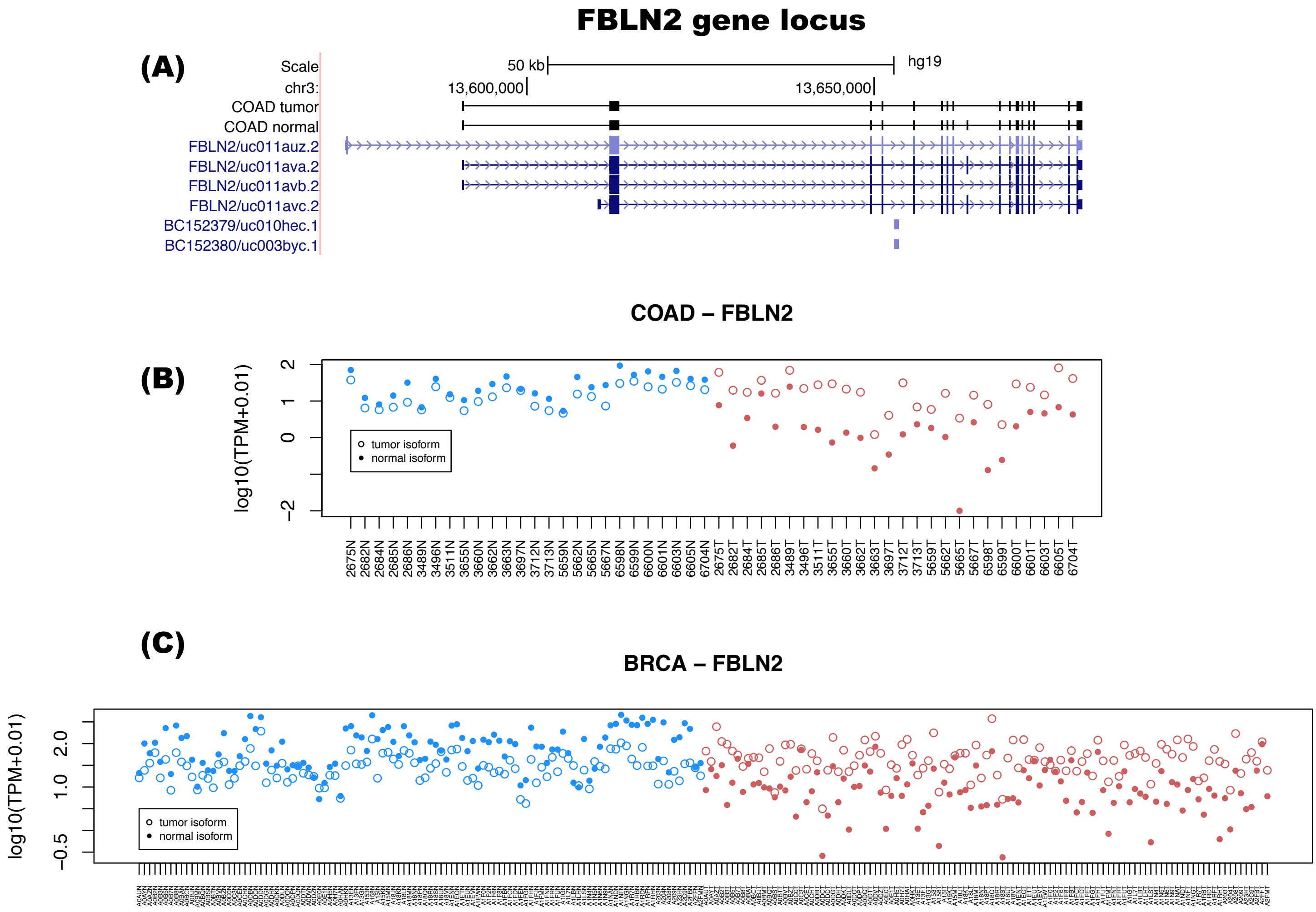

Supplementary Figure 11. (A) UCSC snapshot with the 2 isoforms involved in the isoform switch of FBLN2. The representation does not distinguish between protein coding or non-coding regions of the isoforms. The splicing change corresponds to a protein-coding cassette exon. Representation of the TPMs in log10 scale (y-axis) for both FBLN2 isoforms in COAD **(B)** and BRCA **(C)** samples (x axis)

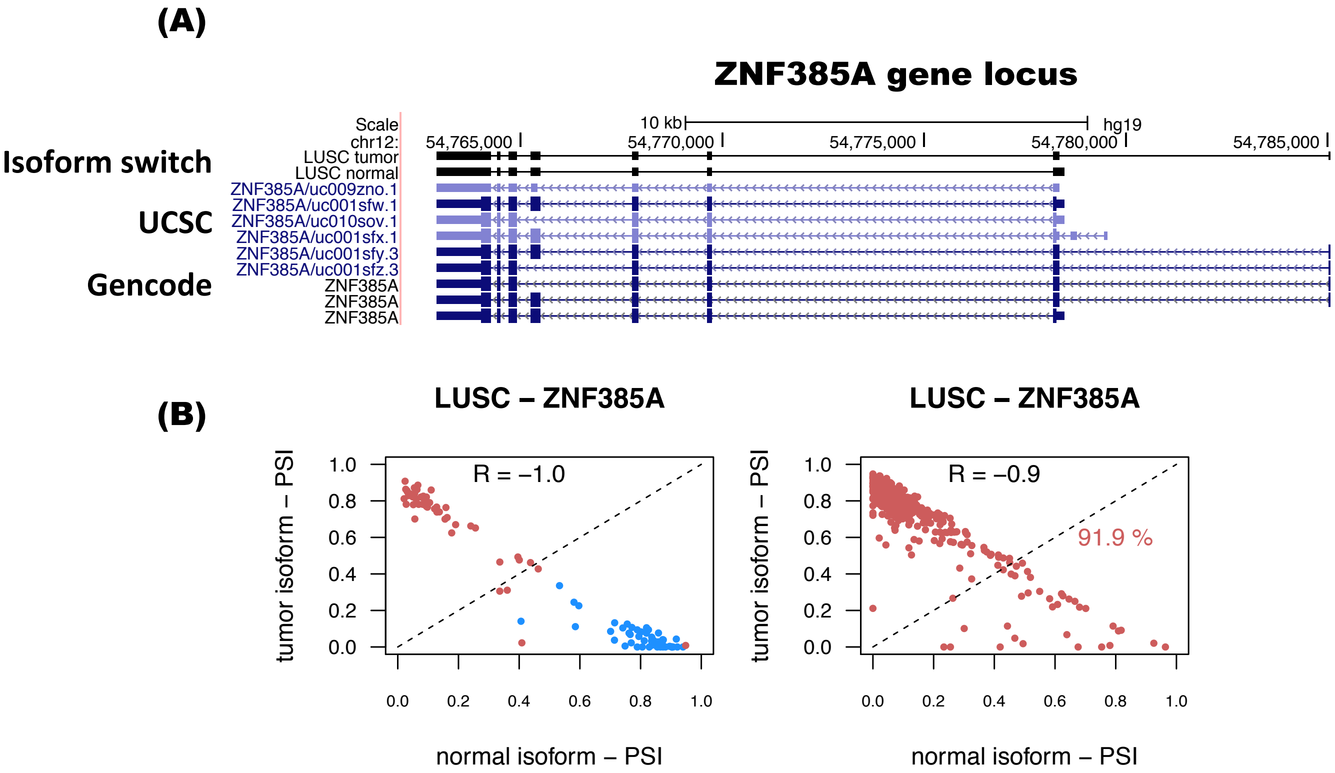

Supplementary Figure 12. There is an isoform switch in LUSC in the gene ZNF385A, which is related to the use of alternative first exon and alternative 3' splice-site. This gene enocdes a protein that interacts with TP53 enhancing its function in cell-cycle arrest, thereby promoting growth arrest (Das et al. 2007). Our representation of the isoform-pair does not distinguish between protein coding or non-coding regions of the isoforms **(B)** Plots of the PSI values for the ZNF385A isoform-pairs (left panel) and unpaired tumor samples (right panel) in LUSC. Values in tumor samples are in red, whereas values in normal samples are in blue. The percentage of correctly classified unpaired tumor samples is given.

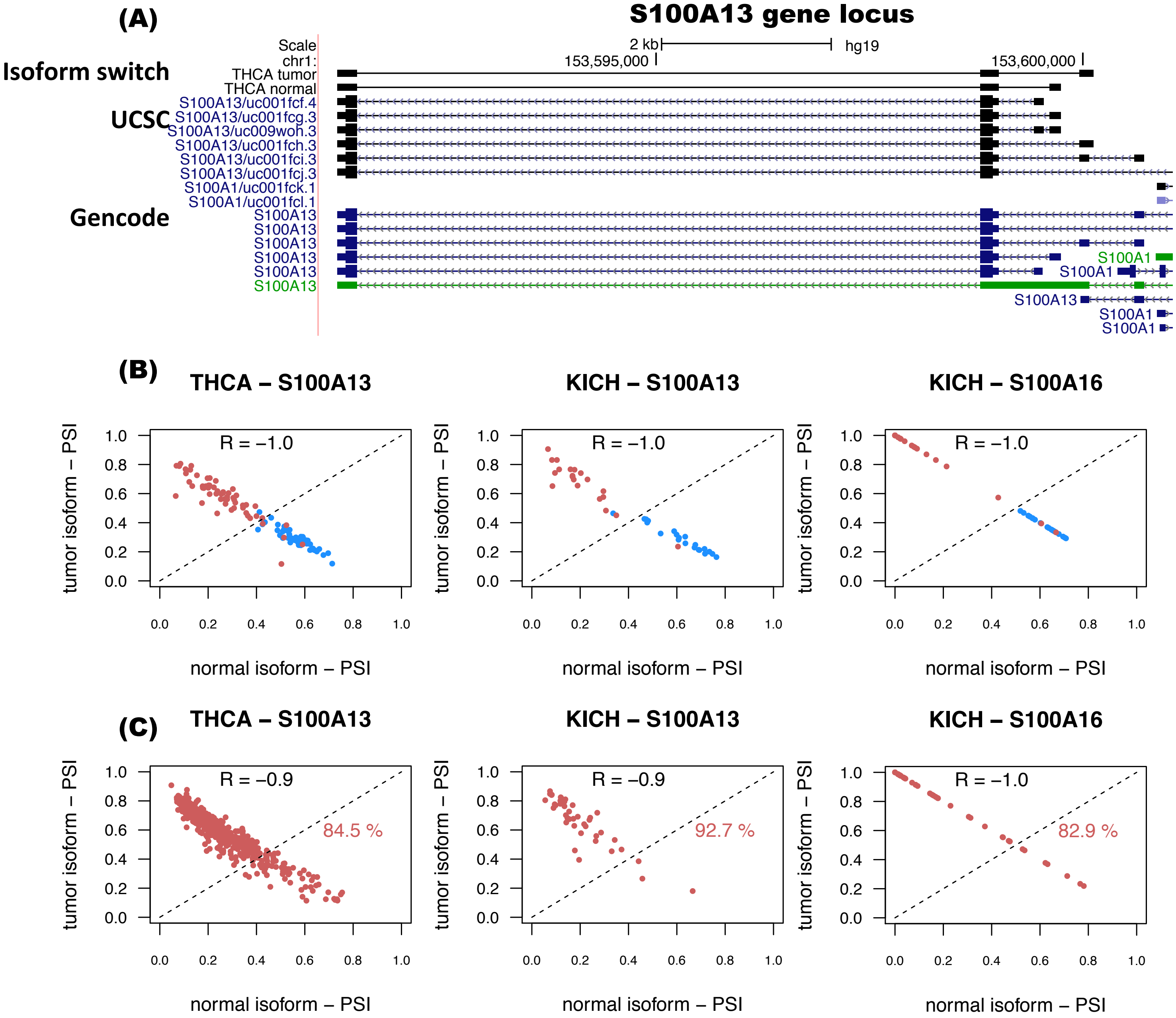

Supplementary Figure 13. (A) The gene S100A13, coding for a calcium binding protein, has multiple isoforms, all differing in the 5' UTR region. The predicted isoform change in S100A13 involves two anti-correlating isoforms differing in a single alternative first 5'UTR exon. S100A13 has been proposed to be a new marker of angiogenesis in various cancer types (Massi et al. 2010). Interestingly, S100A13 and another member of the S100 family, S100A16, have an isoform switch in KICH **(B)** Plots for the relative inclusion of the isoform pairs for S100A13 and S100A16 in unpaired samples The two isoforms show anticorrelation in their relative inclusion levels. **(C)** PSI anticorrelation in the unpaired samples. The S100A13 isoform change alone classifies correctly 84.5% and 92.7% of the THCA and KICH unpaired tumor samples, respectively. Similarly, S100A16 can separate correctly 82.9% of the KICH unpaired samples.

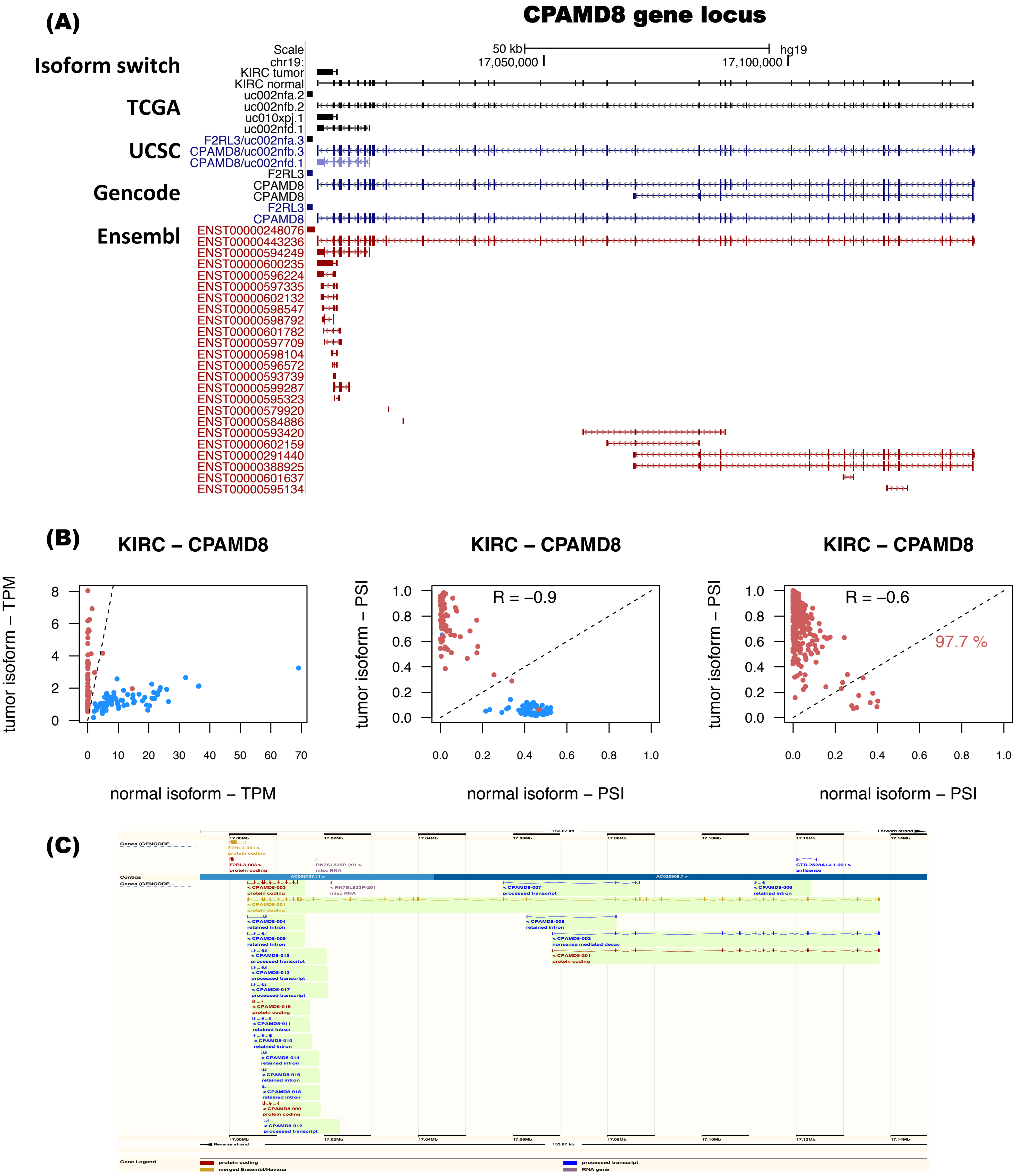

Supplementary Figure 14. CPAMD8 locus. (A) KIRC model is composed of a single isoform switch in CPAMD8, which is implicated in innate immunity and damage control (Li et al. 2004). **(B)** The two isoforms involved show anti-correlation and **(C)** the predicted change gives rise to a truncated isoform that contains a retained intron, however the normal isoform does not reach PSI = 1.

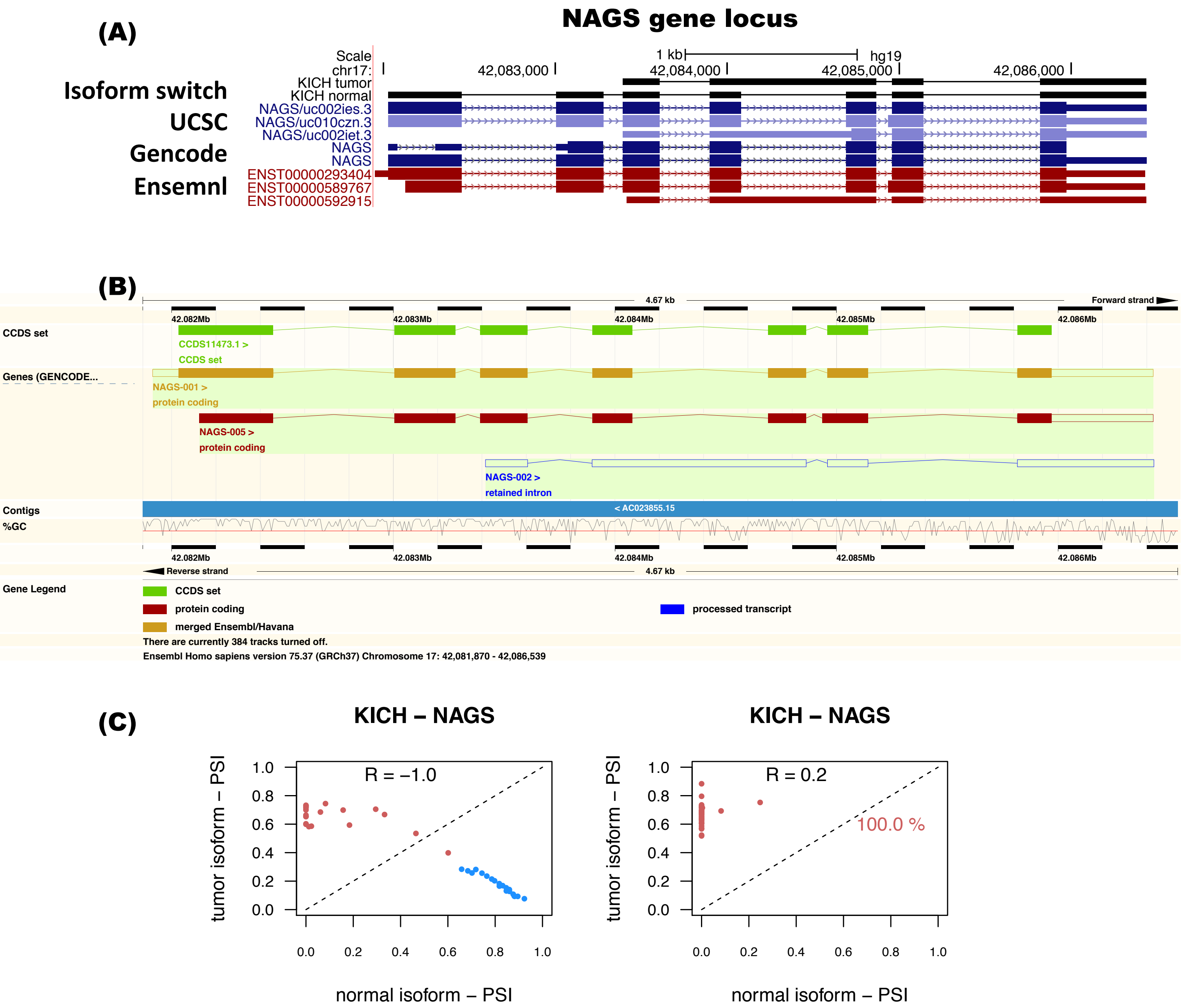

Supplementary Figure 15 .(A) The isoform switch in the N-acetylglutamate synthase involves an intron retention between the 4th and 5th protein exons in KICH tumor samples, which changes a coding region into non-coding in the tumor isoform. **(B)** This transcript is annotated by Ensembl and Havana as an intron-retention without protein product (NAGS-002 ENST00000592915). **(C)** The relative inclusion of the two isofroms anticorrelate in paired and unpaired samples (left and right panels, respectively). In the unpaired samples, 100% of the KICH tumor samples agree with the found isoform rule and show that the normal isoform is not used in most of the samples.

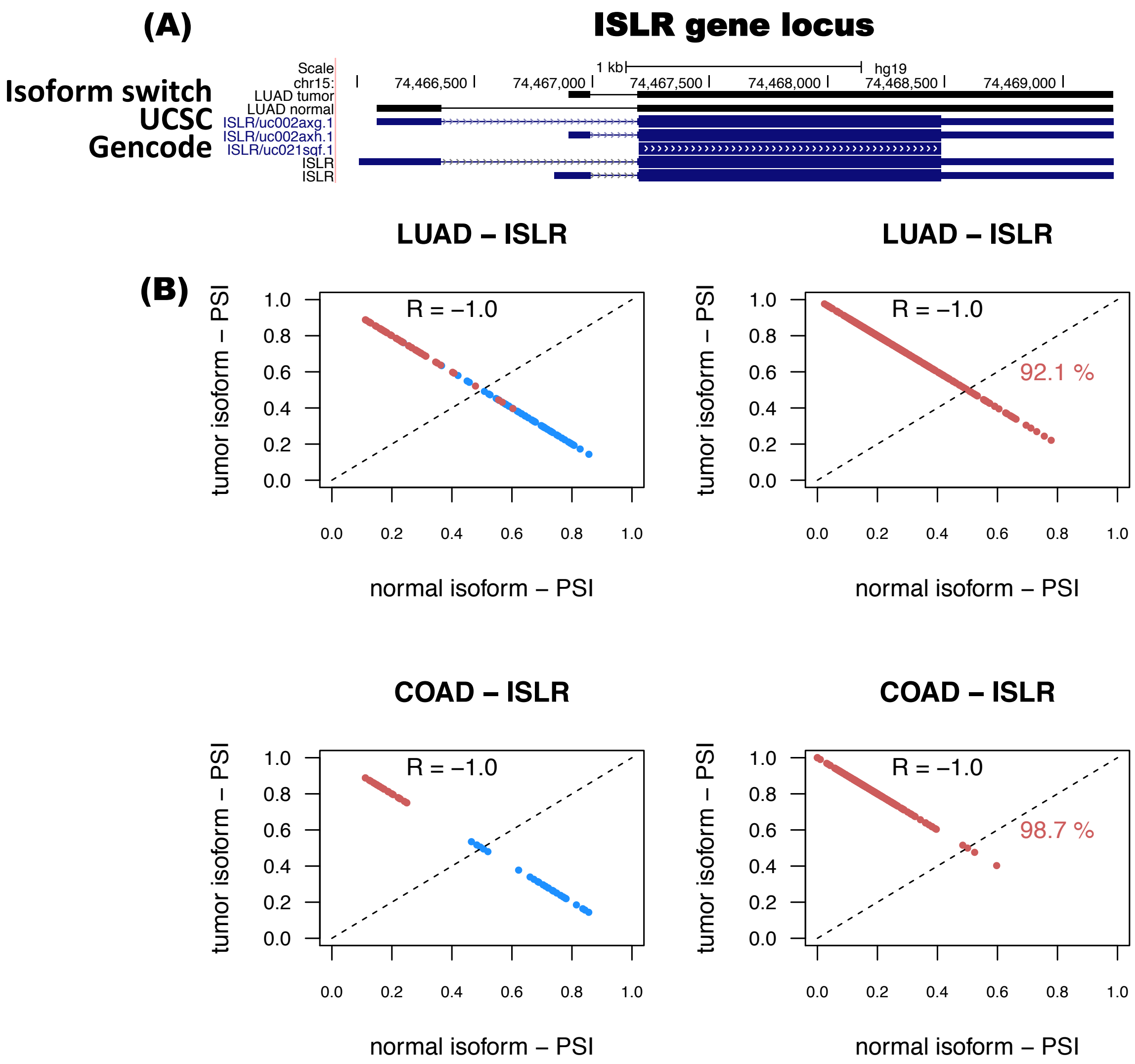

Supplementary Figure 16: LUAD and COAD include a switch in the gene ISLR, involving a change in the first exon **(A)** which shows a perfect anticorrelation in paired and unpaired samples **(B)**. In this case, the strong anticorrelation is due to this gene having only two isoforms. To the best of our knowledge, the activity of this gene has not been related to cancer so far.

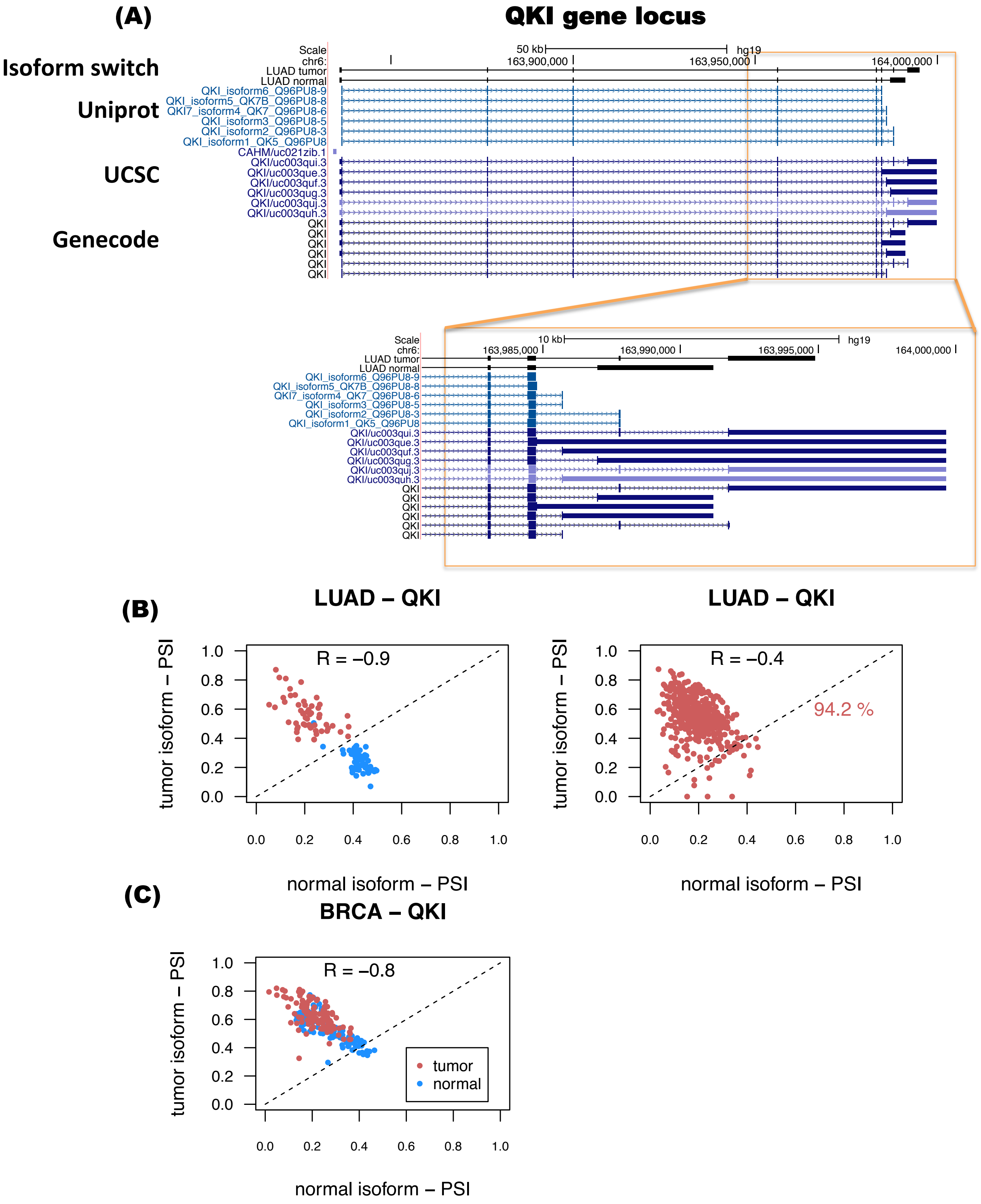

Supplementary Figure 17. (A) The tumoral isoform (uc003qui.2) coincides with NM_006775, which codes for the protein Q96PU8, often denoted as QKI-5. The normal isoform (uc003qug.2) coincides with RefSeq NM_206853, which encodes for protein Q96PU8-9.**(B)** The two isoforms show an anticorrelation pattern. **(C)** The same two isoforms show anticorrelation in BRCA samples, but they show similar inclusion.

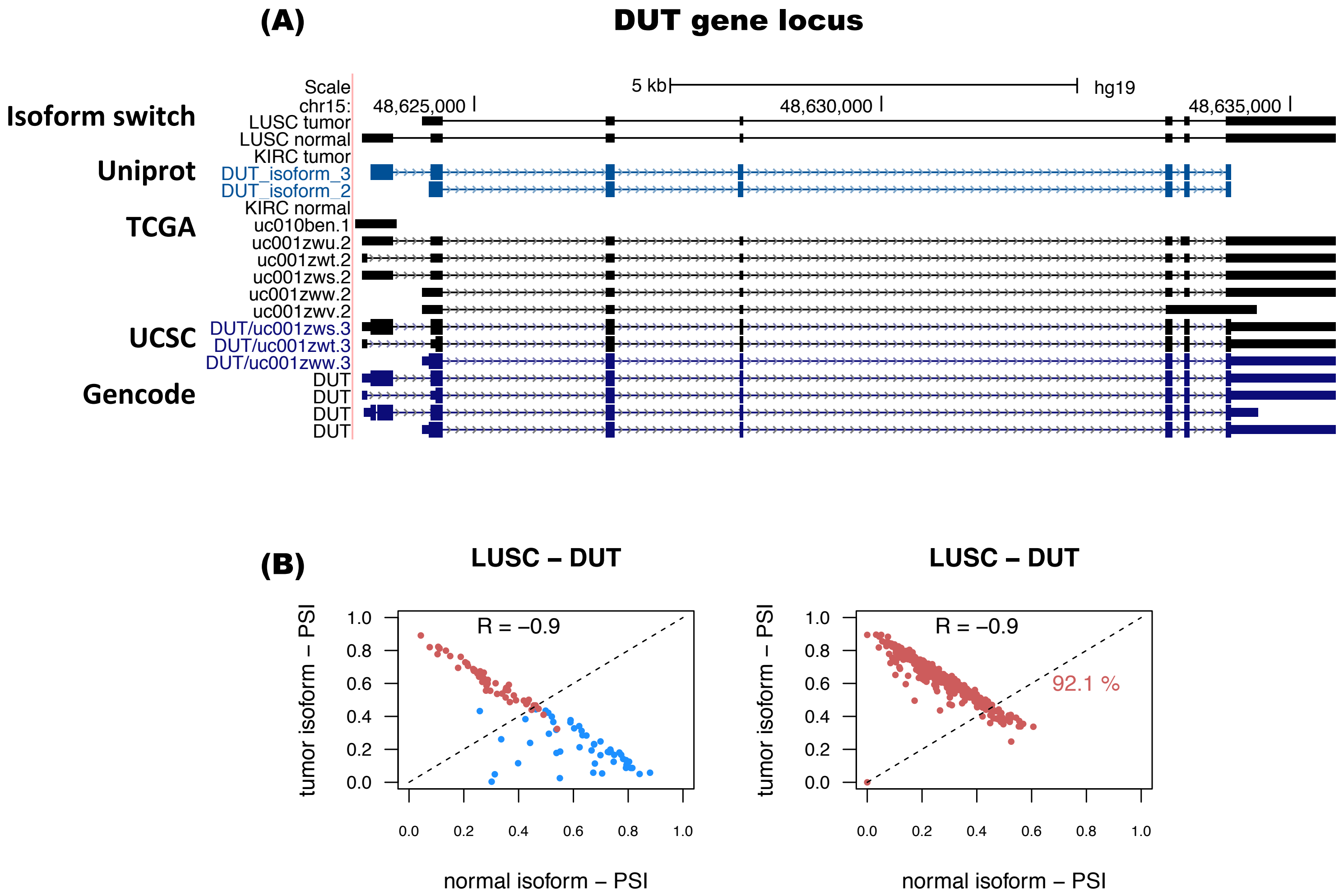

Supplmentary Figure 18. (A) DUT gene has two isoforms, named isoform 2 (Uniprot identifier P33316-2) and isoform 3 (Uniprot identifier P33316). The track "Uniprot" contains the mapping of these two proteins to the DUT genome locus. The isoform switch predicts the RNA encoding for protein isoform 2 to be more included in tumor samples. Protein Isoform 2 has been involved in the onset of DNA replication in lung fibroblasts (Ladner et al. 1997). **(B)** The two isoforms show anti-correlation in their PSIs in paired normal samples (left) and in unpaired samples (right).

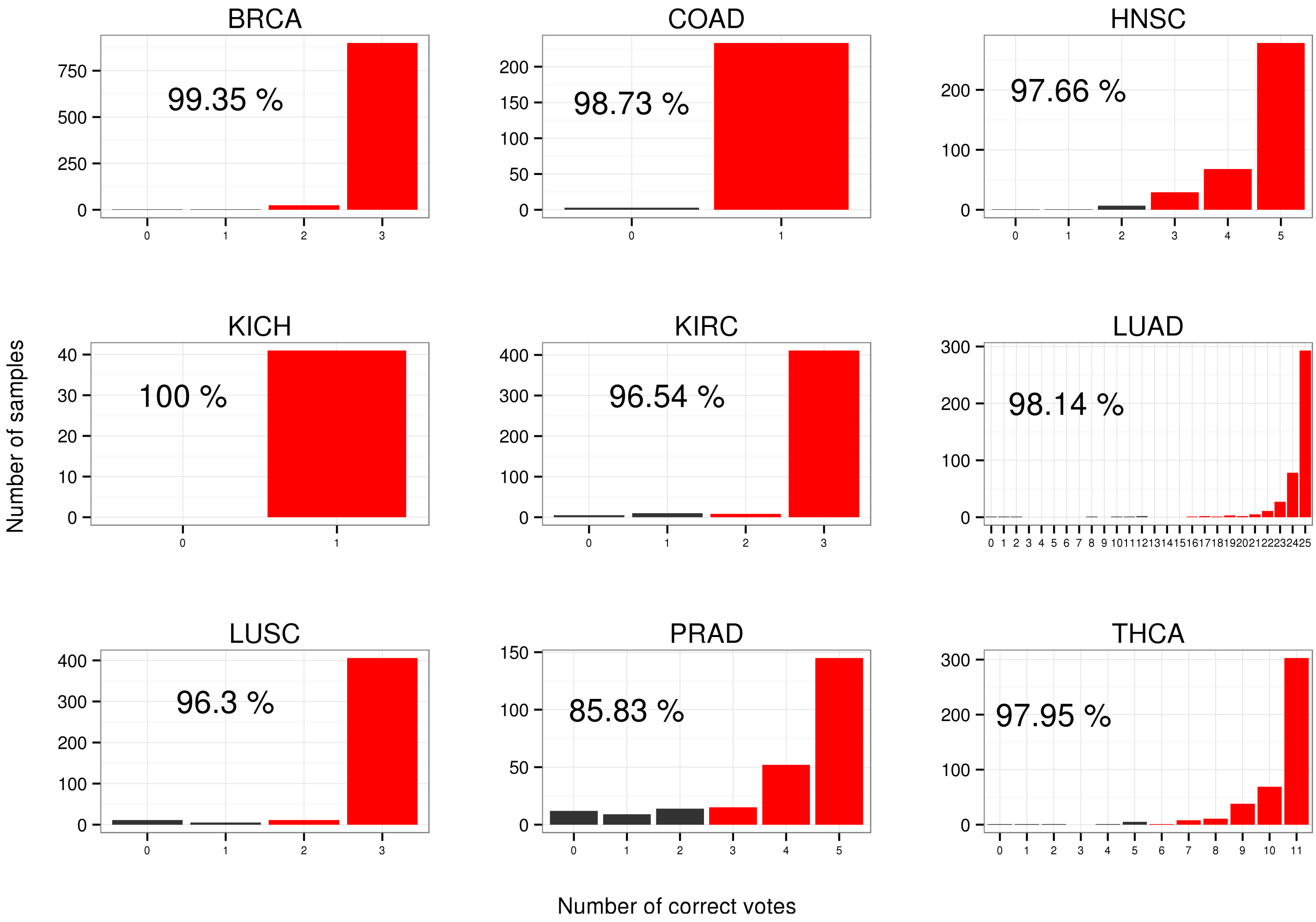

Supplementary Figure 19. Results of the blind-test for the gene-based models. For each cancer type, predictive models were build based on gene expression reversals using the kTSP algorithm using the full gene set available in the TCGA annotation. The models were derived on the set of paired samples and the tests were carried out on the unpaired tumor samples from Table 1. The histograms indicate the proportion of samples (y-axis) for each number of possible correct votes (x-axis), i.e., the number of gene-pair rules from the model. The red bars indicate the number of correct votes that are necessary to classify a sample correctly. Incorrectly labelled samples are indicated with black bars. The percentage of samples correctly classified is also indicated. The kTSP algorithm for genes is implemented as an option in iso-kTSP https://bitbucket.org/regulatorygenomicsupf/iso-ktsp.

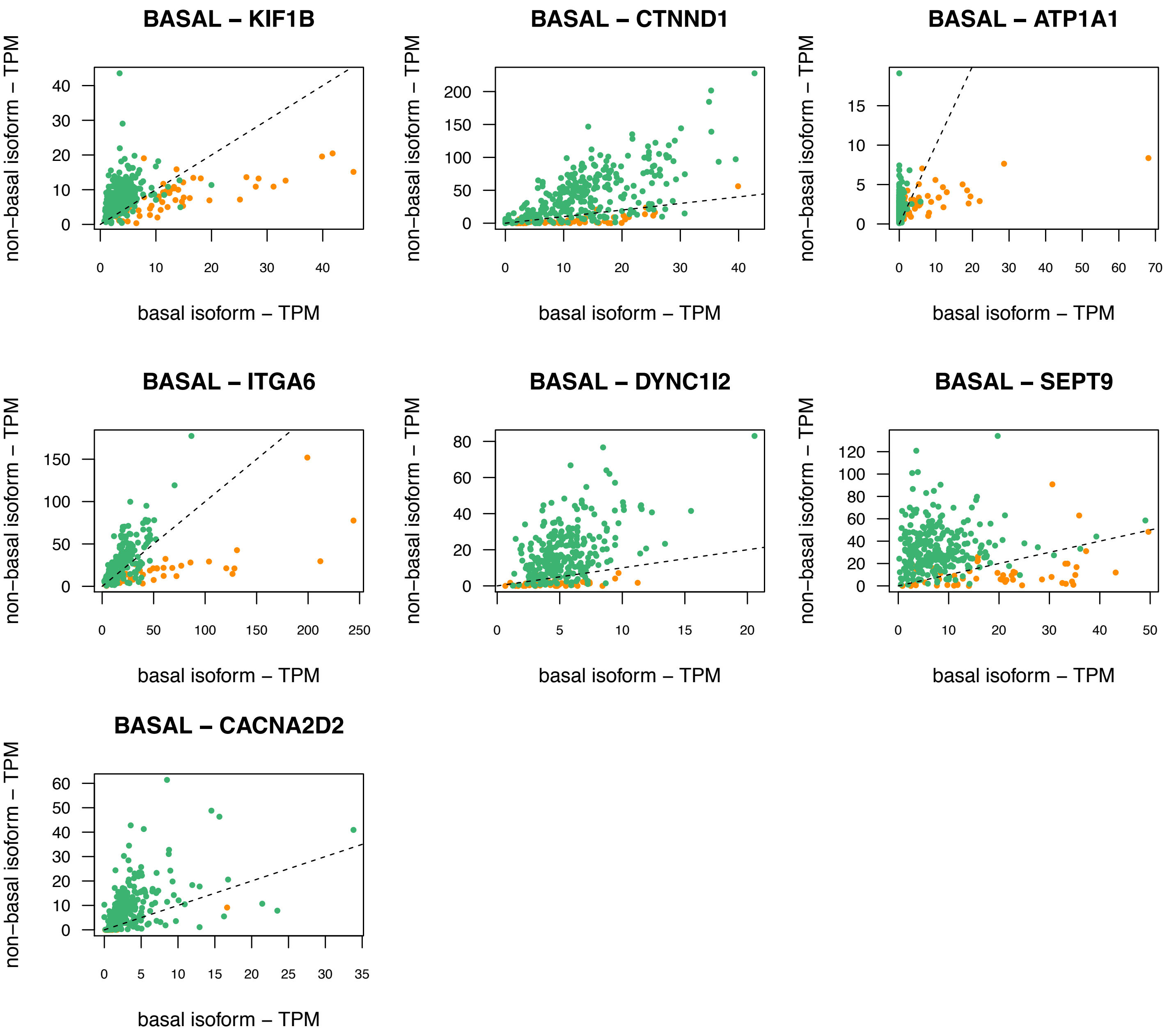

Supplementary Figure 20. **Basal-like breast cancer isoform-pair model.** Transcript expression plots for the Isoform-pair model for basal-like BRCA tumors. The plots show the TPM for the isoform pairs found to be specific to BRCA basal samples compared to the other BRCA subtypes (luminal A, luminal B and Her2+). We show those that show significance in more than 80% of the balanced comparisons using random subsampling. Basal samples are depicted in orange, whereas the samples from the other subtypes are shown in green.

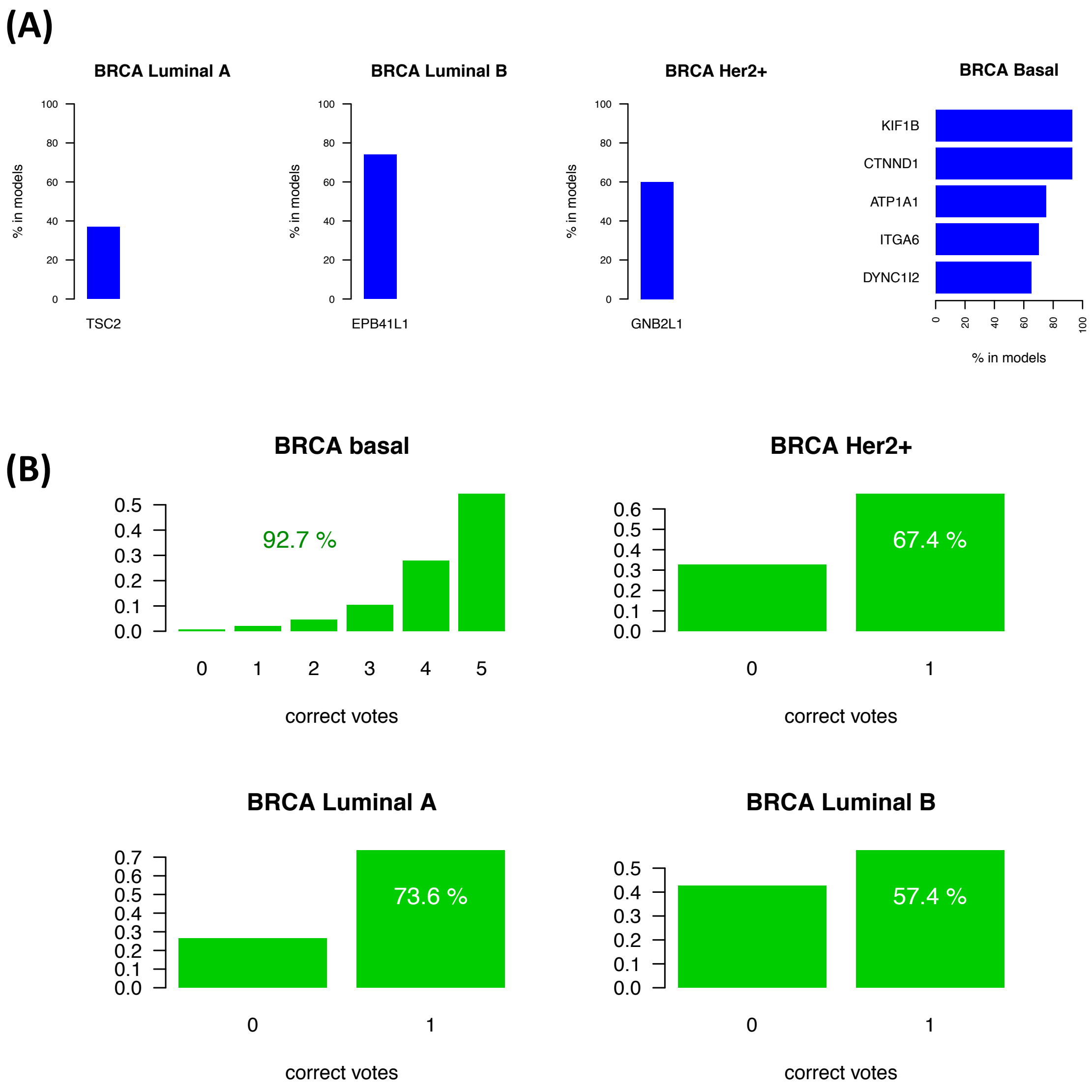

Supplementary Figure 21. Calculated isoform change models for BRCA subtypes (A) Models obtained for the four BRCA subtypes: luminal A, luminal B, Her2+ and basal. The basal model shows the top 5 isoform pairs. **(B)** Performance of each model on the entire dataset. The barplots show the proportion of tested samples with a specific number of correct votes. A true prediction is considered when the majority of votes are correct. The printed percentage refers to the total number of samples that are correctly classified. Basal shows an overall accuracy of 92.% with more than 50% of the samples agreeing with the 5 isoform-pair rules.

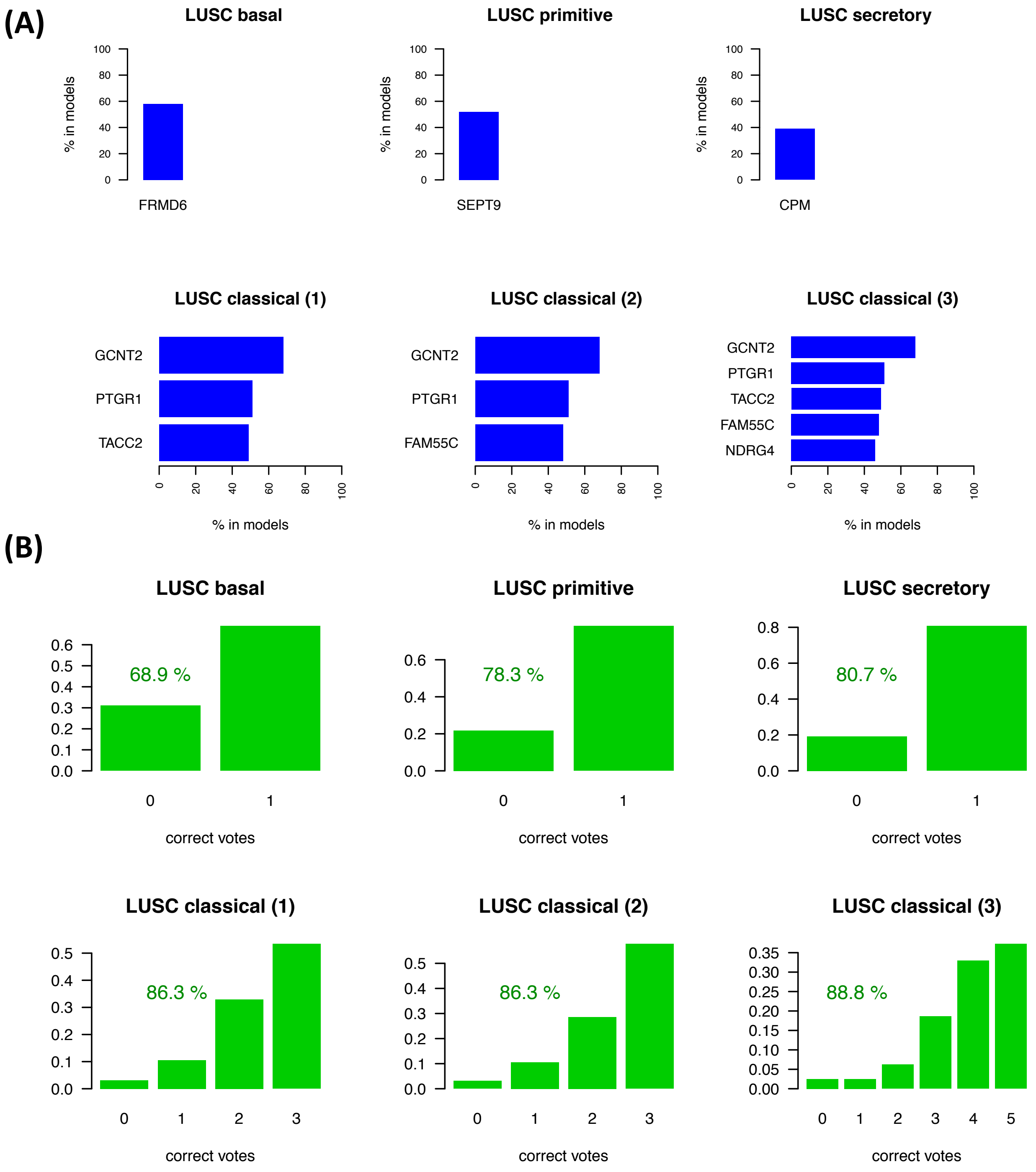

Supplementary Figure 22. Calculated isoform change models for LUSC subtypes. (A) Basal, primitive and secretory show almost no recurrent changes. We considered the top ones for testing. For the classical subtype we considered 3 models with 3 and 5 isoform changes that include those genes that appeared in more than 40% of the models: PTGR1, TACC2, FAM55C and NDRG4. Except for GCNT2, which was significant in 22% of the iterations, all other isoform changes were significant in at most 3% of them. The figure indicates the fraction of random subsampling iterations (comparing one subtype against a balanced pool from the rest) for which each gene was selected in a predictive model. **(B)** Performance of the models when tested on the entire LUSC tumor set, comparing each subtype vs all the rest. The barplots show the number of samples with each possible number of correct votes (from 0 to the number of genes in the model), and the percentage of samples correctly classified.

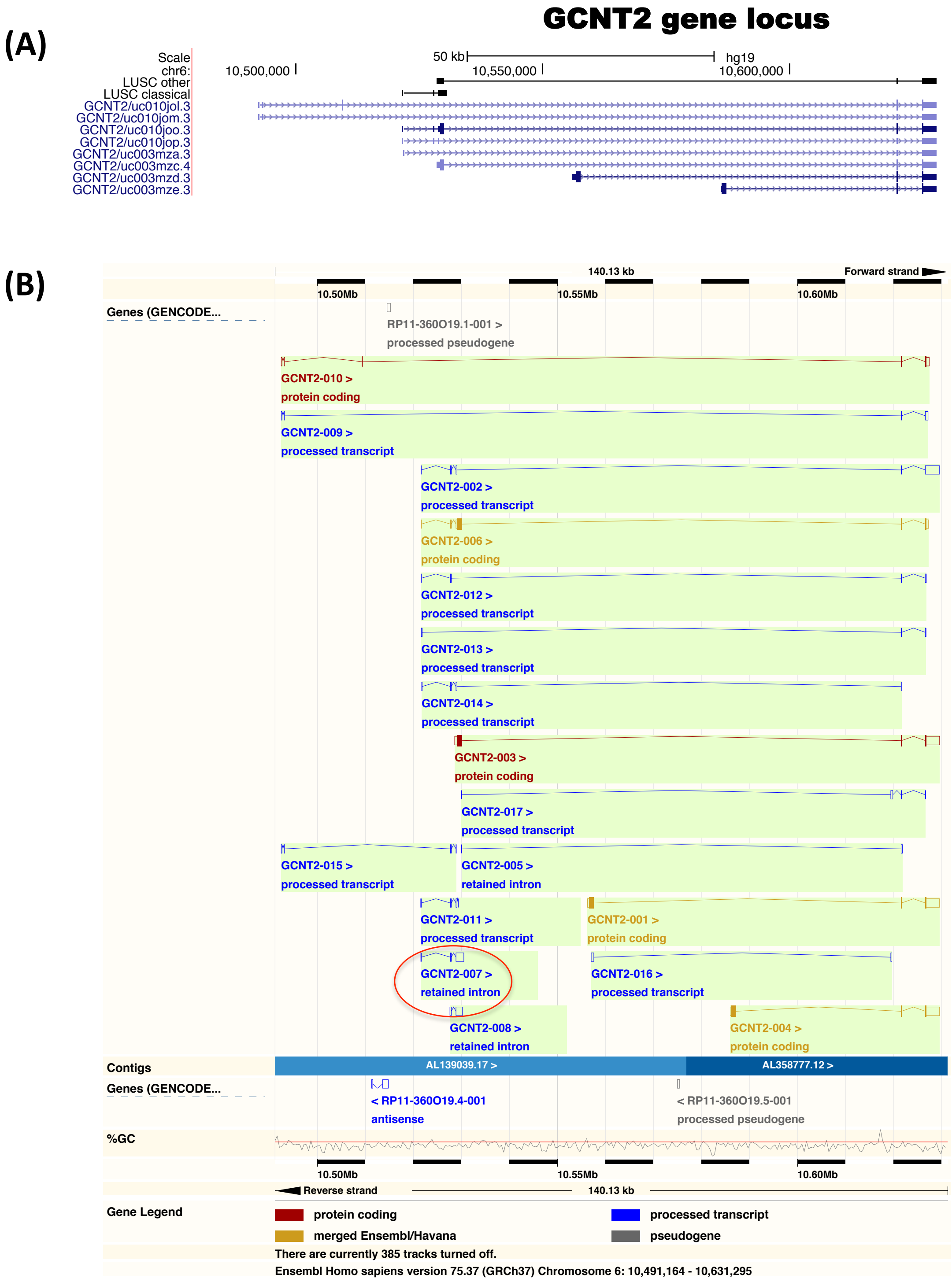

Supplementary Figure 23. (A) Isoform change predicted for GCNT2 between the classical and the other LUSC subtypes. Isoforms are represented in black, without indicating coding or non-coding regions. **(B)** The isoform frequently found in the classical samples corresponds to an isoform annotated as non-coding and with a retained intron by Ensembl.

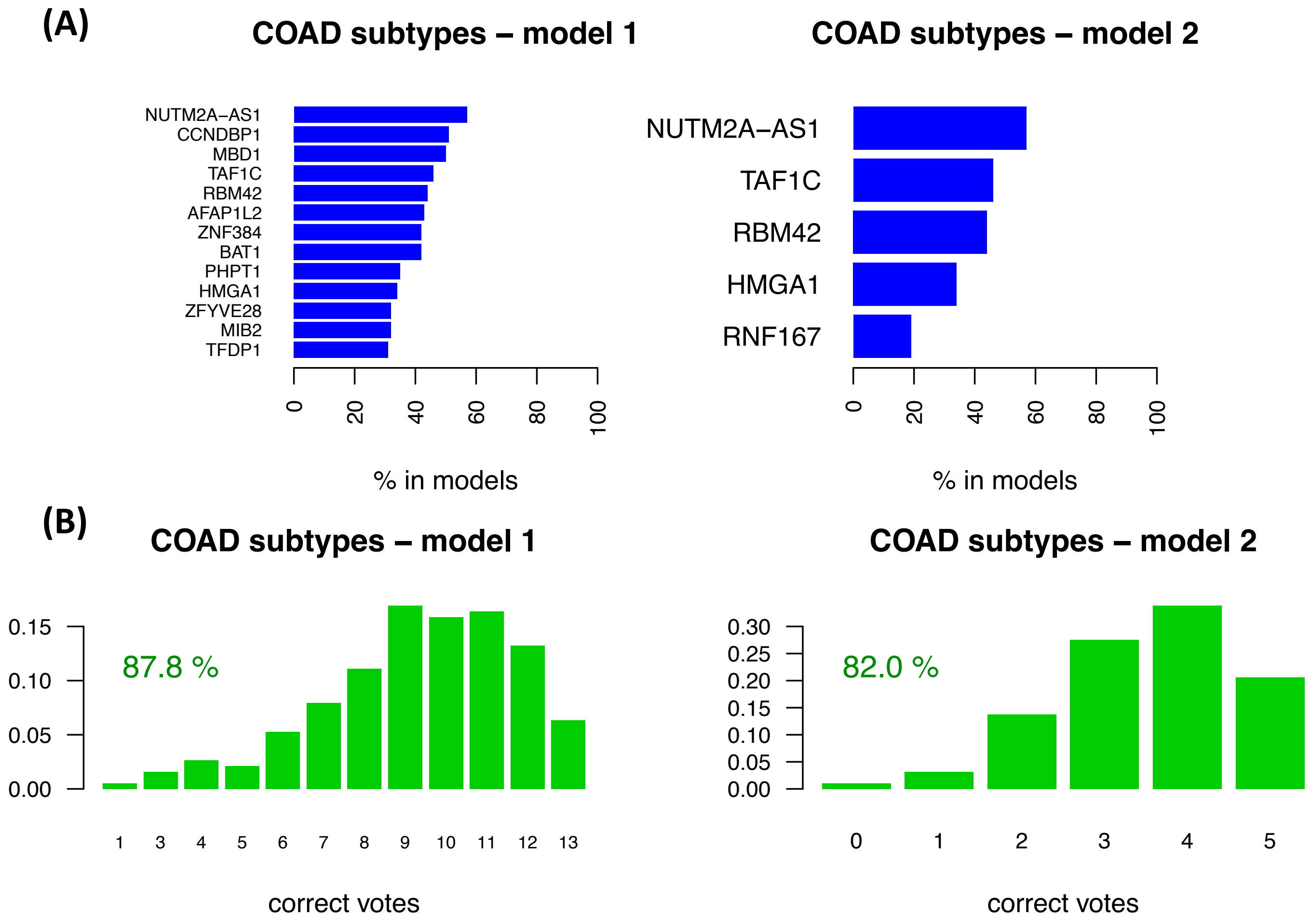

Supplementary Figure 24. Calculated isoform changes between COAD hyper and non-hyper mutated samples (A) We considered two models with 13 and 5 isoform-pairs that appeared in models for more than 20% of the iterations. None of the found isoform-pairs were significant for more than 4% of the samples. **(B)** Performance of the two models when tested on the entire COAD tumor set, separating hyper and non-hyper mutated samples. The barplots show the number of samples with each possible number of correct votes (from 0 to the number of genes in the model), and the percentage of samples correctly classified.

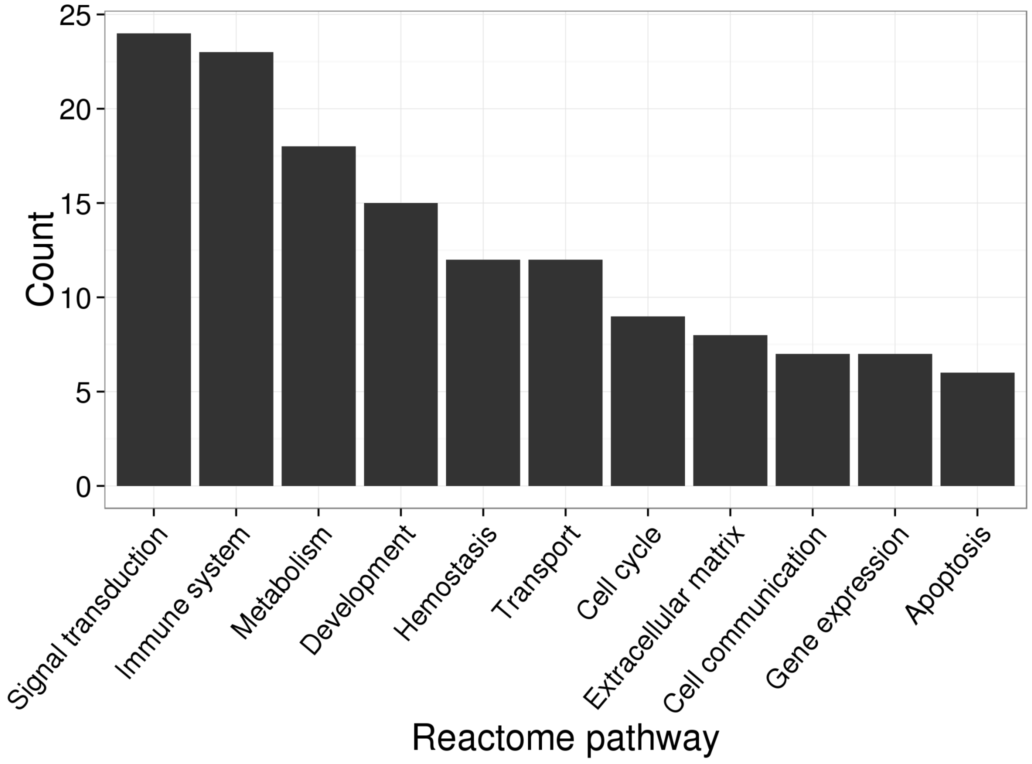

Supplementary Figure 25. Reactome pathway analysis. The barplot shows the most frequent Reactome pathways represented by the 244 isoform switches (Figure 5), based on the v68 of the Reactome database. None of these pathways show a significant enrichment compared to the total number of genes with multiple isoforms as background.

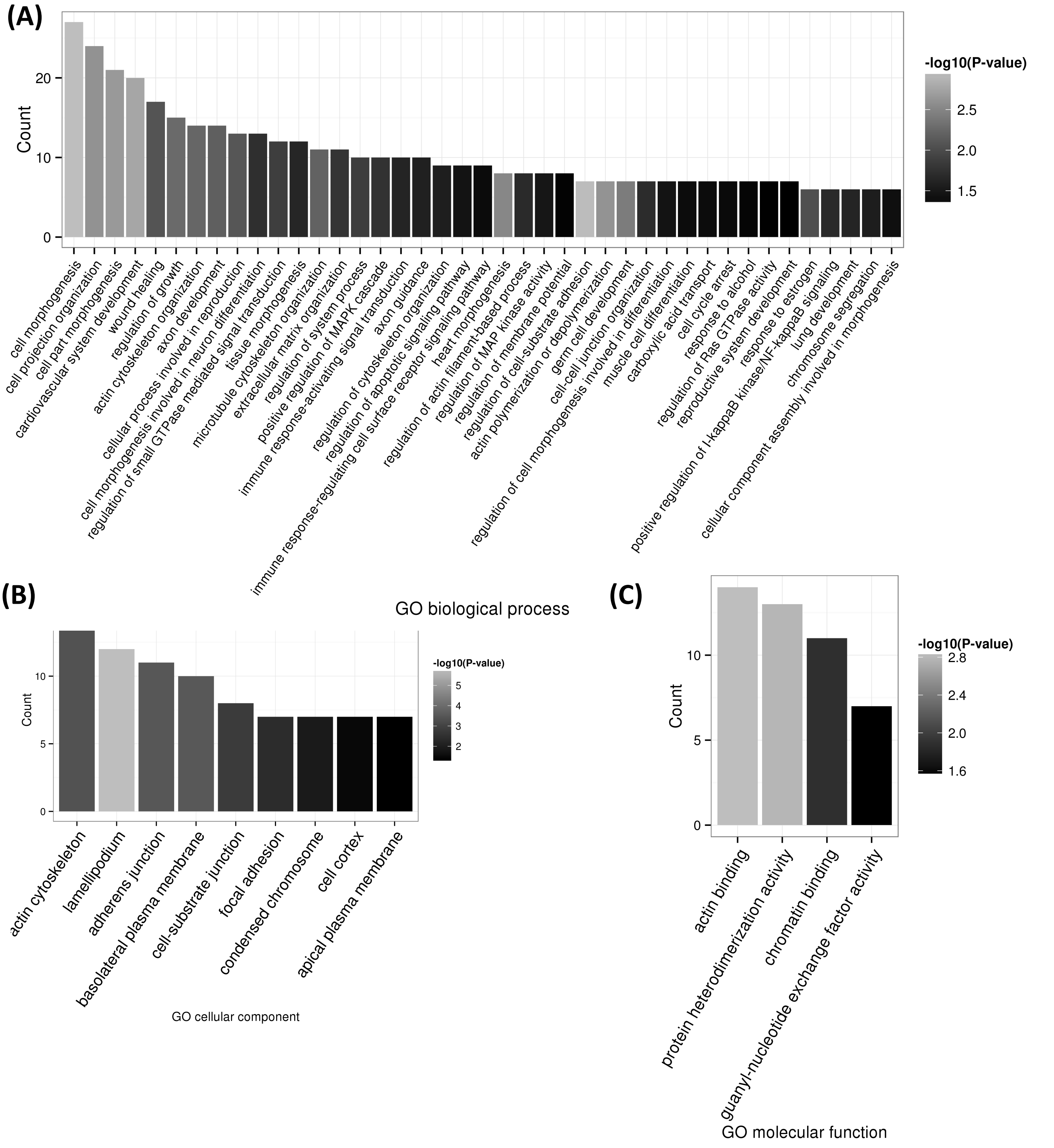

Supplementary Figure 26. GO enrichment analysis. The barplots show the most frequent GO terms represented by the 244 isoform switches for Biological Process **(A)**, Cellular Component **(B)** and Molecular Function **(C)** ontologies. The GOstat package was used for the test, with a P-value cutoff of 0.05, odds-ratio > 2 and at least 5 genes. The background set was the gene set used for the Iso-kTSP analysis and the "conditional" option was used in the test to calculate the significance of a parent term, if its children were significant. All the shown categories are significant. The gray scale indicates the –log10(p-value).

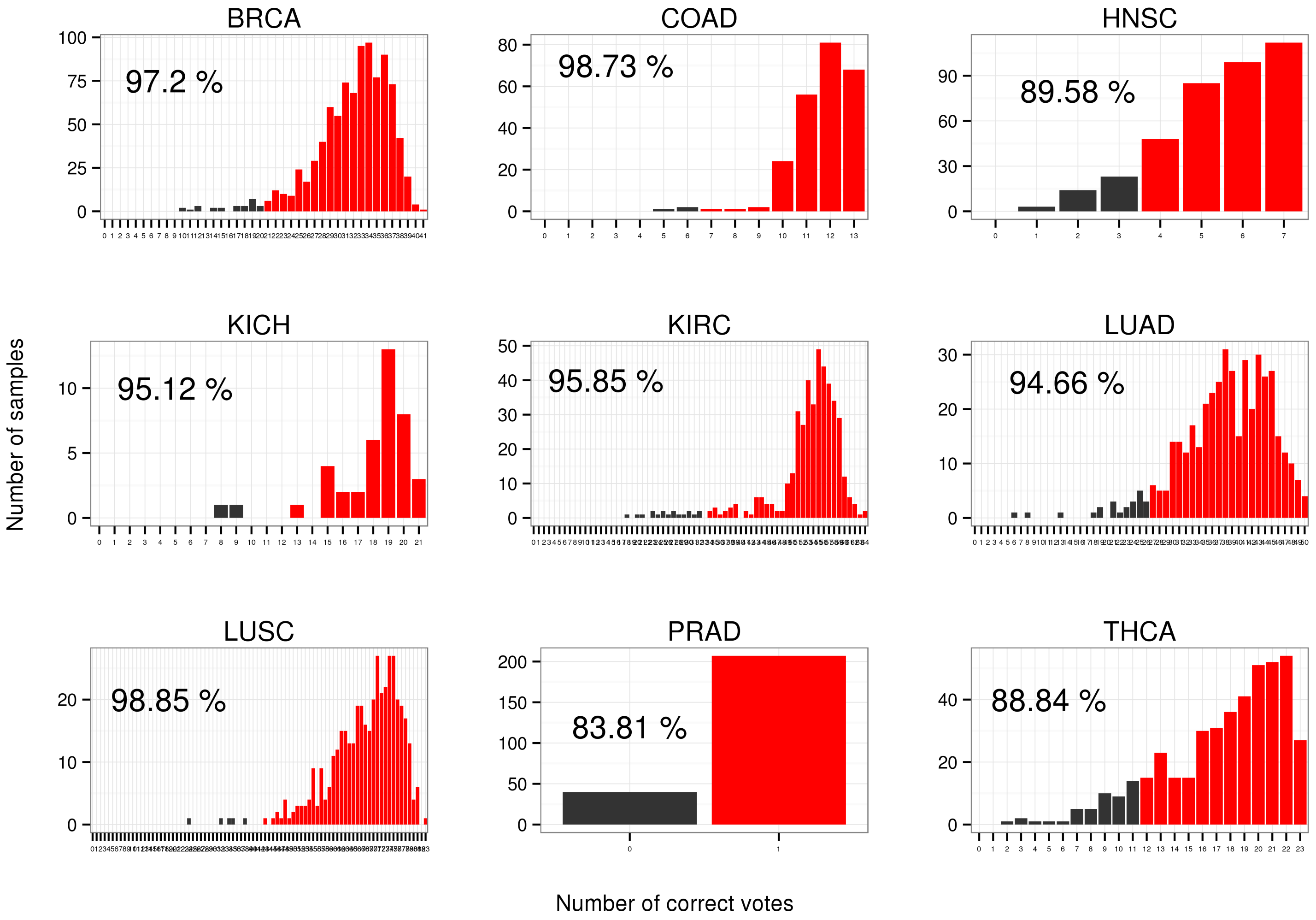

Supplementary Figure 27. Performance of the isoform switches on the unpaired tumor samples. For each cancer type, we tested the models built by using all significant isoform switches found in that cancer type. In each cancer type we considered all switches if they were an odd number, or removed the last one from the ranking of Information Gain if they were an even number. The density plots show the number of samples (y axis) according to the number of possible correct votes (x-axis). The bars in red indicate that the number of correct votes is sufficient for correct labeling of a sample (≥ (|switches|+1)/2). The black bars indicate the samples for which the number of correct votes is not sufficient. PRAD is a special case as it has two isoform switches, GALK2 and WASF3. The model testes was for the switch in WASF3. The percentage of samples correctly classified is also indicated.

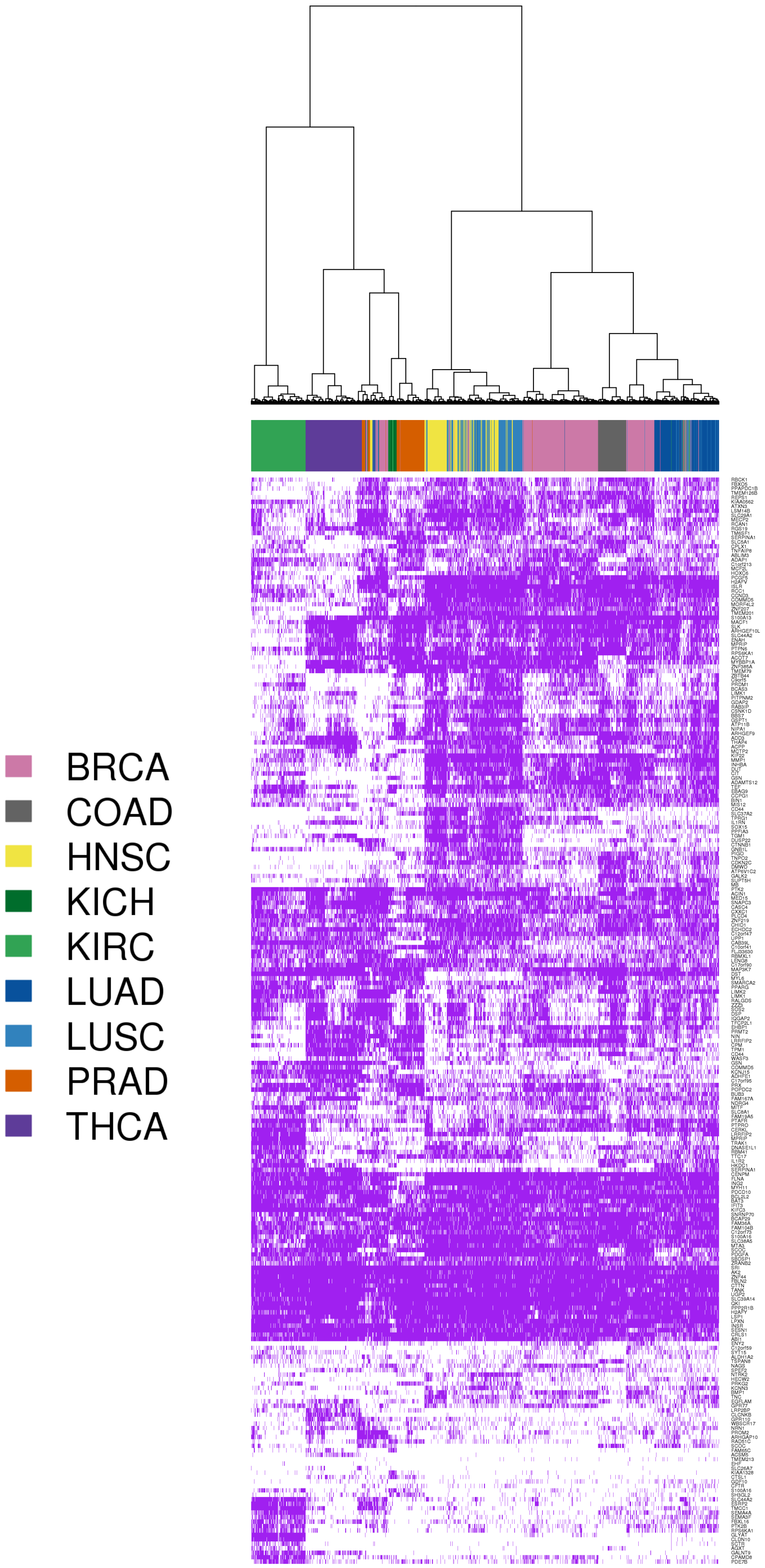

Supplementary figure 28. Unsupervised clustering of tumor samples according to the presence of isoform switches The heatmap shows for each sample (paired and unpaired tumor samples) (x axis) all 244 isoform switches (y-axis) present in that sample (purple), irrespectively of whether the switch was predicted as significant in that cancer type. We indicate in white if the switch is absent in a sample. Only tumor isoforms with TPM>1 are considered as present in each sample. Clustering was performed using the Ward method with the euclidean distance between sample vectors, where the presence or absence of each switch is indicated with 1 or 0, respectively. The cancer types are indicated by a different color for each sample.

(A)	Isoform-pair changes					
	iso-kTSP only	SwitchSeq only	both	Proportion of iso-kTSP in common	Proportion of SwitchSeq in common	correlation of common isoform-pairs
BRCA	397	533	190	0,32	0,26	0,57
COAD	14	720	21	0,60	0,03	0,63
HNSC	14	600	9	0,39	0,01	0,26
KICH	19	1130	56	0,75	0,05	0,17
KIRC	162	686	147	0,48	0,18	0,54
LUAD	133	630	109	0,45	0,15	0,51
LUSC	178	740	171	0,49	0,19	0,43
PRAD	9	411	3	0,25	0,01	0,87
THCA	57	901	41	0,42	0,04	0,61

(B)	Isoform switches					
	iso-kTSP only	SwitchSeq only	both	Proportion of iso-kTSP in common	Proportion of SwitchSeq in common	correlation of common isoform-pairs
BRCA	4	533	38	0,90	0,07	0,94
COAD	0	720	13	1,00	0,02	0,94
HNSC	0	600	7	1,00	0,01	0,47
KICH	0	1130	21	1,00	0,02	0,78
KIRC	5	686	60	0,92	0,08	0,90
LUAD	1	630	53	0,98	0,08	0,94
LUSC	5	740	80	0,94	0,10	0,91
PRAD	0	411	2	1,00	0,00	1,00
THCA	1	901	22	0,96	0,02	0,75

Supplementary Table 1. (A) Comparison of our significant isoform-pairs (Figure 1D) with the SwitchSeq isoform-pairs calculated from the same input data. The table shows that cases that are unique to each method and common to both, and the respective proportions. The table also shows the correlation for the common isoform-pairs using our score S_1 and the score provided by SwitchSeq. The column for both corresponds to all dots (black and red) in Supplementary Figure 28. **(B)** Comparison of our isoform-pairs predicted as proper isoform switches (significant isoform-pairs anticorrelation < -0.8 between the PSI values of the two isoforms and having an average TPM > 1 for at least one of the isoforms, both in the tumor and normal samples), with the SwitchSeq isoform pairs. The column for both corresponds to the red dots in Supplementary Figure 28. The last column indicates as above the correlation between both scores for the common isoform-pairs.

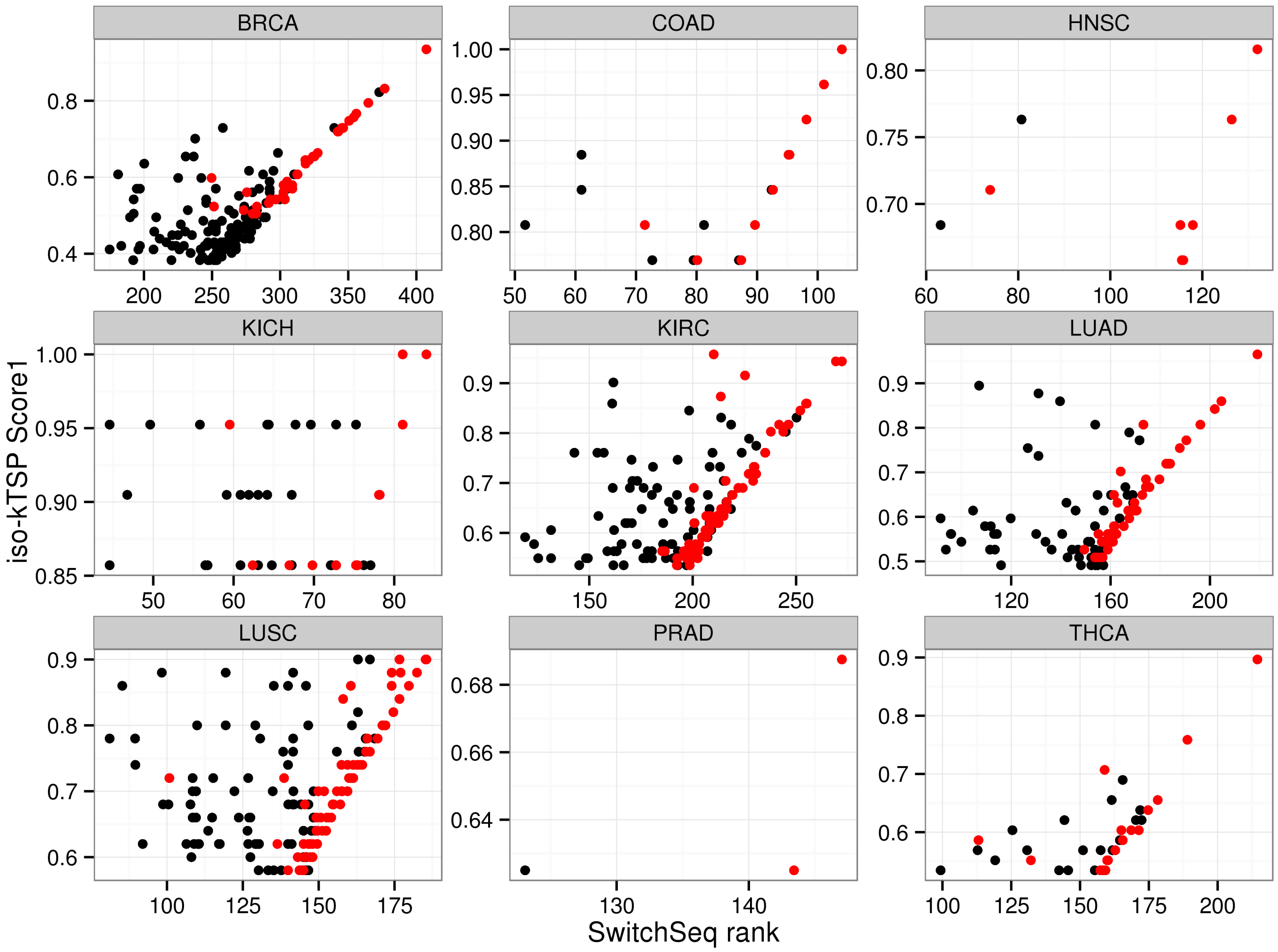

Supplementary Figure 29. Comparison of significant isoform-pairs calculated with Iso-kTSP with the isoform-pairs from SwitchSeq. Correlation of the isoform pairs in different genes found by SwitchSeq and iso-kTSP. The x-axis shows the SwitchSeq rank and the y-axis shows the iso-kTSP score S_1. All dots correspond to the isoform-pairs that are found by both methods. The red dots highlight the subset of isoform pairs that are defined as switches by our method, meaning an anticorrelation < -0.8 between the PSI values of the two isoforms and having an average TPM > 1 for at least one of the isoforms, both in the tumor and normal samples.

cancer	RNA-Seq	Mutation Data	Overlap
**BRCA**	1036	977	969
**COAD**	262	270	210
**HNSC**	422	306	303
**KICH**	62	66	62
**KIRC**	505	417	412
**LUAD**	488	519	459
**LUSC**	483	178	178
**PRAD**	295	251	247
**THCA**	497	402	400

Supplementary Table 2. Samples with RNA-Seq data and with mutation data from DNA sequencing (either whole-genome sequencing or Exome-Seq). Last column shows the total number of samples with both RNA-Seq and DNA sequencing data that were used for the mutation analysis.

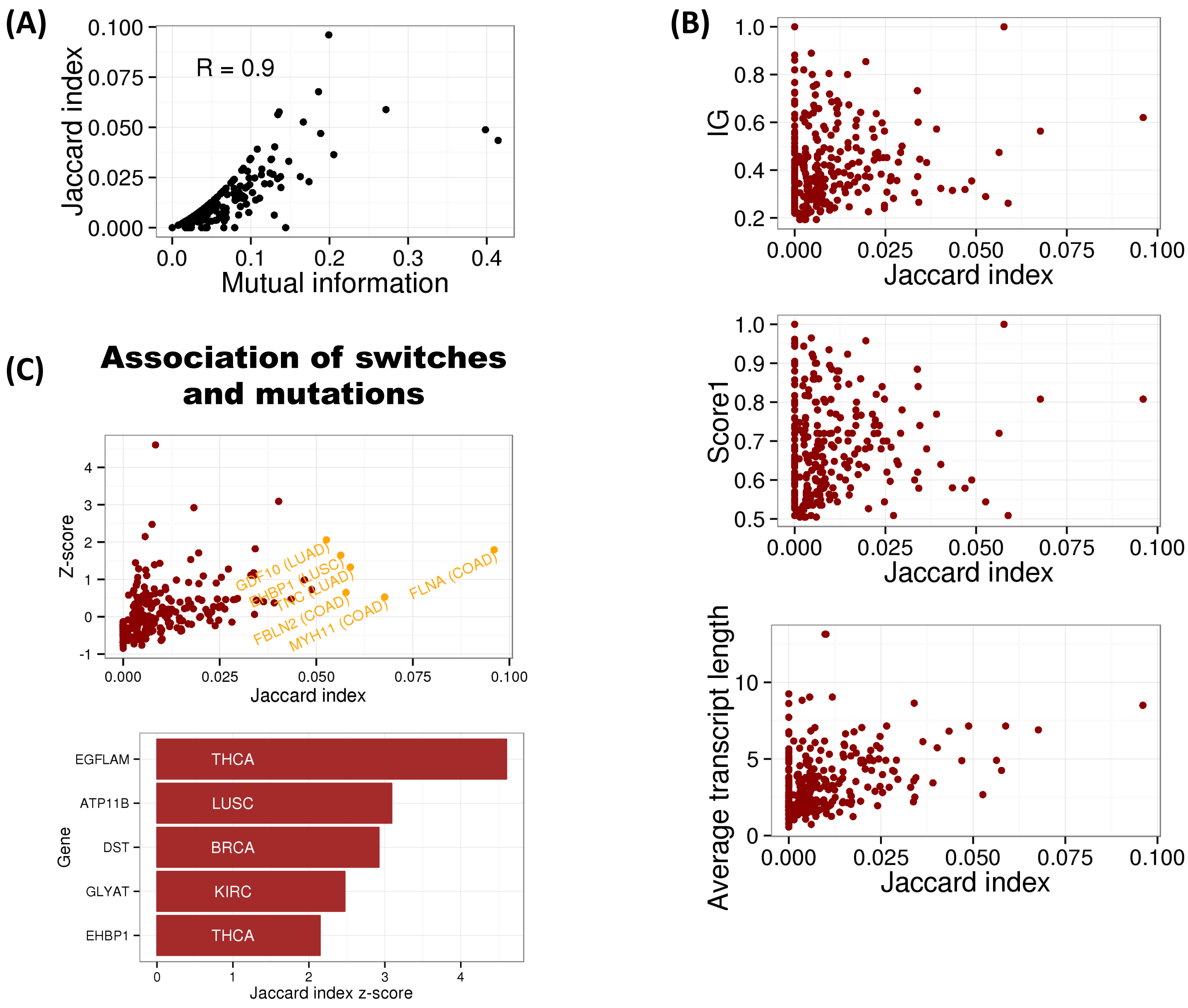

Supplementary Figure 30. (A) Correlation between Jaccard indexes and mutual information. In both cases the association is calculated across samples, using for each sample the presence or absence of the switch and the presence or absence of one or more mutations. **(B)** Relation between the Jaccard index and three characterizing variables of the isoform switches: Information gain of the switch, score S_1 and transcript length (averaged for both isoforms in the pair). The plots show that there is no correlation between these variables and the Jaccard index. **(C)** Relation between Jaccard indexes and the corresponding z-scores, calculated by comparing to the Jaccard index in the same cancer type to a set of 100 random transcripts with similar total exonic length. In the plot we indicate the position of the switches in FLNA, MYH11, FBLN2, TNC, EHBP1 and GDF10. The barplot below shows the Z-scores, which tend to be higher for switches in cancer with very few mutations. That is, the top z-scores are due to 1 or 2 mutated samples only. These plots were performed with all mutation types falling in isoforms. Similar results are obtained when using all mutations falling on the full gene region, and when using only protein-affecting mutations on isoforms or gene-regions.

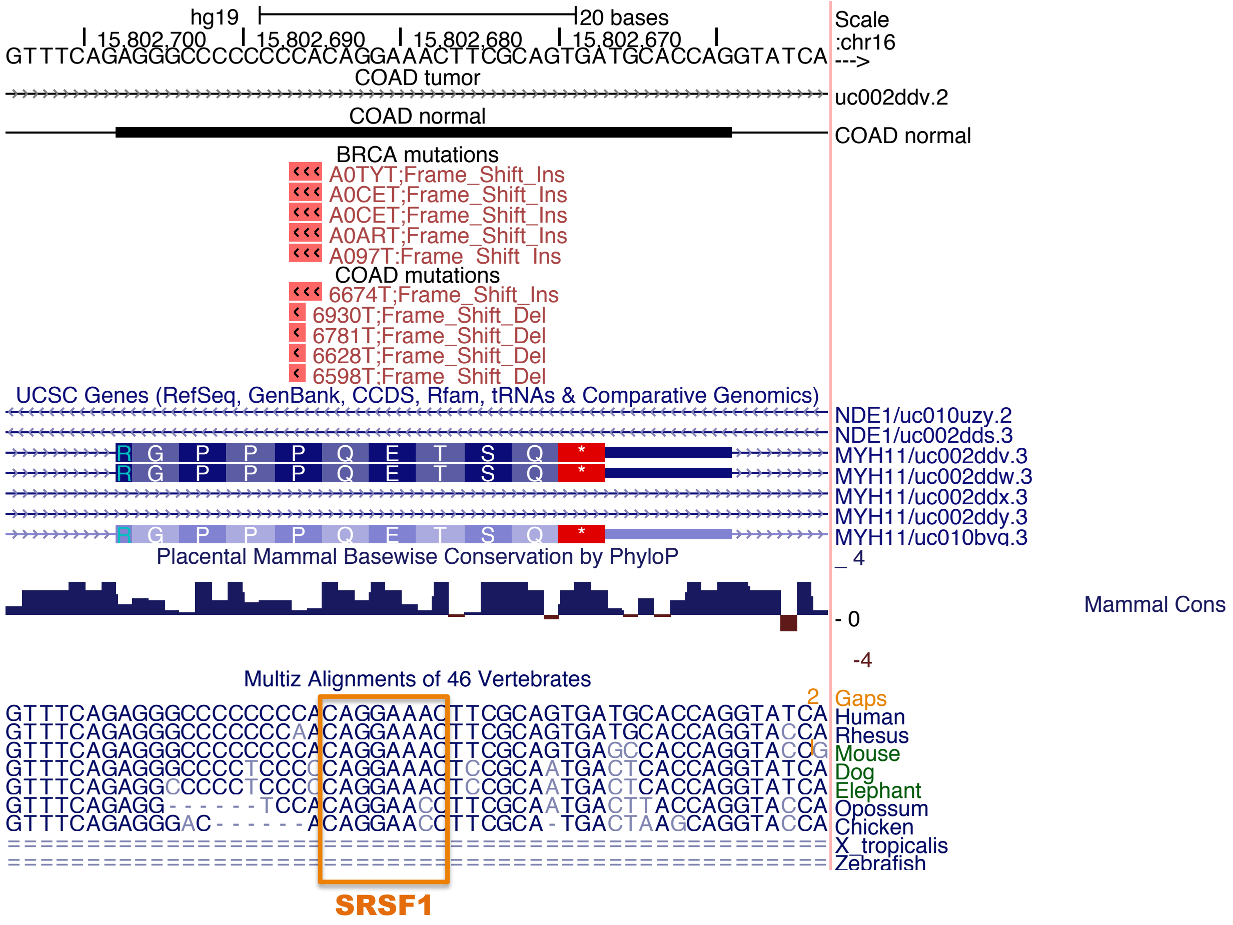

Supplementary Figure 31. Somatic mutations found in COAD and BRCA samples on the alternative exon of MYH11 involved in the isoform switch. The frameshift insertions and deletions fall in a region of low conservation. Right next to these indels we find a putative SRSF1 binding site, with score 7.901 and p-value = 0.000903425. The binding site was predicted using the the program FIMO from the MEME suite and the motif matrix from the RNAcompete datasets.

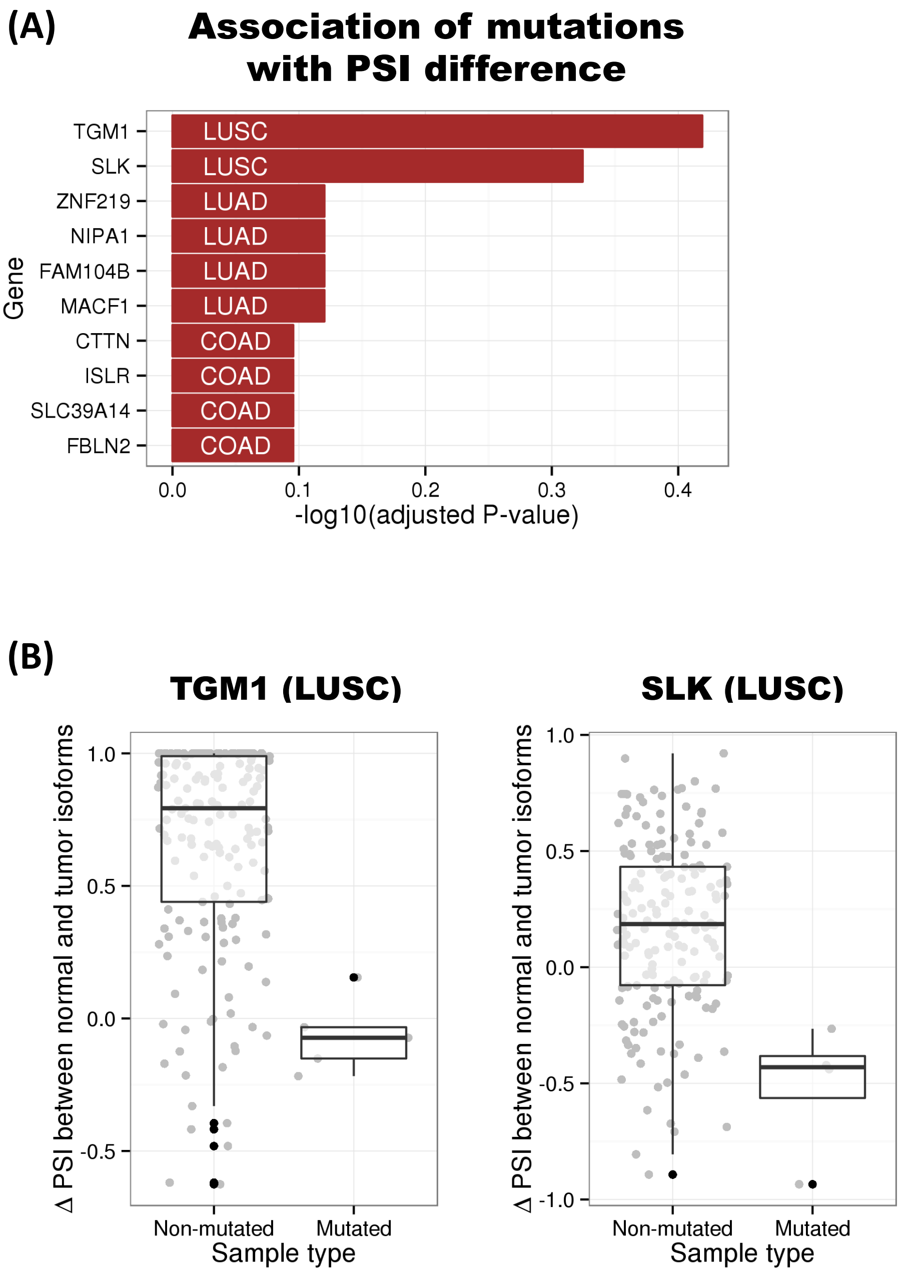

Supplementary Figure 32. (A) Ranking of p-values for the Mann-Whitney test comparing the distribution of PSI differences between tumor and normal isoforms of a switch in mutated and non-mutated tumor samples. After correcting multiple testing, none of these cases are significant with p-value < 0.05. **(B)** Distribution of the PSI difference of tumor and normal isoforms for the switch in TGM1 and SLK in LUSC, for mutated and non-mutated tumor samples. A difference can be appreciated, but the number of mutated samples is too small to draw any reliable conclusion.

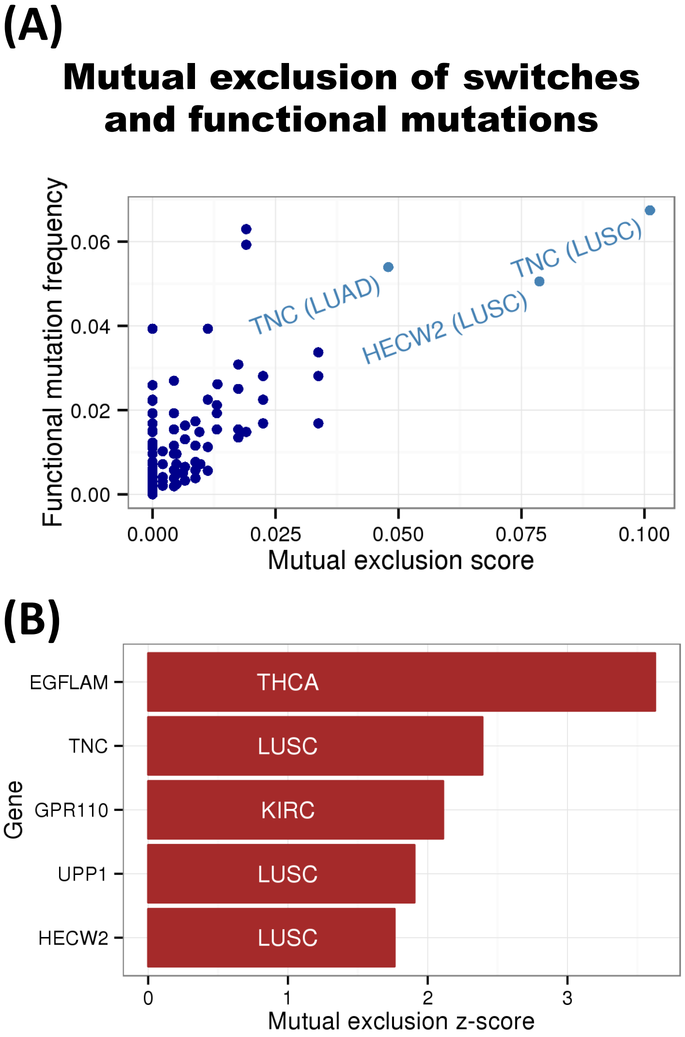

(C)

TNC (LUSC)	No mut	mut
No switch	98	9
Switch	68	3
HECW2 (LUSC)	No mut	mut
No switch	112	7
Switch	57	2
TNC (LUAD)	No mut	mut
No switch	256	11
Switch	183	9

Supplementary Figure 33. (A) Relation between mutual-exclusion score and frequency of mutated samples (using only CDS-affecting mutations). The mutual-exclusion score measures the proportion of samples that have no switch and have a mutation affecting the protein coding sequence (missense, nonsense, frameshift and indel). **(B)** Ranking of z-scores for the mutual-exclusion scores. Z-scores are calculated as for other measures by comparing to the mutual-exclusion score for 100 random isoforms with similar transcript (total exonic) length using mutations from the same cancer type. Some of the top z-scores (e.g. EGFLAM in THCA) occur in switches in cancer types with very few mutations and are due to just one or two cases. Accordingly, we did not consider the z-score as a reliable way to select the relevant cases. **(B)** Number of samples with or without the switch and with (mut) or without (No mut) mutations affecting the protein coding sequence for the three top ranking scores from (A). These top ranking cases show more samples with mutation and no switch, than samples with both mutation and switch. However, there are many more for which there is a switch but no mutation.

Supplementary Methods

RNA-Seq data analysis

Available processed RNA-Seq data for tumor and normal samples was downloaded from the TCGA data portal (https://tcga-data.nci.nih.gov/tcga/) for breast carcinoma (BRCA), colon adenocarcinoma (COAD) head and neck squamous cell carcinoma (HNSC), kidney cromophobe carcinoma (KICH), kidney renal clear cell carcinoma (KIRC), lung adenocarcinoma (LUAD), lung squamous cell carcinoma (LUSC), prostate adenocarcinoma (PRAD) and thyroid carcinoma (THCA). In all cases version 2 and Level 3 of the data was used. We used the hg19 version of the UCSC annotation from June 2011.

Download locations:

(1)/brca/cgcc/unc.edu/illuminahiseq_rnaseqv2/rnaseqv2/unc.edu_BRCA.IlluminaHiSeq_RNASeqV2.Level_3.1.7.0.tar.gz
(1)/brca/cgcc/unc.edu/illuminahiseq_rnaseqv2/rnaseqv2/unc.edu_BRCA.IlluminaHiSeq_RNASeqV2.mage-tab.1.8.0.tar.gz
(1)/coad/cgcc/unc.edu/illuminahiseq_rnaseqv2/rnaseqv2/unc.edu_COAD.IlluminaHiSeq_RNASeqV2.Level_3.1.7.0.tar.gz
(1)/coad/cgcc/unc.edu/illuminahiseq_rnaseqv2/rnaseqv2/unc.edu_COAD.IlluminaHiSeq_RNASeqV2.mage-tab.1.7.0.tar.gz
(1)/hnsc/cgcc/unc.edu/illuminahiseq_rnaseqv2/rnaseqv2/unc.edu_HNSC.IlluminaHiSeq_RNASeqV2.Level_3.1.5.0.tar.gz
(1)/hnsc/cgcc/unc.edu/illuminahiseq_rnaseqv2/rnaseqv2/unc.edu_HNSC.IlluminaHiSeq_RNASeqV2.mage-tab.1.6.0.tar.gz
(1)/kich/cgcc/unc.edu/illuminahiseq_rnaseqv2/rnaseqv2/unc.edu_KICH.IlluminaHiSeq_RNASeqV2.mage-tab.1.2.0.tar.gz
(1)/kich/cgcc/unc.edu/illuminahiseq_rnaseqv2/rnaseqv2/unc.edu_KICH.IlluminaHiSeq_RNASeqV2.Level_3.1.1.0.tar.gz
(1)/kirc/cgcc/unc.edu/illuminahiseq_rnaseqv2/rnaseqv2/unc.edu_KIRC.IlluminaHiSeq_RNASeqV2.mage-tab.1.4.0.tar.gz
(1)/kirc/cgcc/unc.edu/illuminahiseq_rnaseqv2/rnaseqv2/unc.edu_KIRC.IlluminaHiSeq_RNASeqV2.Level_3.1.3.0.tar.gz
(1)/kirp/cgcc/unc.edu/illuminahiseq_rnaseqv2/rnaseqv2/unc.edu_KIRP.IlluminaHiSeq_RNASeqV2.mage-tab.1.11.0.tar.gz
(1)/luad/cgcc/unc.edu/illuminahiseq_rnaseqv2/rnaseqv2/unc.edu_LUAD.IlluminaHiSeq_RNASeqV2.Level_3.1.12.0.tar.gz
(1)/luad/cgcc/unc.edu/illuminahiseq_rnaseqv2/rnaseqv2/unc.edu_LUAD.IlluminaHiSeq_RNASeqV2.mage-tab.1.13.0.tar.gz
(1)/lusc/cgcc/unc.edu/illuminahiseq_rnaseqv2/rnaseqv2/unc.edu_LUSC.IlluminaHiSeq_RNASeqV2.mage-tab.1.9.0.tar.gz
(1)/lusc/cgcc/unc.edu/illuminahiseq_rnaseqv2/rnaseqv2/unc.edu_LUSC.IlluminaHiSeq_RNASeqV2.Level_3.1.7.0.tar.gz
(1)/prad/cgcc/unc.edu/illuminahiseq_rnaseqv2/rnaseqv2/unc.edu_PRAD.IlluminaHiSeq_RNASeqV2.mage-tab.1.10.0.tar.gz
(1)/prad/cgcc/unc.edu/illuminahiseq_rnaseqv2/rnaseqv2/unc.edu_PRAD.IlluminaHiSeq_RNASeqV2.Level_3.1.8.0.tar.gz
(1)/thca/cgcc/unc.edu/illuminahiseq_rnaseqv2/rnaseqv2/unc.edu_THCA.IlluminaHiSeq_RNASeqV2.mage-tab.1.12.0.tar.gz
(1)/thca/cgcc/unc.edu/illuminahiseq_rnaseqv2/rnaseqv2/unc.edu_THCA.IlluminaHiSeq_RNASeqV2.Level_3.1.11.0.tar.gz

where

(1) = https://tcga-data.nci.nih.gov/tcgafiles/ftp_auth/distro_ftpusers/anonymous/tumor

To assess sample quality, TCGA-provided estimated read-counts per gene were used to predict tissue type patterns with URSA (Lee et al. 2013) (Supplementary Figure 1) and we removed samples from the paired dataset that did not cluster with the rest of the samples from the same class (either normal or tumor). We also analysed the non-paired tumor samples, but did not remove any of the samples.

We selected all those cancer types that were not under embargo at the time of performing the analysis and for which we had paired normal samples for the same patients. From those we kept only those that had sufficient number of paired samples. As described in the article (Supplementary Figure 10), it is necessary to have at least 12-13 samples per class (e.g. tumor and normal) to obtain significant isoform-pairs. This discarded uterine cancer (UCEC), for which we only had 6 paired-samples available. For BLCA we had 16 paired-samples. However, from the URSA analysis we noticed that 10 of the normal BLCA samples were predicted to be similar to smooth muscle, whereas the other 6 were predicted to be similar to epithelial tissue and more similar to the tumor samples. Moreover, the smooth muscle like samples showed overexpression of mesenchymal markers, whereas the others had overexpression of epithelial markers, the latter being more similar to the tumor paired samples (data not shown). The analysis of splicing changes between tumor and normal samples including all paired-samples was thus yielding mostly changes related to epithelial-mesenchymal transition (EMT) (Warzecha et al. 2010). These mesenchymal-like samples may be indicative of a contamination from smooth muscle so we removed them from the analysis. Accordingly, we were left with just 6 patients; thus we decided to discard BLCA from further analysis.

For isoform expression, the abundance of every isoform in each sample was calculated in terms of transcripts per million (TPM) (Li et al. 2010) from the isoform-estimated read counts provided by TCGA and the isoform lengths from their annotation (UCSC genes – June 2011). No further normalization on the TPM values was performed. For each isoform i in gene G, the PSI was calculated as

$$PSI_i = \frac{TPM_i}{\sum_{\forall k \in G} TPM_k},$$

Genes with one single isoform, or no HUGO ID, were removed from further analyses.

The iso-kTSP algorithm

The iso-kTSP algorithm is implemented in Java. Software and documentation are available at https://bitbucket.org/regulatorygenomicsupf/iso-ktsp. The algorithm is based on the relative expression reversals of isoforms. It implements the principles of the kTSP algorithm (Geman et al. 2004, Tan et al. 2005) applied to isoforms from the same gene, but with a different scoring function (see below). It reads the TPM values for isoform expression and it stores the rankings in each sample for a number of samples from two possible classes, C_m, $m=1,2$. For every pair of isoforms $I_{g,i}$ and $I_{g,j}$ in each gene g, iso-kTSP calculates a score based on the frequencies of the two possible relative orders in both classes:

$$S_1(I_{g,i}, I_{g,j}) = P(I_{g,i} > I_{g,j} \mid C_1) + P(I_{g,i} < I_{g,j} \mid C_2) - 1$$

Where $P(I_{g,i} > I_{g,j} | C_1)$ and $P(I_{g,i} < I_{g,j} | C_2)$ are the frequencies at which the isoform $I_{g,i}$ appears later than, or before, $I_{g,j}$ in the expression ranking of class C_1 or C_2, respectively. Score S_1 provides an estimate of how probable the two isoforms are to change relative order between the two classes. Accordingly, the higher the S_1 score, the more consistent the isoform change between classes. Our definition of S_1 differs from the one used in (Tan et al. 2005) to account for the fact that in RNA-Seq there are many transcripts with zero reads, hence the expression ranking is not always strictly monotonic. Furthermore, to avoid possible ties, a second score is used, S_2 , which is based on the average rank difference per class C_m for each isoform pair, as defined previously (Tan et al. 2005). Defining $R(I_{g,i} | S_a, C_m)$ as the rank of isoform $I_{g,i}$ in sample S_a and class C_m, the average rank difference between two isoforms in a given class is calculated as

$$g(I_{g,i}, I_{g,j} \mid C_m) = \frac{1}{|C_m|} \sum_a \left(R(I_{g,i} \mid S_a, C_m) - R(I_{g,j} \mid S_a, C_m) \right)$$

where $|C_m|$ denotes the number of samples in class C_m. The score S_2 for an isoform pair is then defined as previously (Tan et al. 2005):

$$S_2(I_{g,i}, I_{g,j}) = \left| g(I_{g,i}, I_{g,j} \mid C_1) - g(I_{g,i}, I_{g,j} \mid C_2) \right|$$

S_2 provides an estimate of the magnitude of the expression reversal for a pair of isoforms from the same gene between the two classes. All possible isoform pairs are sorted by the S_1 score and in the case of a tie, by the S_2 score. Even though the isoform expression ranking is global, only pairs of isoforms from the same gene are considered. Additionally, only one isoform-pair per gene is chosen, hence a gene can only be listed once in the ranking of isoform pairs.

The classification is given in terms of k isoform-pairs, where each isoform-pair provides a rule determined by the order that maximizes the score S_1. For instance, a pair of isoforms $I_{g,i}$ and $I_{g,j}$ for which $P(I_{g,i} > I_{g,j} \mid C_1) > P(I_{g,i} < I_{g,j} \mid C_1)$ and $P(I_{g,i} < I_{g,j} \mid C_2) > P(I_{g,i} > I_{g,j} \mid C_2)$ would define the following rule

$$I_{g,j} < I_{g,i} \Rightarrow C_1$$
$$\text{otherwise} \Rightarrow C_2$$

The semantics of an isoform-pair rule is that if the first isoform is lower in expression than the second, the prediction is C_1 (the first class label in the input file), and in other cases the prediction is C_2. In general, we are using C_1=”normal” and C_2=”tumor”, hence:

$$\text{rule: } I_{g,1}, I_{g,2}$$
$$I_{g,1} < I_{g,2} \Rightarrow \text{normal}$$
$$\text{else} \quad \Rightarrow \text{tumor}$$

Accordingly, we will call $I_{g,1}$ the “tumor isoform” and $I_{g,2}$ the “normal isoform”. This semantics applies similarly when comparing a tumor subtype with the rest of tumor types. For instance, in the case of the comparison of basal-like breast tumors with the rest, one rule would read as:

$$\text{rule: } I_{g,1}, I_{g,2}$$
$$I_{g,1} < I_{g,2} \Rightarrow \text{basal}$$
$$\text{else} \quad \Rightarrow \text{non-basal}$$

And as before, we will call $I_{g,2}$ the “basal isoform” and $I_{g,1}$ the “non-basal isoform”.

The classification of a new sample is performed by evaluating each isoform-pair rule against the ranking of isoform expression of this new sample. Given k rules, the classifier selects for each isoform-pair rule the class for which the data fulfills the rule.

The final decision for classification is established by simple majority voting, by selecting the most voted class from the k rules. In order to avoid ties in this voting, k is always odd. For instance, for k=3, we could have:

$$\left.\begin{array}{l}\text{rule 1: } \Rightarrow \text{ tumor}\\ \text{rule 2: } \Rightarrow \text{ tumor}\\ \text{rule 3: } \Rightarrow \text{ normal}\end{array}\right\}\text{classification : tumor}$$

The optimal number k of isoform pairs in the classifier, k_{opt}, is calculated by performing cross-validation on the training set. The samples are split into training and testing sets, each containing the same number of samples from each class. For our analyses, at each step of the cross-validation the testing is done at a single pair of tumor-normal samples, training on the rest of the paired samples; hence, at the end of the cross-validation every tumor-normal pair is tested once. At each step of the cross-validation, a model is built for various values of k odd, up to a value k_{max} input as parameter (k_{max} = 50 in our analysis). For each k, the accuracy of the model is evaluated against the test set, where

$$\text{accuracy} = \frac{TP+TN}{TP+TN+FN+FP}$$

and where TP, TN, FN and FP are the true positives, true negatives, false negatives and false positives, respectively. This accuracy value is symmetric with respect to the choice of either class as reference for positive cases. After cross-validation, the optimal number of pairs, k_{opt}, is taken to be the minimum k with the highest average accuracy, where the average is calculated across all cross-validation steps. Finally, using the global ranking of isoform-pairs, now calculated on the entire dataset, the top k_{opt} pairs are then reported as the classification model. These k_{opt} pairs are the isoforms that most consistently and with greatest average relative difference (e.g. tumor and normal, two cancer subtypes, etc.) separate the two classes. Additionally, the iso-kTSP method also reports the individual performances of each isoform-pair in terms of the Information Gain (IG) measure, which provides an estimate of the discriminating power. IG for an attribute A is defined as the expected reduction in entropy caused by partitioning the set of examples S according to the value t of the attribute A:

$$IG(A) = H(S) - H(S,t,A)$$

Where H is Shannon's entropy according to the normal and tumor classes:

$$H(S) = -p_N \log_2 p_N - p_T \log_2 p_T$$

where p_N and p_T are the proportion of normal and tumor samples. Since we are always using S to be a balanced set according to the classes, we always have $H(S) = 1$,. In our case, the attribute is an isoform-pair and the partitioning is whether the isoform-pair rule is fulfilled. Hence:

$$H(S,t,A) = \frac{|S_+|}{|S|} H(S_+) + \frac{|S_-|}{|S|} H(S_-)$$

where S_+ and S_- are the subsets of examples that fulfil or not the isoform-pair rule, respectively; and $H(S_+)$ and $H(S_-)$ are the entropy values according to the normal and tumor classes for each of these subsets:

$$H(S_+) = -p_{+,N} \log_2 p_{+,N} - p_{+,T} \log_2 p_{+,T}$$
$$H(S_-) = -p_{-,N} \log_2 p_{-,N} - p_{-,T} \log_2 p_{-,T}$$

where $p_{+,N}$ and $p_{+,T}$ are the proportion of samples that fulfil the isoform-pair rule that are normal and tumor, respectively, which we can re-write as:

$$p_{+,N} = \frac{|S_{+,N}|}{|S_+|}, \quad p_{+,T} = \frac{|S_{+,T}|}{|S_+|}$$

and similarly for $p_{-,N}$ and $p_{-,T}$. Considering that the isoform-pair rule is defined as true for tumor samples, it follows that $|S_{+,T}|$ is the same as the true positives (TP), $|S_{+,N}|$ is equal to the false positives (FP), and similarly, $|S_{-,T}|$ is the same as the false negatives (FN), and $|S_{-,N}|$ is equal to the true negatives (TN). Defining

$$PPV = \frac{TP}{TP+FP}, \; FDR = \frac{FP}{TP+FP}$$
$$NPV = \frac{TN}{TN+FN}, \; FNR = \frac{FN}{TN+FN}$$

where PPV, FDR, NPV and FNR are the positive predictive value, false discovery rate, negative predictive value and false negative rate, respectively, the information gain defined above can be thus rewritten as:

$$IG = 1 - \frac{|S_+|}{|S|}\left(-PPV\log_2 PPV - FDR\log_2 FDR\right)$$
$$-\frac{|S_-|}{|S|}\left(-FNR\log_2 FNR - NPV\log_2 NPV\right)$$

$-$

where $S = S_+ \cup S_-$. Thus, provided the measures of TP, FP, FN and TN for a given isoform-pair, we can calculate the associated information gain for that isoform pair using this formula. This provides in a single number a measure of the separation power of this isoform-pair rule, which can be then compared to the other isoform-pairs.

Z-score calculation for score S_I and Information Gain

To calculate a Z-score for score S_I and the information gain (IG), we carried out an iso-kTSP analysis, where we calculated the single pair performance for all genes used in the analysis. That is, asking the algorithm to provide a ranking of isoform pairs for all genes, which provides the best isoform-pair per gene, with the corresponding individual IG and S_I values. This resulted in 14630 values for S_I and IG, which were used to calculate mean values and standard deviation of the population. We then subtracted the mean from each individual value and divided it by the standard deviation, resulting in a standardized Z-score.

Permutation analysis

Significance of the computed isoform switches was evaluated with iso-kTSP program using permutation analysis. Namely, labels from the two classes (e.g. tumor vs normal) were shuffled 1000 times. For each shuffling step, the iso-kTSP algorithm was re-run and the top-scoring isoform-pair was selected. The distribution of the 1000 top-scoring pairs from the shufflings provides an estimate of the expected recurrence of isoform changes obtained by chance (Supplementary Figure 10). An isoform-pair is significant if the information gain and Score S_I are both larger than any of the values obtained from the 1000 shufflings.

Blind tests

A blind test of each model was carried out on the set of samples that were not used for cross-validation. These are all the unpaired tumor samples. For the analysis of the cancer subtypes, however, the overall accuracy is estimated on the complete set of tumor samples. In all these tests, we measure the accuracy as described above, calculating the proportion of samples correctly labeled by the classifier, as well as counting the number of correct votes per sample.

Anticorrelation

For each isoform pair in a rule we calculated the Spearman correlation of the PSI values for the paired tumor and normal samples. Those pairs with correlation value Spearman $R < -0.8$, i.e. anticorrelation, were considered proper isoform switches where the most abundant isoform in each sample is one of the isoforms in the pair.

Analysis of Subtypes

We applied the iso-kTSP algorithm to study the four BRCA subtypes as classified by TCGA (TCGA 2012): luminal A, luminal B, basal and Her2+, which have 231, 127, 98 and 58 samples, respectively. For each subtype, 100 random subsets were sampled, selecting 45 from a given subtype, and 15 from each of the other subtypes to maintain balanced sets. For each one of the 100 iterations, a iso-kTSP model was built as described above with $k = 50$; and the significance of the predicted isoform changes were computed using permutation analysis as described above. Finally, for each subtype and for each isoform pair, the proportion of iterations in which it is used in a model and the proportion of iterations in which it is significant is reported.

We also analysed the four LUSC subtypes: basal, classical, primitive and secretory (Wilkerson et al. 2010), with 41, 57, 25 and 38 samples, respectively. Similarly to the BRCA subtypes, 100 random samples for each subtype were considered, each time selecting 24 from one subtype and 8 from each other three subtypes. For each of the 100 iterations a iso-kTSP model was built with $k = 50$, and the significance estimated by permutation analysis.

COAD non-paired tumor samples were separated into hypermutated or non-hypermutated according to the number of mutations per sample. Using all mutation

types (classified by TCGA as functional or non-functional), samples with more than 250 mutations were classified as hypermutated and those with less than 250 mutations were classified as non-hypermutated; leaving 59 and 130 samples, respectively. Similarly as before, both COAD subtypes were compared by sampling 100 times 40 samples from either subtype, and by building the iso-kTSP model at each iteration and computing the significance by permutation analysis.

Analysis of somatic mutations

We downloaded somatic mutation data from TCGA. Download locations:

(1)/brca/gsc/genome.wustl.edu/illuminaga_dnaseq_curated/mutations/genome.wustl.edu_BRCA.IlluminaGA_DNASeq_curated.Level_2.1.1.0.tar.gz
(1)/brca/gsc/genome.wustl.edu/illuminaga_dnaseq/mutations/genome.wustl.edu_BRCA.IlluminaGA_DNASeq.Level_2.5.3.0.tar.gz
(1)/coad/gsc/hgsc.bcm.edu/solid_dnaseq/mutations/hgsc.bcm.edu_COAD.SOLiD_DNASeq.Level_2.1.7.0.tar.gz
(1)/coad/gsc/hgsc.bcm.edu/illuminaga_dnaseq/mutations/hgsc.bcm.edu_COAD.IlluminaGA_DNASeq.Level_2.1.5.0.tar.gz
(1)/hnsc/gsc/broad.mit.edu/illuminaga_dnaseq/mutations/broad.mit.edu_HNSC.IlluminaGA_DNASeq.Level_2.1.0.0.tar.gz
(1)/kich/gsc/hgsc.bcm.edu/mixed_dnaseq_curated/mutations/hgsc.bcm.edu_KICH.Mixed_DNASeq_curated.Level_2.1.0.0.tar.gz
(1)/kich/gsc/broad.mit.edu/illuminaga_dnaseq/mutations/broad.mit.edu_KICH.IlluminaGA_DNASeq.aux.1.0.0.tar.gz
(1)/kich/gsc/broad.mit.edu/illuminaga_dnaseq/mutations/broad.mit.edu_KICH.IlluminaGA_DNASeq.Level_2.1.0.0.tar.gz
(1)/kich/gsc/hgsc.bcm.edu/illuminaga_dnaseq/mutations/hgsc.bcm.edu_KICH.IlluminaGA_DNASeq.Level_2.1.2.0.tar.gz
(1)/kirc/gsc/hgsc.bcm.edu/mixed_dnaseq/mutations/hgsc.bcm.edu_KIRC.Mixed_DNASeq.Level_2.1.2.0.tar.gz
(1)/kirc/gsc/broad.mit.edu/illuminaga_dnaseq/mutations/broad.mit.edu_KIRC.IlluminaGA_DNASeq.Level_2.1.5.0.tar.gz
(1)/kirc/gsc/broad.mit.edu/illuminaga_dnaseq/mutations/broad.mit.edu_KIRC.IlluminaGA_DNASeq.aux.1.2.0.tar.gz
(1)/luad/gsc/broad.mit.edu/illuminaga_dnaseq/mutations/broad.mit.edu_LUAD.IlluminaGA_DNASeq.Level_2.0.4.0.tar.gz
(1)/lusc/gsc/broad.mit.edu/illuminaga_dnaseq/mutations/broad.mit.edu_LUSC.IlluminaGA_DNASeq.Level_2.100.1.0.tar.gz
(1)/lusc/gsc/broad.mit.edu/illuminaga_dnaseq/mutations/broad.mit.edu_LUSC.IlluminaGA_DNASeq.aux.1.2.0.tar.gz
(1)/prad/gsc/hgsc.bcm.edu/illuminaga_dnaseq_automated/mutations/hgsc.bcm.edu_PRAD.IlluminaGA_DNASeq_automated.Level_2.1.0.0.tar.gz
(1)/prad/gsc/broad.mit.edu/illuminaga_dnaseq_curated/mutations/broad.mit.edu_PRAD.IlluminaGA_DNASeq_curated.Level_2.1.2.0.tar.gz
(1)/prad/gsc/broad.mit.edu/illuminaga_dnaseq/mutations/broad.mit.edu_PRAD.IlluminaGA_DNASeq.Level_2.1.3.0.tar.gz
(1)/prad/gsc/broad.mit.edu/illuminaga_dnaseq/mutations/broad.mit.edu_PRAD.IlluminaGA_DNASeq.aux.1.0.0.tar.gz
(1)/thca/gsc/hgsc.bcm.edu/illuminaga_dnaseq_automated/mutations/hgsc.bcm.edu_THCA.IlluminaGA_DNASeq_automated.Level_2.1.1.0.tar.gz
(1)/thca/gsc/broad.mit.edu/illuminaga_dnaseq/mutations/broad.mit.edu_THCA.IlluminaGA_DNASeq.aux.1.5.0.tar.gz
(1)/thca/gsc/broad.mit.edu/illuminaga_dnaseq/mutations/broad.mit.edu_THCA.IlluminaGA_DNASeq.Level_2.1.5.0.tar.gz

where, as before,

(1) = https://tcga-data.nci.nih.gov/tcgafiles/ftp_auth/distro_ftpusers/anonymous/tumor

The number of samples with mutation data, and with both mutation data and RNA-Seq data are given in Supplementary Table 2 for each cancer type. For the purpose of finding associations, we simply considered whether a gene or the isoform-pair of the gene in a given sample either has or does not have mutations. Although most of the mutational data in TCGA comes from exome sequencing data, there are cases where it comes from whole genome sequencing. Accordingly, in order to homogenize the mutation sampling, we considered mutations that fall on exon regions or in the 100 nt upstream or downstream flanking regions. We carried out several different tests for the association of somatic mutations and isoform switches, which are described below.

Jaccard index

Given the samples with one or more mutations *M* on the transcripts involved in the switch, and the samples with the isoform switch *S*, a Jaccard index *J* for the association of these two variables was calculated:

$$J = \frac{|M \cap S|}{|M \cup S|}$$

This index was calculated using the clujaccard function from the R package fpc (http://cran.r-project.org/web/packages/fpc) (Hennig 2014). A Z-score from the Jaccard index was calculated by comparison to genes with alternative splicing isoforms and with similar median isoform length. Genes used in the iso-kTSP analysis were ranked according to the absolute length difference of the median isoform length with the given gene. Lengths were calculated as the sum of the component exons from the TCGA annotation (UCSC genes, June 2011), which is equivalent to the length of the projection of the exons from the gene to the genome. The top 100 with the smallest length difference were selected, and two of its isoforms were chosen randomly. For each of these matched controls the Jaccard index was calculated as before. A Z-score was then calculated based on the mean and standard deviation of the Jaccard index from the 100 matched controls. The above analysis was also repeated using only mutations that fall on exons and that affect the protein sequence (Frame_Shift_Del, Frame_Shift_Ins, In_Frame_Del, In_Frame_Ins,

Missense_Mutation, Nonsense_Mutation, Nonstop_Mutation) with very similar results.

Mutual information

The mutual information was calculated using the presence or absence of mutations and switches in each sample, using the mi.empirical function from the entropy package of R (Hausser et al. 2009). Using mutations falling on the isoforms undergoing the switch, the mutual information values correlate with the Jaccard index (Spearman R = 0.9) and with the Jaccard z-scores (Spearman R=0.7) calculated above. This analysis was also performed using mutations falling on the entire gene locus with very similar results.

Wilcox-test of delta-PSI values

For each isoform-pair we calculated a delta-PSI value for each tumor sample by subtracting the PSI of the tumor isoform from the PSI of the normal isoform. We then separated the samples into two classes, based on the presence or absence of any mutation in the isoform pair. We carried out a Mann-Whitney test to check if there are significant differences in the distribution of delta-PSI values between the two populations, and used the Benjamini-Hochberg method to correct the p-values for multiple testing. The same analysis was also performed using mutations on the entire gene locus with very similar results.

Fisher test

A Fisher test was performed per isoform-pair by building a 2x2 contingency table with the number of tumor samples with or without mutations and the number of tumor samples with or without the corresponding switch. None of the isoform switches had a significant association according to this test after correcting for multiple testing with the Benjamini-Hochberg method. The same analysis was also performed using mutations on the entire gene locus with very similar results.

Mutual exclusion analysis of functional mutations and switches

A mutual-exclusion between isoform switches and protein-affecting mutations was measured as follows: given the number of samples having an isoform switch and no

mutation (n_{10}), and those having a mutation but no isoform switch (n_{01}), a mutual-exclusion score was defined to be:

$$2\frac{\min(n_{10}, n_{01})}{N}$$

where N is the total number of samples. A z-score was calculated similarly as above for the Jaccard index.

Reactome analysis

For the Reactome (Croft et al. 2014) enrichment analysis we used the ReactomePA package from Bioconductor (Yu 2014) with default values, only changing the "universe" parameter, where we used the 14630 Entrez gene IDs that were used for the iso-kTSP analysis (all the genes present in the annotation with multiple alternative splicing isoforms).

GO enrichment analysis

GO-terms were tested for enrichment in the three ontologies: Biological Process, Cellular Component and Molecular Function. The GOstats package (Falcon and Gentleman 2007) was used for the test, with a P-value cutoff of 0.05, odds-ratio > 2 and at least 5 genes. The background set was the gene set used for the iso-kTSP analysis and the "conditional" option was used in the test to calculate the significance of a parent term, if its children were significant.

Supplementary Files description:

Supplementary File 1 (SupplementaryFile1.zip)

This is a compressed file with the lists of patient-samples used for the analyses. Each sample is described by a short ID, which was also used in the data tables. The sample ID is the participant ID part of the TCGA barcode, with an additional N or T character at the end, meaning normal or tumor sample, respectively. We include the patients that had RNA-Seq data and the patients for which we had mutation data.

Supplementary File 2 (SupplementaryFile2.zip)

This is a compressed file with all the iso-kTSP models described in the paper, ready to be used and tested with the iso-kTSP algorithm. The format is plain text, where each isoform-pair of the model is given per line, separated by a tab. Each isoform is specified by an ID composed of the gene-ID and transcript-ID separated by ",". For instance the LUAD model is:

QKI|9444,uc003qui.2	QKI|9444,uc003qug.2
NUMB|8650,uc001xnz.1	NUMB|8650,uc001xoa.1
MTA3|57504,uc002rsr.2	MTA3|57504,uc002rsp.1
SLC39A8|64116,uc003hwb.1	SLC39A8|64116,uc011ceo.1
ESYT2|57488,uc003wod.1	ESYT2|57488,uc003wob.1
PRMT2|3275,uc002zjy.2	PRMT2|3275,uc002zjz.1
ISLR|3671,uc002axh.1	ISLR|3671,uc002axg.1

Supplementary File 3 (SupplementaryFile3.zip)

This compressed file includes the GFF (version GFF3) files of the tumor and normal isoforms for the predictive models (cancer types and subtypes), for all the significant isoform-pairs and for the isoform switches.

Supplementary File 4 (SupplementaryFile4.zip)

This contains two plain text, tab-separated files with all the information derived for the isoform switches and for the significant isoform pairs. The files contain a header. The column description for the isoform-pair list is as follows:

	**Header**	**Explanation**
1	cancer	Cancer type (BRCA, etc..)
2	gene_name	HUGO gene ID
3	tumor	Tumor isoform ID, format: gene_name,transcript_id
4	normal	Normal isoform ID, format: gene_name,transcript_id
5	Ig	Information Gain of the isoform-pair
6	score1	Score S_1 of the isoform-pair

Each switch is characterized by the gene name and the pair of isoforms (columns 2,3 and 4).

The column description of the isoform switch list is as follows:

	Header	Explanation
1	Cancer	Cancer type (BRCA, etc..)
2	gene_name	HUGO gene ID
3	tumor	Tumor isoform ID, format: gene_name,transcript_id
4	Normal	Normal isoform ID, format: gene_name,transcript_id
5	Category	One of the following:<br>No CDS (no CDS in the annotation),<br>No normal CDS (the normal isoform has no CDS),<br>No tumor CDS (The tumor isoform has no annotated CDS),<br>No protein affected, (no change in the protein product),<br>Protein affected (the switch changes protein product)
6	Ig	Information Gain of the switch
7	score1	Score S_1 of switch
8	Ig_zscore	Z-score of switch information gain
9	score1_zscore	Z-score of switch Score S_1
10	corr	Spearman correlation of isoform-pair PSI values in the paired samples
11	TinN	Average expression TPM of tumor isoform in normal samples
12	TinT	Average expression TPM of tumor isoform in tumor samples
13	NinN	Average expression TPM of normal isoform in normal samples
14	NinT	Average expression TPM of normal isoform in tumor samples
15	Isonum	Total number of annotated isoforms in gene
16	freqAG	Frequency of samples with any mutation in the full gene
17	freqAI	Frequency of samples with any mutation in the isoform switch
18	freqFG	Frequency of samples with functional mutation in the full gene
19	freqFI	Frequency of samples with functional mutation in the isoform switch
20	dpsiAG	Mann-Whitney test p-value for comparing PSI values of tumor samples with and without any mutation in the full gene
21	dpsiAI	Mann-Whitney test p-value for comparing PSI values of tumor samples with and without any mutation in the isoform switch
22	freq_switches	Frequency of samples with the isoform switch
23	fisherAG	Fisher test p-value comparing the association of isoform switches and any type of mutations along the full gene in the samples
24	fisherAI	Fisher test p-value comparing the association of isoform switches and any type of mutations along the isoform switch in the samples
25	mutinfAG	Mutual information score of the association of isoform switches and any type of mutations along the full gene in the samples
26	mutinfAI	Mutual information score of the association of isoform switches and any type of mutations along the isoform switch in thea samples
27	jaccardAG	Jaccard index of the association of isoform switches and any type of mutations along the full gene in the samples
28	jaccardAI	Jaccard index of the association of isoform switches and any type of mutations along the isoform switch in the samples
29	jaccardFG	Jaccard index of the association of isoform switches and functional mutations along the full gene in the samples
30	jaccardFI	Jaccard index of the association of isoform switches and functional mutations along the isoform switch in the samples
31	funvalFG	Mutual exclusion score of the association of isoform switches and functional mutations along the full gene in the samples
32	funvalFI	Mutual exclusion score of the association of isoform switches and functional mutations along the isoform switch in the samples
33	jaccardAG_Z	Z-score of the jaccardAG score
34	jaccardAI_Z	Z-score of the jaccardAI score
35	jaccardFG_Z	Z-score of the jacardFG score
36	jaccardFI_Z	Z-score of the jaccardFI score
37	funvalFG_Z	Z-score of the funvalFG score
38	funvalFI_Z	Z-score of the funvalFI score
39	dpsiAG_adj	Benjamini - Hochberg adjusted dpsiAG p-value
40	dpsiAI_adj	Benjamini - Hochberg adjusted dpsiAI p-value
41	tumoriso_transcript	Tumor isoform mRNA length
42	tumoriso_genomic	Tumor isoform pre-mRNA length
43	normaliso_transcript	Normal isoform mRNA length
44	normaliso_genomic	Normal isoform pre-mRNA length

Data availability

Data is available at DOI: http://dx.doi.org/10.6084/m9.figshare.1061917

References

Croft D, Mundo AF, Haw R, Milacic M, Weiser J, Wu G, Caudy M, Garapati P, Gillespie M, Kamdar MR, Jassal B, Jupe S, Matthews L, May B, Palatnik S, Rothfels K, Shamovsky V, Song H, Williams M, Birney E, Hermjakob H, Stein L, D'Eustachio P (2014) The Reactome pathway knowledgebase. Nucleic Acids Research 1 January 2014; 42(D1):D472-D477

Falcon S, Gentleman R. (2007) Using GOstats to test gene lists for GO term association. Bioinformatics 23(2):257-8

Geman D, d'Avignon C, Naiman DQ, Winslow RL. Classifying gene expression profiles from pairwise mRNA comparisons. Stat Appl Genet Mol Biol. 2004;3:Article19.

Hennig C (2014). fpc: Flexible procedures for clustering. R package version 2.1-9. http://CRAN.R-project.org/package=fpc

Hausser J, & Strimmer K. 2009. Entropy inference and the James-Stein estimator, with application to nonlinear gene association networks. J. Mach. Learn. Res. 10: 1469-1484

Lee YS, Krishnan A, Zhu Q, Troyanskaya OG. Ontology-aware classification of tissue and cell-type signals in gene expression profiles across platforms and technologies. Bioinformatics. 2013 Dec 1;29(23):3036-44.

Li B, Ruotti V, Stewart R, Thomson J, Dewey C. (2010). RNA-Seq gene expression estimation with read mapping uncertainty. Bioinformatics 26(4):493-500.

Tan AC, Naiman DQ, Xu L, Winslow RL, Geman D. Simple decision rules for classifying human cancers from gene expression profiles. Bioinformatics. 2005 Oct 15;21(20):3896-904.

The Cancer Genome Atlas Network (2012c) Comprehensive molecular portraits of human breast tumours. *Nature.* 490(7418):61-70

Warzecha CC, Jiang P, Amirikian K, Dittmar KA, Lu H, Shen S, Guo W, Xing Y, Carstens RP. An ESRP-regulated splicing programme is abrogated during the epithelial-mesenchymal transition. EMBO J. 2010 Oct 6;29(19):3286-300.

Wilkerson MD, Yin X, Hoadley KA, Liu Y, Hayward MC, Cabanski CR, Muldrew K, Miller CR, Randell SH, Socinski MA, Parsons AM, Funkhouser WK, Lee CB, Roberts PJ, Thorne L, Bernard PS, Perou CM, Hayes DN. Lung squamous cell carcinoma mRNA expression subtypes are reproducible, clinically important, and correspond to normal cell types. Clin Cancer Res. 2010 Oct 1;16(19):4864-75.

Yu G. (2014) *ReactomePA: Reactome Pathway Analysis*. R package version 1.8.1. http://www.bioconductor.org/packages/release/bioc/html/ReactomePA.html
